# Growth‐mediated negative feedback shapes quantitative antibiotic response

**DOI:** 10.15252/msb.202110490

**Published:** 2022-09-20

**Authors:** S Andreas Angermayr, Tin Yau Pang, Guillaume Chevereau, Karin Mitosch, Martin J Lercher, Tobias Bollenbach

**Affiliations:** ^1^ Institute for Biological Physics University of Cologne Cologne Germany; ^2^ Institute of Science and Technology Austria Klosterneuburg Austria; ^3^ Institute for Computer Science Heinrich Heine University Düsseldorf Düsseldorf Germany; ^4^ Department of Biology Heinrich Heine University Düsseldorf Düsseldorf Germany; ^5^ INSA de Strasbourg Strasbourg France; ^6^ Genome Biology Unit European Molecular Biology Laboratory (EMBL) Heidelberg Germany; ^7^ Center for Data and Simulation Science University of Cologne Cologne Germany; ^8^ Present address: CeMM Research Center for Molecular Medicine of the Austrian Academy of Sciences Vienna Austria

**Keywords:** antibiotics, dihydrofolate reductase (DHFR), dose–response curve, feedback loops, resource allocation model, Microbiology, Virology & Host Pathogen Interaction, Pharmacology & Drug Discovery

## Abstract

Dose–response relationships are a general concept for quantitatively describing biological systems across multiple scales, from the molecular to the whole‐cell level. A clinically relevant example is the bacterial growth response to antibiotics, which is routinely characterized by dose–response curves. The shape of the dose–response curve varies drastically between antibiotics and plays a key role in treatment, drug interactions, and resistance evolution. However, the mechanisms shaping the dose–response curve remain largely unclear. Here, we show in *Escherichia coli* that the distinctively shallow dose–response curve of the antibiotic trimethoprim is caused by a negative growth‐mediated feedback loop: Trimethoprim slows growth, which in turn weakens the effect of this antibiotic. At the molecular level, this feedback is caused by the upregulation of the drug target dihydrofolate reductase (FolA/DHFR). We show that this upregulation is not a specific response to trimethoprim but follows a universal trend line that depends primarily on the growth rate, irrespective of its cause. Rewiring the feedback loop alters the dose–response curve in a predictable manner, which we corroborate using a mathematical model of cellular resource allocation and growth. Our results indicate that growth‐mediated feedback loops may shape drug responses more generally and could be exploited to design evolutionary traps that enable selection against drug resistance.

## Graphical abstract

Growth‐rate dependent sensitivity of *E. coli* to the antibiotic trimethoprim leads to a negative feedback loop that explains the extreme shallowness of the dose‐response curve. This feedback loop is mediated by the regulation of the drug target dihydrofolate reductase (DHFR).

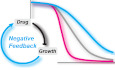

## Introduction

Dose–response curves are a central concept in systems biology and essential for understanding emergent nonlinear phenomena at different scales. A prime example is bacterial gene regulation where cooperativity of transcription factor binding to promoter regions governs the steepness of dose–response curves that characterize gene expression as a function of transcription factor concentration (Bintu *et al*, 2005). Negative feedback can reduce the steepness of dose–response curves of gene expression, i.e., change their shape from sigmoidal to linear (Nevozhay *et al*, 2009). The steepness of transcription factor dose–response curves ultimately determines whether feedback loops in genetic circuits can produce biologically relevant functions such as bistability or oscillations (Elowitz & Leibler, 2000; Gardner *et al*, 2000). At the population level, the bacterial response to antibiotics is captured by similar dose–response curves that quantify the dependence of growth rate on drug concentration. Antibiotic dose–response curves are routinely measured to characterize antibiotic susceptibility via the minimal inhibitory concentration (MIC) or the concentration leading to 50% growth inhibition (IC_50_), two classic quantities to describe antibiotic efficacy. However, the quantitative shape of the antibiotic dose–response curve – especially its steepness – and its implications are underappreciated.

**Figure 1:**
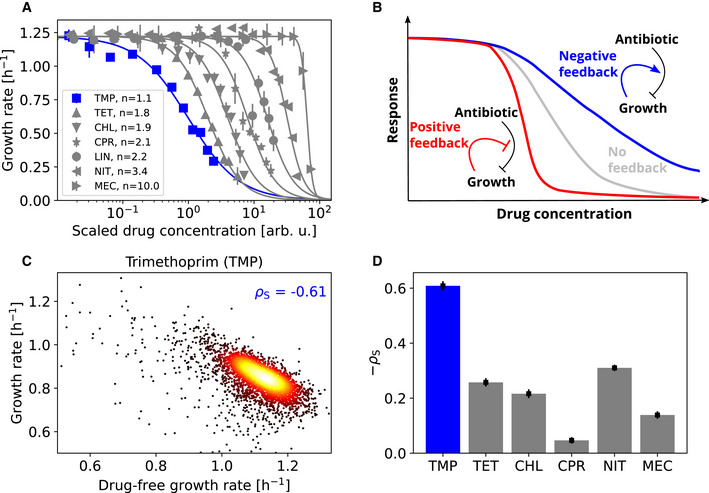
Trimethoprim exhibits an extremely shallow dose response curve and its efficacy correlates strongly with growth rate compared to other antibiotics ADose–response curves (normalized growth rate as a function of drug concentration) for different antibiotics. Growth rate was measured via optical density measurements over time (Materials and Methods). Antibiotics used: Trimethoprim (TMP), tetracycline (TET), chloramphenicol (CHL), ciprofloxacin (CPR), lincomycin (LIN), nitrofurantoin (NIT), and mecillinam (MEC). The TMP dose–response curve (dark blue) is by far the shallowest. Lines are fits of the Hill function $\frac{g\left(c\right)}{g\left(0\right)}=\frac{1}{1+{\left(\frac{c}{IC_{50}}\right)}^{n}}$ to the data. Drug concentrations were arbitrarily rescaled to better visualize dose–response curve steepness; for unscaled dose–response curves, see Appendix Fig S13. Error bars show standard deviation of 12 biological replicates.BSchematic: Effect of growth‐mediated feedback loops on dose–response curves. Negative feedback (blue) renders the dose–response curve shallower than in the absence of feedback (gray); positive feedback (red) steepens the dose–response curve.CDensity scatterplot showing growth response to TMP versus normalized drug‐free growth rate for 3,913 gene deletion strains (Baba *et al*, 2006); these are essentially all viable gene deletion strains in *E. coli*, no selection of strains was made. These gene deletion strains exhibit diverse growth rates, offering an unbiased way to test the relation between the drug‐free growth rate and the response to antibiotics. Response is defined as growth rate in the presence of TMP normalized to the drug‐free growth rate of the respective deletion strain. TMP was used at a fixed concentration that inhibits wild type growth by about 30% (Chevereau *et al*, 2015). Spearman correlation coefficient $\rho_{s}$ is shown.DBar chart showing negative Spearman correlation coefficients $-\rho_{s}\;$compared across antibiotics (Appendix Fig S1). Error bars show bootstrap standard error of $\rho_{s}$. TMP (blue) exhibits by far the strongest negative correlation, indicating the existence of a particularly strong growth‐mediated negative feedback loop for this antibiotic. Source data are available online for this figure.

The steepness of the dose–response curve varies drastically between antibiotics. For many antibiotics, the growth rate drops gradually from high to low as the drug concentration is increased (Fig 1A); in particular, this is the case for antibiotics targeting DNA replication at the gyrase (e.g. ciprofloxacin) or antibiotics targeting translation at the ribosome (e.g. tetracycline). Beta‐lactams like mecillinam (an antibiotic targeting cell wall biosynthesis at a penicillin binding protein) have extremely steep dose–response curves where just a slight relative increase in drug concentration – by about two‐fold – causes an abrupt transition from full‐speed growth to near‐zero net growth (Fig 1A). At the other end of the spectrum, the folic acid synthesis inhibitor trimethoprim (TMP) has an extremely shallow dose–response curve (Palmer & Kishony, 2014; Chevereau *et al*, 2015; Rodrigues *et al*, 2016; Russ & Kishony, 2018): Reducing growth from full speed to zero with TMP requires a more than 100‐fold increase in drug concentration (Fig 1A). In general, dose–response curves are well approximated by Hill functions and the Hill slope n (“dose‐sensitivity”) is a quantitative measure of their steepness (Chou & Talalay, 1983; Regoes *et al*, 2004; Chevereau *et al*, 2015): TMP has n≈1.1, while most antibiotics fall in the range 1.8≤n≤3.5, and beta‐lactams such as mecillinam have n>6 (Fig 1A).

The steepness of the dose–response curve strongly affects the evolution of resistance by spontaneous mutations (Hermsen *et al*, 2012; Chevereau *et al*, 2015). Resistance mutations that slightly increase the MIC provide greater fitness benefits for drugs with a steep dose–response curve compared to drugs with a shallow curve, implying a greater chance to fix in the population. Thus, all else being equal, the rate of resistance evolution for a drug increases with the steepness of its dose–response curve – a trend that is observed in evolution experiments (Chevereau *et al*, 2015). This effect is strongest for drug concentrations near the IC_50_, occurring, for example, when populations of motile bacteria evolve resistance in spatial drug gradients where growth takes place primarily at a population front located in the region with drug concentrations that partially, but not completely, inhibit growth (Baym *et al*, 2016; Hol *et al*, 2016). Despite their fundamental relevance for resistance evolution and bacterial responses to antibiotics, the mechanisms that shape the dose–response curve are largely unknown.

Feedback loops mediated by growth rate may play a key role in shaping the dose–response curve (Deris *et al*, 2013; Greulich *et al*, 2015). The action of antibiotics affects bacterial growth but the inverse is also true: Slower growing bacteria are less rapidly killed by antibiotics targeting cell wall biosynthesis (beta‐lactams; Tuomanen *et al*, 1986; Lee *et al*, 2018) and non‐growing (persister) cells are fully protected from many antibiotics (Balaban *et al*, 2004), offering a possibility to evade antibiotic treatments. However, it is not clear if there is a more general relation between the drug‐free growth rate and common measures of antibiotic efficacy (such as MIC or IC_50_) that would generalize this trend across drug classes for both bacteriostatic and bactericidal antibiotics. Recent findings further suggest that antibiotic lethality depends on bacterial metabolic state rather than growth rate alone (Lopatkin *et al*, 2019). Slower growth caused by nutrient limitation affects the bacterial susceptibility to ribosome‐targeting antibiotics but the IC_50_ changes in opposite ways with increasing drug‐free growth rate for different ribosome inhibitors: it decreases for tetracycline and chloramphenicol but increases for streptomycin and kanamycin (Greulich *et al*, 2015). In engineered strains expressing a constitutive resistance gene, a positive feedback loop leads to high dose‐sensitivity and even bistability (i.e. co‐existence of growing and non‐growing cells) in the presence of the ribosome‐targeting antibiotic chloramphenicol (Deris *et al*, 2013). Positive feedback occurs as faster growth leads to the upregulation of the resistance enzyme, which in turn enables even faster growth. Growth‐mediated feedback loops could more generally explain the drastic differences in dose‐sensitivity between antibiotics (Fig 1A) with positive feedback producing higher (Deris *et al*, 2013) and negative feedback lower dose‐sensitivity. However, such feedback loops shaping the dose–response curve of sensitive wild‐type bacteria have not yet been characterized.

Here, we establish that negative growth‐mediated feedback produces an extremely shallow drug dose–response curve. Focusing on TMP, we vary bacterial growth rates by diverse environmental and genetic perturbations and show that, in contrast to most other antibiotics we investigated, slower growth generally lowers the susceptibility of *Escherichia coli* to this antibiotic. The molecular origin of this phenomenon lies in the expression of the drug target, which is upregulated in response to TMP but also when the growth rate is lowered by other means: TMP lowers growth, which in turn reduces susceptibility to TMP. We show that synthetically reversing this feedback loop can drastically steepen the dose–response curve. The negative feedback loop leads to a seemingly paradoxical situation where adding the antibiotic can even enhance growth under extreme nutrient limitations. It can be envisioned that such growth‐mediated feedback loops in drug responses could be used to design evolutionary traps that invert selection for resistance.

## Results

### Growth‐mediated feedback loops can affect the dose–sensitivity of drugs

We hypothesized that a growth‐mediated negative feedback loop could explain the shallowness of the dose–response curve of TMP. We focused on TMP because it had by far the shallowest dose–response of all antibiotics we investigated (Fig 1A). As an antibiotic, TMP lowers bacterial growth (by inhibiting dihydrofolate reductase, DHFR, encoded by *folA*). If a lower growth rate in turn protects bacteria from TMP, the resulting growth‐mediated negative feedback loop could lead to a shallow dose–response curve (Fig 1B). In contrast, for antibiotics where faster growth protects bacteria, positive growth‐mediated feedback leads to ultrasensitivity (Fig 1B) and can even produce bistability as previously reported (Elf *et al*, 2006; Deris *et al*, 2013). These results show that growth‐mediated feedback loops can affect the dose‐sensitivity of drugs in general.

### Slower growth generally lowers susceptibility to TMP and steepens its dose–response curve

To test experimentally whether negative growth‐mediated feedback underlies the shallow TMP dose–response curve, we varied the growth rate in several independent ways and investigated its effect on TMP susceptibility compared with susceptibility to other antibiotics. We first made use of a purely genetic way of varying growth. Specifically, we exploited the growth rate variability resulting from genome‐wide gene deletions to expose global trends that are independent of the specific effects of individual gene deletions. Non‐essential gene deletions often reduce the drug‐free growth rate – some by up to ∼50% (Chevereau *et al*, 2015). We re‐analyzed a dataset of growth rates of ∼4,000 *E. coli* gene deletion mutants under different antibiotics representing common modes of action (Chevereau & Bollenbach, 2015); this analysis was genome‐wide and not restricted to a smaller sample of gene deletion mutants, minimizing potential bias. Growth rates were measured at concentrations that inhibit the reference strain by ∼30% to ensure that (i) most gene deletion strains exhibit significantly reduced growth compared to no drug and (ii) most gene deletion strains that are more sensitive to the antibiotic than the wild type still grow exponentially, allowing quantitative analysis. While each gene can have specific effects for each antibiotic (Nichols *et al*, 2011; Chevereau *et al*, 2015), most genes should be unrelated to the drug's mode of action. The global trend of drug susceptibility across all gene deletion strains can thus reveal general consequences of growth inhibition, independent of the specific cellular limitation causing the growth rate reduction.

Non‐specific growth rate changes caused by gene deletions indicate that slower growth protects *E. coli* from TMP but less so from other antibiotics. By correlating the drug‐free growth rate of deletion strains with their growth rate in the presence of drugs, we revealed the dependencies of drug susceptibility on the drug‐free growth rate. The clearest trend emerged for TMP: Its relative effect on growth was weaker in gene deletion strains that had lower growth rates in the absence of drugs (Spearman correlation $\rho_{s}=-0.6$; Fig 1C). Compared to other antibiotics, this effect was most pronounced for TMP (Fig 1D and Appendix Fig S1). Slower‐growing mutants can grow at increased TMP concentrations: While it was technically not feasible to study this genome‐wide, full dose–response curve measurements for a smaller set of 78 arbitrarily selected gene deletion mutants showed that the IC_50_ is weakly negatively correlated with the growth rate in the absence of drug for TMP ( $\rho_{s}=-0.27,\;p=0.019$) but this correlation is not significantly different from zero for other antibiotics (Appendix Fig S2). Thus, TMP represents an extreme case, both in terms of dose‐sensitivity and in terms of susceptibility‐dependence on growth rate. Overall, these results suggest that slower growth generally lowers the susceptibility to TMP.

Slow growth can also protect *E. coli* from other antibiotics but to a far lesser extent. For the prodrug nitrofurantoin (NIT) and the translation inhibitors tetracycline (TET) and chloramphenicol (CHL), there was a weak negative correlation between the drug‐free growth rate and that in the presence of the drug ( $\rho_{s}=-0.31$ for NIT, $\rho_{s}=-0.26$ for TET, $\rho_{s}=-0.22$ for CHL; Fig 1D; Appendix Fig S1). For the beta‐lactam mecillinam (MEC), this trend was even weaker ( $\rho_{s}=-0.14$) and for ciprofloxacin (CPR) almost entirely absent ( $\rho_{s}=-0.05$). There appears to be a tendency for the magnitude of this negative correlation to decrease with increasing dose‐sensitivity when compared among drugs (Fig 1A and D), although this trend is not significant due to the limited number of different drugs and the outliers NIT and CPR deviate from this trend. Although other factors certainly contribute to the shape of dose–response curves, this observation supports the notion that growth‐mediated feedbacks are an important contributor to the shape of the dose–response curve for TMP (Fig 1B) and possibly for other antibiotics as well.

**Figure 2:**
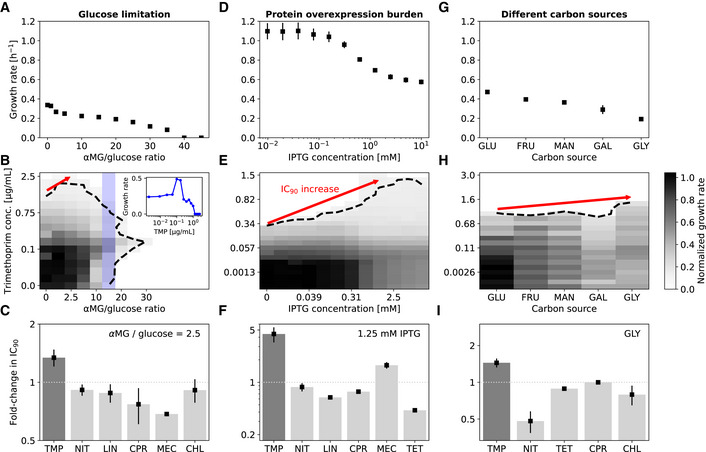
Slower growth generally lowers the efficacy of trimethoprim but not other antibiotics AGrowth rate under glucose limitation achieved by adding the non‐metabolizable structural glucose analog α‐methyl glucoside (αMG) at different ratios to glucose in a minimal medium (Materials and Methods).BNormalized growth rate (gray scale) from a checkerboard assay in a two‐dimensional concentration gradient of TMP and αMG. Dashed black line shows contour line of 90% growth inhibition (IC_90_ line). Red arrow shows increase in IC_90_ as growth is lowered. Inset: Normalized growth rate as a function of TMP concentration along the column marked in blue.CFold‐change in IC_90_ at αMG/glucose ratio 2.5 in assays as in (B) for different antibiotics (Appendix Fig S5). Lowering growth rate increases IC_90_ for TMP but not for other antibiotics.DGrowth rate in rich medium (LB) under different levels of overexpression of a gratuitous protein from a T5‐lac promoter; overexpression burden is controlled by IPTG concentration (Materials and Methods).EAs (B) but for growth rate reduction by protein overexpression in a two‐dimensional concentration gradient of TMP and IPTG.FFold‐change in IC_90_ at 1.25 mM IPTG in assays as in (E) for different antibiotics (Appendix Fig S6). Overexpression of unnecessary protein increases IC_90_ for TMP by almost five‐fold; no comparable increase occurs for other antibiotics.GGrowth rate in minimal medium containing different carbon sources (Materials and Methods): Glucose (GLU), fructose (FRU), mannose (MAN), galactose (GAL), and glycerol (GLY).HNormalized growth rates (gray scale) on different carbon sources (*x*‐axis) at different TMP concentrations (*y*‐axis).IFold‐change in IC_90_ in assays as in (H) for different antibiotics (Appendix Fig S7). Data information: Error bars in (A, D and G) show standard deviation from 6, 12, and 18 biological replicates, respectively; day‐to‐day reproducibility of growth rate measurements is high (Appendix Fig S3). Error bars in (C and F) show standard deviation from three neighboring αMG/glucose ratios and IPTG concentrations centered at 2.5 and 1.25 mM, respectively. IPTG alone has no detectable effect on growth at these concentrations (Appendix Fig S4). Error bars in (I) show standard deviation from three biological replicates. Antibiotic abbreviations are as in Fig 1. CHL was not used in the protein overexpression assay in (F) since the plasmid used for overexpression has a CHL‐resistance marker (Materials and Methods). Sample growth curves are in Appendix Fig S11. The same analysis for the IC_50_ instead of IC_90_ and a different normalization of the dose–response curves is shown in Appendix Fig S15. Source data are available online for this figure.

Reducing growth rate by other means like a nutrient limitation or imposing a protein burden also protects *E. coli* from TMP but less so from other antibiotics. To systematically determine how the efficacy of different antibiotics changes with drug‐free growth rate, we used several independent approaches to change the growth rate. First, we used glucose limitation in batch culture by adding a non‐metabolizable structural analog of glucose, α‐methyl glucoside, in varying concentrations to the growth medium. This analog competes with glucose for uptake into the cell, but unlike glucose it cannot be utilized for growth (Hansen *et al*, 1975). Second, we used different carbon sources (glucose, fructose, mannose, glycerol, and galactose) in the growth medium, which is a classic strategy to test for growth‐dependent effects (Bremer & Dennis, 2008). Third, we overexpressed a gratuitous protein from an inducible promoter to burden the cells (Dong *et al*, 1995; Scott *et al*, 2010). These approaches have different physiological consequences, but they all reduce the growth rate in a gradual and controlled manner, while the maximal growth rate and the accessible dynamic range of relative growth inhibition vary between them (Fig 2). Although TMP can kill bacteria under certain nutrient conditions by causing thymineless death, it can only stop the growth and cause cell stasis in minimal media (Kwon *et al*, 2010). In our experiments, the relevant TMP concentrations are below the MIC and very few cells die, as confirmed by time‐lapse imaging of individual cells in a microfluidic chamber (Appendix Fig S16). This facilitates the interpretation of the data, as the growth rate and the death rate no longer need to be measured separately. Collectively, the three different approaches we used enable us to vary growth rate over a wide range and identify general effects of growth rate, which occur independently of the exact cause of the growth rate reduction.

TMP inhibits growth less under glucose limitation: Lowering the growth rate by glucose limitation enabled bacteria to grow at slightly increased TMP concentrations (Fig 2B and C). This trend was reflected in an increase in IC_90_ and IC_50_, whether these concentrations were defined in terms of the highest drug‐free growth rate (Fig 2) or in terms of the drug‐free growth rate at each level of glucose depletion (Appendix Fig S15). The observed increases were even more pronounced when growth was lowered by overexpressing a gratuitous protein – a truncated and inactive version of *tufB* (Dong *et al*, 1995) expressed from a synthetic promoter P_LlacO‐1_ (Lutz & Bujard, 1997) induced by addition of isopropyl β‐D‐1‐thiogalactopyranoside (IPTG; Fig 2D–F and Appendix Fig S15). Reducing growth by using different carbon sources in a minimal medium could also slightly protect bacteria from TMP, in particular for glycerol (Fig 2G–I and Appendix Fig S15). Changing carbon sources had modest effects, presumably because even the highest growth rate (achieved with glucose only) is relatively low and the fold‐change in growth is considerably smaller than for glucose limitation (Fig 2A, D and G). These effects did not occur to a comparable extent for other antibiotics representing common modes of action (Fig 2C, F and I); however, gratuitous protein overexpression also lowered the susceptibility to mecillinam (MEC), albeit to a lesser extent (Fig 2F and Appendix Fig S6), consistent with the established effect of growth rate on beta‐lactam efficacy (Tuomanen *et al*, 1986; Lee *et al*, 2018). The effects of growth rate changes were clearly drug‐specific and strongest for TMP.

Under severe glucose limitation (high ratios of α‐methyl glucoside over glucose), which does not support growth, the addition of TMP even rescued bacteria and enabled them to grow again (Fig 2B). As a result, the TMP dose–response curve in this regime has a very unusual non‐monotonic shape (inset in Fig 2B) that is hard to interpret in comparison with conventional dose–response curves of Hill‐function shape. Therefore, we restricted further analysis of the effects of drug‐free growth rate on the quantitative shape of the TMP dose–response curve to lower ratios of α‐methyl glucoside over glucose. The non‐monotonic dose–response curve indicates that, under extreme nutrient limitation, the antibiotic TMP can paradoxically promote bacterial growth (inset in Fig 2B) – perhaps the most drastic illustration of the close interplay between drug‐free growth rate and TMP susceptibility we observed.

Lowering growth rate by changing temperature does not show a similar effect: The relative growth reduction by antibiotics remains the same at different temperatures (Appendix Fig S8). This is plausible since, in contrast to other means of altering growth rates, many key physiological parameters are invariant under temperature changes (Bremer & Dennis, 2008). In particular, the concentrations of the most relevant macromolecules in the cell are known to remain constant when the growth rate is altered by a change in temperature in the range from 25°C to 38°C; specific examples include the total amounts of protein, RNA, and RNA polymerase per cell mass, the fractions of total RNA synthesis corresponding to stable RNA and mRNA synthesis, and the DNA replication fork patterns (Bremer & Dennis, 2008). The reason for this behavior is that the corresponding chain elongation rates (for DNA, RNA, and polypeptides) all depend approximately equally on temperature. Therefore, it is plausible to expect that temperature changes have limited effects on the molecular composition and physiology of bacteria. The observation that antibiotic dose–response curves are essentially unaffected by temperature changes indicates that the changes in TMP efficacy observed in response to other means of altering growth rate (Fig 2) have a biological origin that involves changes in the molecular composition of the cell.

**Figure 3:**
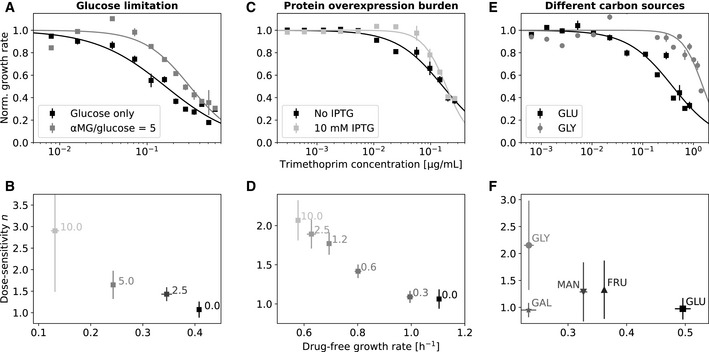
Slower growth increases the steepness of the trimethoprim dose–response curve ATMP dose–response curve in minimal medium with glucose as carbon source and at lower drug‐free growth rate due to glucose limitation, achieved by increasing the αMG/glucose ratio from 0 (black) to 5 (gray). Glucose limitation results in a steeper dose–response curve. Lines show Hill function fits (cf. Fig 1A).BSteepness of TMP dose–response curves (dose‐sensitivity n) versus drug‐free growth rate at different αMG concentrations (Materials and Methods). Numbers next to data points show αMG/glucose ratio.C, DAs (A and B) but for growth limitation by gratuitous protein overexpression in rich growth medium (Materials and Methods). Inducing overexpression with IPTG at 10 mM (light gray) steepens the dose–response curve compared to no induction (black). Numbers next to data points in (D) show IPTG concentration in mM.E, FAs (A and B) but for growth limitation by varying the carbon source in a minimal medium (Materials and Methods). Data information: Carbon sources as in Fig 2. Growth rate error bars show standard deviation of three replicates; vertical error bars in (B,D and F) show standard deviation of parameter estimates from Hill function fit. Non‐normalized dose–response curves are shown in Appendix Fig S14. Source data are available online for this figure.

Slower growth increases the steepness of the TMP dose–response curve. We noticed that the extremely shallow dose–response curve of TMP (Fig 1A) became steeper when growth was slowed by glucose limitation (Fig 3A): Halving the growth rate increased the dose‐sensitivity from n≈1.1±0.2 to n≈1.6±0.3 (Fig 3B). This steepening occurred similarly when growth was slowed by gratuitous protein overexpression (Fig 3C and D) or by changing the carbon source in the growth medium (Fig 3E and F). In addition to this change in steepness, the concentration at which TMP starts to have an effect on growth is higher for slower‐growing bacteria. However, once the effect of TMP kicks in, the growth rate drops more rapidly with increasing TMP concentration. To better understand this unexpected increase in dose‐sensitivity resulting from slower growth, we next aimed to elucidate the underlying mechanism of TMP's growth‐rate‐dependent action.

### Growth‐dependent regulation of the TMP drug target leads to a negative feedback loop that flattens the dose–response curve

Regulation of the drug target DHFR could mediate the growth‐rate‐dependent efficacy of TMP. The abundance of the target of TMP (DHFR/FolA) correlates with growth (Bershtein *et al*, 2013); increasing its expression, e.g. by overexpressing *folA* from a plasmid, alleviates the effect of TMP on growth (Palmer & Kishony, 2014). Accordingly, TMP resistance in the lab and in the clinic often evolves by overexpressing *folA*, e.g. by mutating its promoter or by increasing gene copy number (Rood *et al*, 1980; Flensburg & Sköld, 1987; Toprak *et al*, 2012; Baym *et al*, 2016; Nyerges *et al*, 2018). These phenomena suggest a specific mechanism for the reduced susceptibility to TMP at lower growth rates: We hypothesized that slower growth generally leads to increased *folA* expression, which in turn partially protects bacteria from TMP (Soo *et al*, 2011; Palmer & Kishony, 2014) – a buffering mechanism against inhibition of FolA that is specific to TMP.

**Figure 4:**
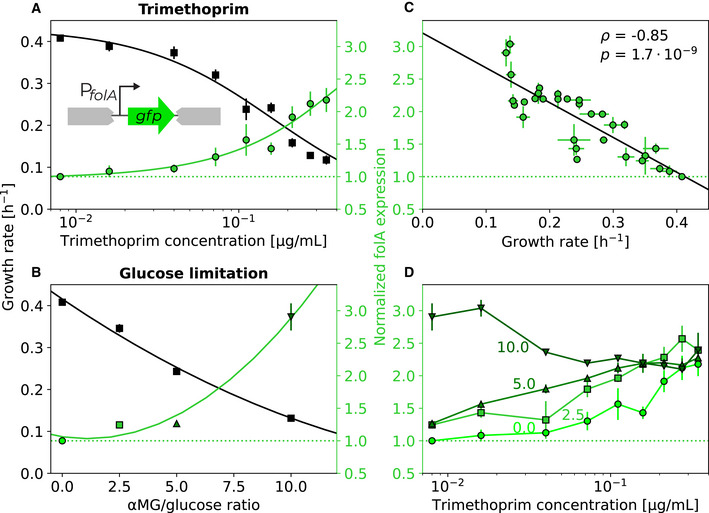
Slower growth increases folA expression, irrespective of whether growth is reduced by trimethoprim or by nutrient limitation ADependence of growth rate (black) and folA expression (green) on TMP concentration. Schematic: FolA expression was measured using a promoter‐GFP reporter inserted at a neutral site in the genome (Materials and Methods).BGrowth rate (black) and folA expression (green) in the absence of TMP at different growth rates achieved by different ratios of αMG/glucose.CScatterplot of folA expression level with growth rate across all combinations of TMP concentrations and αMG/glucose ratios shown in (A and B) (Appendix Fig S9). Pearson's correlation coefficient ρ and *P*‐value from two‐sided *t*‐test are shown. FolA expression is largely determined by growth rate, suggesting that this regulation is growth rate‐dependent and not mediated by a specific molecular mechanism.DDependence of folA expression on TMP concentration at four different αMG/glucose ratios (0, 2.5, 5, and 10 as shown). Darker green indicates greater αMG/glucose ratio. FolA expression converges approximately to the same level at high TMP concentrations. Data information: Black line in (A) shows Hill function fit as in Fig 1A; other lines show polynomial fits of first (C) or second order (A and B) to guide the eye. Horizontal dotted green line shows folA expression level in the absence of TMP. Error bars show standard deviation of three biological replicates. Source data are available online for this figure.

DHFR expression increases similarly in response to TMP and to other means of reducing growth rate, indicating that this regulation is growth rate‐dependent and not mediated by a specific molecular mechanism. Using a folA‐promoter‐GFP reporter (Materials and Methods), we confirmed that *folA* expression increases in response to TMP (Fig 4A), as previously observed in whole populations (Bollenbach *et al*, 2009; Bershtein *et al*, 2015; Rodrigues *et al*, 2016) and single cells (Mitosch *et al*, 2017). Here, however, we noticed that *folA* expression increases similarly when growth is slowed by glucose limitation (Fig 4B). This observation suggests that the upregulation of *folA* under TMP is not a specific response to this drug or the inhibition of its target, but rather a general response to the reduced growth rate. Expression levels of constitutive genes are generally expected to increase when the quality of the nutrient environment is lowered (Scott *et al*, 2010). While the *folA* promoter can be regulated by two transcription factors (TyrR (Yang *et al*, 2007) and IHF (Keseler, 2004)) under certain conditions, it behaved similarly to a constitutive promoter in these experiments. Indeed, *folA* expression across a two‐dimensional concentration gradient of TMP and the glucose analog varied (Appendix Fig S9) but was largely determined by growth rate alone (Fig 4C). Like constitutively expressed genes (Scott *et al*, 2010), *folA* expression followed a general, approximately linear increase with decreasing growth rate, approaching a fixed maximum level at zero growth (Fig 4C). Since increased *folA* expression protects bacteria from TMP (Palmer & Kishony, 2014), this mode of regulation results in a negative growth‐mediated feedback loop: TMP inhibits growth, leading to upregulation of its target, even though there is no specific molecular mechanism for this, thereby attenuating its own efficacy.

Saturating growth‐dependent regulation of the drug target can explain the steepening of the dose–response curve at lower growth rates. Higher drug target expression at lower growth rates can compensate for some of the target inhibition caused by TMP. This offers a plausible explanation as to why the effect of TMP becomes apparent only at higher concentrations when the drug‐free growth rate is lower (Fig 2). But how does slower growth steepen the TMP dose–response curve (Fig 3)? We noticed that *folA* expression at different drug‐free growth rates converges to a fixed value when TMP is added (Fig 4D). In other words, the relative upregulation of *folA* in response to TMP gets weaker with decreasing drug‐free growth rate; it even disappears completely at high glucose‐analog concentrations (Fig 4D). This convergence of *folA* expression in different conditions may reflect that the promoter reaches its maximal induction level. At lower drug‐free growth rates, the promoter is already near its maximum expression level without TMP and saturates quickly when TMP is added, resulting in weaker relative upregulation than at higher growth rates. Consistent with this scenario, increasing *folA* expression at low growth rates is deleterious (Appendix Fig S10). Thus, lower drug‐free growth rates weaken – or even break – the growth‐mediated negative feedback loop, resulting in steeper dose–response curves.

### Artificially breaking the growth‐mediated feedback loop steepens the TMP dose–response curve

**Figure 5:**
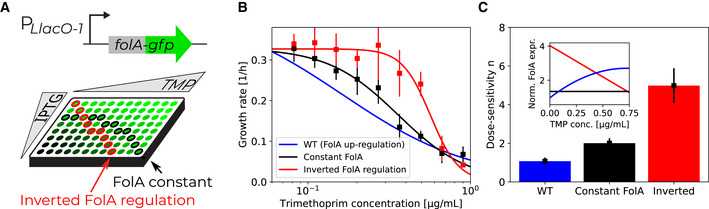
Breaking the growth‐mediated negative feedback loop steepens the trimethoprim dose–response curve ASchematic: FolA expression is controlled by varying the IPTG concentration and measured by flow cytometry using a GFP fusion to FolA. Shades of green indicate different FolA levels. Wells encircled in red indicate how the effect of inverted folA regulation (where folA expression decreases with increasing TMP concentration, starting from a high level) can be inferred; wells encircled in black illustrate the same for constant FolA expression.BGrowth rate as a function of TMP concentration for different paths through IPTG‐TMP concentration space as illustrated in (A) (Materials and Methods). Constant FolA is shown in black and inverted FolA regulation in red. Wild‐type dose–response curve (blue line; fit from Fig 4A) is shown for comparison.CSteepness of the dose response curve (quantified as dose‐sensitivity n) for the three cases in (B). Inset: Normalized FolA expression level as a function of TMP concentration for the three cases in (B); WT (blue) shows fit from Fig 4A; colors as in the bar chart and in (B). Data information: Error bars in (B) show standard deviation of the measured growth rates used for interpolating the values shown; the entire experiment was replicated once (Materials and Methods). Error bars in (C) show standard deviation of parameter estimates from Hill function fit. Source data are available online for this figure.

To corroborate that the shallowness of the TMP dose–response curve is due to a growth‐mediated negative feedback loop, we aimed to break this loop even under nutrient conditions that support high drug‐free growth rates. To this end, we constructed a synthetic strain in which the expression of *folA* from its endogenous locus is controlled by an inducible promoter P_*Llac‐O1*_. The strain allowed IPTG‐mediated induction of *folA* and *folA‐gfp*, respectively, with an expression level comparable to wild‐type *folA* at low induction (Materials and Methods). Note that using an inducible promoter alone does not eliminate the feedback loop since, at constant inducer levels, expression from this promoter can change with growth rate, similar to expression from the endogenous *folA* promoter. Nevertheless, we can use this synthetic strain to infer the shape of the TMP dose–response curve at constant *folA* expression by continuously varying the inducer concentration and measuring FolA levels. Specifically, we measured growth rate and *folA* expression using a FolA‐GFP fusion protein across a two‐dimensional concentration gradient of TMP and inducer (Fig 5A; Materials and Methods). We then determined the growth rate as a function of TMP concentration on a path through this two‐dimensional concentration space along which *folA* expression is constant. The resulting TMP dose–response curve at constant *folA* expression is steeper than in wild type ( n=2.0±0.3; Fig 5B and C). It becomes even steeper for a positive feedback loop, which is inferred from a path through the two‐dimensional concentration space along which *folA* expression is inverted compared to wild type, i.e., starting from a high level, it decreases with increasing TMP concentration ( n=5.0±0.9; Fig 5B and C). Placing *folA* under an inducible promoter results in a slightly lower drug‐free growth rate compared to wild type (by about 20%); however, this effect alone cannot explain the observed increases in dose‐sensitivity with constant and inverted *folA* regulation, because a reduction in the growth rate of this magnitude increases dose‐sensitivity to at most n≈1.5 for several different ways of changing growth rate (Fig 3B, D and F). These results provide direct evidence that a negative growth‐mediated feedback loop implemented by the regulation of the drug target causes the exceptional shallowness of the TMP dose–response curve.

### A cellular resource allocation model captures the effect of trimethoprim on bacterial growth

To determine whether the metabolic limitation caused by TMP due to inhibition of its target FolA, together with the growth‐rate‐dependent regulation of *folA*, is sufficient to explain the experimentally observed phenomena related to the shape of the TMP dose–response curve, we developed a mechanistic mathematical model of cellular resource allocation under this drug. The model is based on Constrained Allocation Flux Balance Analysis (CAFBA; Mori *et al*, 2016) and captures how the interaction of TMP with its target enzyme FolA reduces the metabolic flux through the folate synthesis pathway, thereby reducing the growth rate (Materials and Methods). The key assumptions of the model are that the demand for FolA is constant and that growth is limited by the metabolic flux catalyzed by FolA when sufficiently many FolA enzymes are blocked by TMP. These assumptions reflect the basic intuition that the growth inhibition at sufficiently high TMP concentrations is due to a bottleneck in the folate synthesis pathway caused by this drug, whereas growth at low TMP concentrations is limited by ribosomes, as dictated by bacterial growth laws. The transition between these two regimes occurs at an intermediate TMP concentration, the value of which increases with FolA content in the cell, since more copies of FolA must be blocked by TMP to achieve the same reduction in metabolic flux. The model has four parameters. Two of these describe growth in the drug‐free regime; these were fitted to the data at negligible TMP concentration (*c* < 0.1 μg/ml). We subsequently fitted the remaining two parameters, which specifically relate to the drug response, to the remaining data; they quantify the cellular demand for FolA and the equilibrium constant for the binding between FolA and TMP. We used the measured regulation of *folA* expression in response to TMP and glucose limitation (Fig 4) as an input to this model and calculated the TMP dose–response curve at different levels of glucose limitation in wild type.

**Figure 6:**
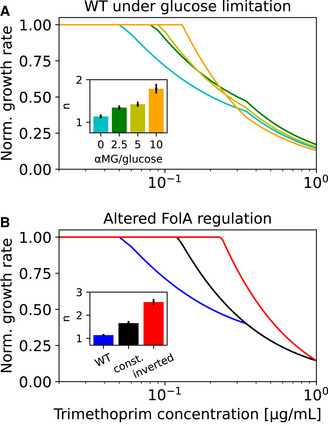
Mathematical model of bacterial resource allocation supports that the trimethoprim dose–response curve is shaped by folA regulation ADose–response curves calculated from mathematical model in wild type at different levels of glucose limitation (αMG/glucose ratios) shown by different colors. Inset: dose‐sensitivity n of these dose–response curves. The steepness of the dose–response curve increases with glucose limitation as observed experimentally (cf. Fig 3A and B).BDose–response curves calculated from mathematical model for wild‐type folA regulation (blue), constant intracellular FolA level (black), and inverted folA regulation (red). Inset: dose‐sensitivity n of these dose–response curves. Dose–response curve steepens as observed experimentally for these perturbations (cf. Fig 5B and C).

Using plausible parameter values (Materials and Methods), the model produces a TMP dose–response curve with a similar steepness ( n≈1.1) as observed experimentally (Fig 6A). Glucose limitation leads to a steepening of the dose–response curve (Fig 6A), similar to that observed experimentally (Fig 3A and B). Further, moderate glucose limitation can slightly increase the absolute growth rate under TMP (Appendix Fig S12) as observed experimentally (Fig 2B). In essence, these effects can be intuitively understood: they are due to the upregulation of *folA* under glucose limitation, resulting in an excess of FolA in the cell. This excess in turn buffers the effect of TMP: higher TMP concentrations are required for the growth rate to decrease as more drug targets become available at reduced growth, leading to a shift in the TMP concentration at which a growth inhibitory effect begins to occur to higher values in Fig 6A. Because the upregulation of *folA* in response to TMP also becomes weaker under increasing glucose limitation (Fig 4D), the decrease in growth rate as a function of TMP concentration becomes steeper (Fig 6A) as the negative feedback loop that dampens the effect of the drug is weakened.

Mimicking synthetic perturbations of this feedback loop in the model further supports the notion that upregulation of the drug target with decreasing growth rate is the key mechanism underlying the shallow dose–response curve of TMP. Replacing the wild‐type regulation of *folA* expression in the model with alternative modes of regulation in which it is forced to a constant value or inverted (i.e., downregulated from a high value with increasing TMP concentration) results in a successive steepening of the dose–response curve (Fig 6B; Materials and Methods) that is quantitatively similar to that observed experimentally for these perturbations (Fig 5B and C). Taken together, the results of the model support that the TMP dose–response curve is shaped by a transition from ribosome‐limited to FolA‐limited growth and by the strength of the negative feedback loop, which is mediated by the regulation of the drug target FolA.

## Discussion

We showed that slower‐growing bacteria are generally less affected by TMP, largely regardless of what causes their slower growth (Fig 2). This phenomenon, which did not occur for most other antibiotics tested here, implies a growth‐mediated negative feedback loop causing TMP's extremely shallow dose–response curve (Fig 1A): TMP lowers growth, which in turn weakens the inhibitory effect of the drug. Mechanistically, this feedback loop is rooted in the expression level of the drug target DHFR, which is upregulated with decreasing growth rate (Fig 4). Elimination or inversion of this feedback loop from negative to positive drastically steepens the dose–response curve (Fig 5). Together with recent work on ribosome‐targeting antibiotics (Deris *et al*, 2013), these results which are specific to TMP suggest a more general role of growth‐mediated feedbacks in shaping antibiotic dose–response curves (Fig 1B).

Consistent with this view, the steepness of the dose–response curve of antibiotics with different modes of action often changes in tandem with the change in drug susceptibility under slower growth (Fig 1A and D). In particular, while the effect is less extreme than for TMP, the ribosome inhibitors CHL and TET also exhibit relatively low dose‐sensitivity and slightly reduced susceptibility under slower growth (Fig 1D and Appendix Fig S1). The mechanism underlying this weaker growth‐mediated negative feedback for CHL and TET, while certainly not mediated by the regulation of *folA* as for TMP, is conceptually similar to that for TMP, in that their drug target, the ribosome, is upregulated in response to these drugs (Scott *et al*, 2010)—similar to DHFR in response to TMP. In contrast to TMP, this upregulation of ribosome production is a specific response to growth inhibition by CHL or TET since lowering the growth rate by nutrient limitation results in the opposite behavior, downregulation of ribosome production (Scott *et al*, 2010). Other factors can certainly also play a role in shaping dose–response curves. For example, the prodrug NIT does not follow the trend: It has a relatively steep dose–response curve (Fig 1A) despite being less susceptible under slower growth (Fig 1D). This is probably caused by additional (unknown) mechanisms acting on top of the growth‐mediated feedback we focus on here. Overall, although there are indications for a more general role of growth‐mediated feedback loops in antibiotic responses, identification of the molecular mechanisms underlying these feedback loops or other phenomena that shape dose–response curves will require detailed studies for each antibiotic or antibiotic class.

Notably, a growth‐mediated negative feedback loop could lead to limit cycle oscillations in growth rate if there is a sufficient time delay between the onset of the effect of TMP and the resulting change in growth rate. If they exist, such oscillations could in principle be observed for the instantaneous growth rate at the single‐cell level: This growth rate should decrease when TMP is added, leading to slower growth, which in turn decreases the efficacy of TMP a little later, leading to faster growth, which in turn increases the efficacy of TMP, and so on. We could not detect such oscillations in single‐cell time‐lapse experiments (Appendix Fig S16); if they exist, their detection may require more precise measurements of the instantaneous growth rate of individual bacteria (Godin *et al*, 2010).

We used a mathematical model of resource allocation in the bacterial cell to explain the growth rate based on nutrient quality, drug concentration and *folA* expression. After a suitable rescaling of these variables, the model comprises only a single meaningful parameter (Materials and Methods), which highlights its simplicity. The model supports that the regulation of TMP's target DHFR underlies the growth‐mediated feedback for this drug. We assumed that DHFR is upregulated with decreasing growth rate and approaches a maximum at zero growth as experimentally observed (Fig 4C). Decoupling the DHFR level from the growth rate by forcing it to a constant value, or even inverting its response to TMP, results in a steeper dose–response curve (Fig 6B) in agreement with experimental observations (Fig 5B and C). The mathematical model thus confirms the intuitive expectation that the negative feedback loop mediated by the regulation of the drug target is the main cause of the shallow TMP dose–response curve. It further helps to rationalize why dose–response curves become steeper when the drug‐free growth rate is decreased, corresponding to a poorer nutrient environment. At very low drug‐free growth rates, the DHFR level becomes almost constant as a function of TMP concentration (Fig 4D), effectively breaking the negative feedback loop and thus steepening the dose–response curve, as observed experimentally (Fig 3). However, we note that the resource allocation model does not reproduce the unusual, non‐monotonic TMP dose–response curve observed experimentally under severe glucose limitation (inset in Fig 2B), suggesting that this extreme limitation may be too far removed from the physiological conditions assumed in the model. Explaining the shape of dose–response curves of other antibiotics using suitable mathematical models remains a challenge for future work.

We observed that an artificial nutrient limitation that results in no or extremely slow growth can be alleviated by adding the antibiotic TMP (Fig 2B). This phenomenon is qualitatively consistent with the TMP‐specific mechanism we identified: *folA* expression is greatly increased at low drug‐free growth rates (Fig 4C), leading to a situation in which blocking a fraction of FolA in the cell by TMP promotes growth, probably by rebalancing metabolic resources in the cell. This indicates that bacteria may not regulate DHFR expression in a way that maximizes growth under extreme nutrient limitation. High *folA* expression as occurs during slow growth (Fig 4C) is deleterious (Bhattacharyya *et al*, 2016) because cellular resources are diverted toward excessive folic acid synthesis. Consistent with this view, TMP facilitated bacterial growth when *folA* was artificially overexpressed to levels that were deleterious in the absence of TMP (Appendix Fig S10). Together, these observations show that DHFR level is the main driver of TMP susceptibility and suggest that deleterious overproduction of DHFR, which can be rescued by adding TMP, occurs under extreme nutrient limitation. Since TMP increases the fitness of bacteria that evolve under extreme nutrient limitation, the usual selection pressure for antibiotic resistance is inverted under such conditions: Mutations that usually enhance TMP action (e.g. increased drug uptake) can be selected. Similar to certain drug combinations (Chait *et al*, 2007), this situation provides an opportunity to select against antibiotic‐resistant bacteria. One potential advantage of creating such conditions with a sugar analog instead of a second drug is that bacteria can hardly evolve resistance to such an analog, as impaired sugar uptake would come at a massive fitness cost.

## Materials and Methods

### Reagents and Tools table

**Table:** 

Reagent/Resource	Reference or Source	Identifier or Catalog Number
**Experimental Models**		
*E. coli* BW25113	Baba *et al* (2006)	N/A
*E. coli* BWAA01	This study	N/A
*E. coli* BWAA02	This study	N/A
*E. coli* BWAA11	This study	N/A
*E. coli* BWAA12	This study	N/A
*E. coli* BWAA19	This study	N/A
*E. coli* BWAA20	This study	N/A
*E. coli* BW25141	Datsenko & Wanner (2000)	CGSC#: 7633
*E. coli* HG105	Garcia *et al* (2011)	N/A
**Recombinant DNA**		
pSIM19	Datta *et al* (2006)	N/A
pCP20	Cherepanov & Wackernagel (1995)	N/A
pKD13‐mutgfp	Guet lab (IST Austria)	Bor Kavcic
pCA24N(‐)tufB	Kitagawa *et al* (2005)	N/A
pCA24N(‐)folA	Kitagawa *et al* (2005)	N/A
pCA24N(‐)	Kitagawa *et al* (2005)	N/A
pAAtufB	This study	N/A
pAA30	This study	N/A
pAA39	This study	N/A
pAA40	This study	N/A
pCS‐λ	Kishony & Leibler (2003)	N/A
pZS11‐pHluorin	Mitosch *et al* (2017)	N/A
**Oligonucleotides and sequence‐based reagents**		
PCR primers	This study	Table EV1
**Chemicals, enzymes and other reagents**		
SmaI restriction enzyme	New England Biolabs	Cat # R0141S
LB Broth Lennox	Sigma Aldrich	Cat # L3022
Na_2_HPO_4_.7H_2_O	Fisher Scientific Acros Organics	Cat # 206515000
KH_2_PO_4_	Sigma Aldrich	Cat # P9791
NaCl	Sigma Aldrich	Cat # S3014
NH_4_Cl	Sigma Aldrich	Cat # A9434
CaCl_2_	Fluka	Cat # 223506
MgSO_4_	Sigma Aldrich	Cat # M7506
Triton‐X 100	Sigma Aldrich	Cat # T8787
Glucose	Sigma Aldrich	Cat # G8270
Glycerol	VWR	Cat # 854
Mannose	Carl Roth	Cat # 4220.2
Fructose	Sigma Aldrich	Cat # F0127
Galactose	Sigma Aldrich	Cat # G0750
LB agar	Sigma Aldrich	Cat # L2897
Chloramphenicol	Sigma Aldrich	Cat # C0378
Kanamycin	Sigma Aldrich	Cat # K4000
Ampicillin	Sigma Aldrich	Cat # A9518
Spectinomycin	Sigma Aldrich	Cat # S6501
Ethanol	Sigma Aldrich	Cat # 32221
Trimethoprim	Sigma Aldrich	Cat # 92131
Nitrofurantoin	Sigma Aldrich	Cat # N7878
Lincomycin	Sigma Aldrich	Cat # 62143
Mecillinam	Sigma Aldrich	Cat # 33447
Tetracycline	Sigma Aldrich	Cat # 268054
Ciprofloxacin	Sigma Aldrich	Cat # 17850
IPTG	VWR	Cat # 437144N
α‐methyl glucoside	Sigma Aldrich	Cat # M9376
ISOTON II	Beckman Coulter	N/A
**Software**		
Matlab R2016b	MathWorks Inc.	N/A
Cytexpert 2.3.0.84	Beckman Coulter	N/A
**Other**		
Pin tool VP407	V&P Scientific Inc., CA, USA	N/A
Pin tool VP408	V&P Scientific Inc., CA, USA	N/A
Shaking incubator Innova 44	Eppendorf New Brunswick, DE	N/A
Plate reader Synergy Neo2	Biotek Inc., VT, USA	N/A
Plate reader Synergy H1	Biotek Inc., VT, USA	N/A
Plate reader Infinite M1000 Pro	Tecan Inc., CH	N/A
Transparent microtiter plates FT 96‐well	Nunc Thermo Scientific	Cat # 236105
White microtiter plates FW 96‐well	Nunc Thermo Scientific	Cat # 260860
Transparent foil TopSeal‐A Plus	PerkinElmer	N/A
CytoFLEX B2‐RQ‐V2 with 96‐well plate module	Beckman Coulter	N/A
CellASIC ONIX microfluidic device	Merck Millipore	N/A

### Methods and Protocols

#### Growth conditions and growth rate measurements

The growth medium used was either LB Broth Lennox, pH set to 7.0 with NaOH before autoclaving, or M9 minimal medium made from Na_2_HPO_4_.7H_2_O, KH_2_PO_4_, NaCl, and NH_4_Cl supplemented with 0.1 mM CaCl_2_, 2 mM MgSO_4_, and 0.001% (v/v) Triton‐X 100. Triton‐X was added to flatten the meniscus that forms in 96‐well plates (Mitosch *et al*, 2017). Carbon sources in the M9 medium were glucose, glycerol, mannose, fructose, and galactose, all of which were added at 0.4% (w/v) and prepared as filter sterilized 20% (w/v) stock solutions stored at room temperature in the dark. Experiments were started from a frozen glycerol stock. Bacteria were streaked on an LB agar plate (containing antibiotics as appropriate) incubated overnight at 37°C and a single colony was inoculated in 2 ml of the appropriate growth medium (containing antibiotics if appropriate) and grown for about 20 h to obtain a pre‐culture that has reached stationary phase. We inoculated experimental cultures with a 1,000‐fold dilution from a stationary phase culture when growth was determined by optical density measurements at 600 nm (OD_600_). For the experiment in which temperature was varied (Appendix Fig S8), a luminescence readout was used; here, the pre‐culture was grown in a 20 ml LB medium in a 250 ml flask until the stationary phase; 100 μl aliquots were transferred to the wells of a 96‐well plate, supplemented with glycerol to 15% and frozen at −80°C. To start a luminescence‐based experiment the plate was thawed, and dilutions were performed in 96‐well plates with fresh medium using pin tools (VP407 and VP408, V&P Scientific Inc., CA, USA), which transfer 1.5 and 0.2 μl per well, respectively. Subsequent use resulted in a 10^7^‐fold dilution from a stationary phase culture. In all cases, the pre‐cultures were incubated at 30°C with a shaking speed of 250 rpm (Innova 44, Eppendorf New Brunswick, DE).

Pre‐cultures carrying plasmids and cultures needed for molecular cloning procedures were prepared with antibiotics at the following concentrations: chloramphenicol 35 μg/ml, kanamycin 25 μg/ml, ampicillin 50 μg/ml, spectinomycin 100 μg/ml.

Unless otherwise noted antibiotics were dissolved in ethanol. Stock solutions in water were filter‐sterilized. Aliquots of stocks were stored at −20°C in the dark. The antibiotics used were trimethoprim, nitrofurantoin, chloramphenicol, lincomycin (dissolved in water), mecillinam (dissolved in water), tetracycline, and ciprofloxacin (dissolved in water). IPTG was added to cultures to control expression from IPTG‐responsive promoters (P_T5‐lac_, P_LlacO‐1_) (Lutz & Bujard, 1997; Kitagawa *et al*, 2005). A filter‐sterilized solution of 1 M IPTG in water served as a stock solution. IPTG was stored at −20°C in the dark and aliquots were thawed at room temperature before use. For the non‐metabolizable glucose analog α‐methyl glucoside, which competes for glucose uptake and essentially imposes glucose limitation (Hansen *et al*, 1975), a filter sterilized solution of 50% (w/v) in M9 salts served as the stock solution.

The experiments shown in Figs 1, 2, 3 were performed using a robotic system as described previously (Chevereau *et al*, 2015) and have a day‐to‐day variability (coefficient of variation, CV) of growth rate for unperturbed cultures of less than 5% (Ref. Chevereau *et al*, 2015 and Appendix Fig S3). The experiments shown in Figs 4 and 5 were performed using two plate readers: A Synergy Neo2 and a Synergy H1 (both from Biotek Inc., VT, USA). Both were set to 30°C with continuous shaking at an orbital displacement of 1 mm and a speed of 807 rpm, and after a settling period of 10 s the optical density at 600 nm and GFP fluorescence were measured every 10 min. Flat transparent microtiter plates (Nunc Thermo Scientific FT 96‐well) with lids were used. The experiments presented in Appendix Fig S8 were performed using luminescence measurements in an Infinite M1000 Pro plate reader (Tecan Inc., CH) equipped with an integrated stacking module. The stack was housed in a custom‐built (IST Austria Miba Machine Shop, Klosterneuburg, AT) acrylic glass box equipped with a custom‐built heating block, a thermostat, and strong ventilation to assure a homogenous temperature over the plates and the stack (Kavčič *et al*, 2020). For these experiments (Appendix Fig S8), the wild‐type strain used here (*E. coli* BW25113) was transformed with a kanamycin resistance‐bearing plasmid (pCS‐λ) carrying luciferase genes used to determine the growth rate (Kishony & Leibler, 2003; Chait *et al*, 2007). For the actual growth experiments kanamycin was omitted; however, this was not a problem as the plasmid is retained throughout the duration of such an experiment (Kavčič *et al*, 2020). We have recently also verified that the luminescence setup used here results in the same growth rates as obtained from OD measurements (Kavčič *et al*, 2020). Luminescence assays were performed using flat white microtiter plates (Nunc Thermo Scientific FW 96‐well). These plates were sealed with a transparent foil (TopSeal‐A Plus, PerkinElmer) and about 10 plates were used per stack. Luminescence was measured every 10–20 min. Before each measurement, plates were shaken for 10 s at 582 rpm with a 1 mm amplitude. The culture volume per well was 150 μl. The day‐to‐day CV for unperturbed cultures for the growth rate in the luminescence‐setup was 3%.

The growth rate was determined by a linear fit of the log‐transformed and background‐subtracted OD_600_ from the exponential growth phase of the cultures using custom Matlab (R2016b, MathWorks Inc.) scripts. To capture the exponential growth phase for cultures in LB we used background‐subtracted OD_600_ windows of 0.02 to 0.2 and for minimal medium 0.03 to 0.12; these windows cover one order of magnitude and at least two doublings and take the lower growth yield in the minimal medium into account. The lowest accepted growth rate for LB was 0.1 h^−1^ and for minimal medium 0.03 h^−1^, both corresponding to about 10% of the respective unperturbed maximal growth rate. For the Hill function fits in Fig 3C and D, growth rates below 0.2 h^−1^ were ignored because too many data points fell in this range at higher IPTG concentrations (Fig 2E) – these data points would thus dominate the fit, which is undesirable since they contain less information about the shape of the dose–response curve (i.e. the dose‐sensitivity). The duration of experiments for LB cultures was about 22 h, for minimal medium about 46 h. For all experiments performed in LB medium, data after ∼1,000 min were discarded to avoid the inclusion of faster growing mutants which occurred sporadically in the presence of antibiotics; this was not necessary for experiments in minimal medium. To capture the growth rate strictly during the exponential phase from the luminescence‐based experiments, the rate of luminescence increase was determined by a linear fit of the log‐transformed data between 10^2^ cps and 10^5^ cps.

#### Expression level measurements using plate readers

Two plate readers Synergy Neo2 and a Synergy H1 (see section *Growth conditions* for further details) were used for GFP fluorescence measurements. The filter set used in the Neo2 provided excitation at 485 nm (BW20) and emission at 516 nm (BW20; Biotek fluorescent filter #105). The settings for the monochromator‐based H1 model were 485 nm for excitation and 528 nm for emission. Both readers produced consistent values and results. The measured values for experiments where both plate readers were used in parallel were adjusted accordingly (i.e. simply normalized by a constant obtained from measuring the same sample on both readers). The expression level was determined essentially as described (Zaslaver *et al*, 2006; Mitosch *et al*, 2017). Briefly, for each GFP‐expressing strain, a similar strain without GFP‐expression was grown in parallel in the same conditions (see section *Strain construction* for further details). For both strains, the exponential growth phase was determined and the background subtracted GFP‐signal from the GFP‐less strain was subtracted from the GFP‐carrying strain for cultures with similar growth rates and at the same OD_600_. As the exact same OD_600_ values were mostly not met, linear interpolation (Matlab function *interp1*) was used to generate an interpolated GFP‐value between the two GFP values of the two nearest OD_600_ values. The expression level is obtained from the slope of a linear fit (Matlab function *fit*) to the GFP over OD_600_ data during exponential growth. In the experiments using the strains with the reporter construct with the native promoter (BWAA01, Fig 4), fast‐folding GFP (Zaslaver *et al*, 2006) was used whereas in the experiments with the synthetic IPTG‐inducible promoter construct (BWAA19, Fig 5) the GFP from the ASKA‐library (Kitagawa *et al*, 2005) was used.

#### Expression level measurements using flow cytometry

For the expression level determination of the strains with IPTG‐induced *folA‐gfp* expression (Fig 5) we used a combination of plate readers (Biotek Synergy H1) for optical density measurements for growth rate determination (see section Growth rate measurements for details) and flow cytometry (Beckman Coulter CytoFLEX B2‐RQ‐V2 with 96‐well plate module) for fluorescence measurements. Flow cytometry was used because of its higher signal‐to‐noise ratio compared to fluorescence measurements on plate readers. Strains were grown in the plate readers and growth was monitored by measuring optical density every 10 min. When strains were in mid‐exponential growth phase (OD ∼ 0.1), they were diluted 1,000‐fold in ISOTON II (Beckman Coulter) and measured immediately on the flow cytometer. Gating in SSC‐A and GFP FITC‐A channels in the flow cytometry analysis software (Beckman Coulter Cytexpert 2.3.0.84) allows the finding of (fluorescent) cells and the determination of the mean and relative coefficient of variation of fluorescence intensity. Strains used were BWAA11, BWAA12, BWAA19, and BWAA20 (see section *Strain construction* for details) and TMP and IPTG gradients starting at 0.9 μg/ml and 2.5 mM were applied, respectively. Growth rates at constant or decreasing *folA* expression levels were calculated by linear interpolation of the growth rates measured at different IPTG and TMP concentrations as illustrated in Fig 5A. This experiment involves two‐dimensional concentration gradients on the 96‐well plate and the parallel use of three plate readers (each measuring one plate over time) and requires repetitive sampling of the 96‐well plates; this is unusually laborious and time‐consuming and was therefore replicated only once.

#### Strains and strain construction

We used *E. coli* BW25113 and several derivatives thereof. BW25113 is the parent strain of the KEIO collection, a widely used whole‐genome deletion mutant collection (Baba *et al*, 2006). For the overexpression experiments, BW25113 was transformed with the necessary plasmids (Reagents and Tools table) which stem from the ASKA‐library, a plasmid‐based whole‐genome overexpression collection (Kitagawa *et al*, 2005). To reduce the growth rate by gratuitous protein expression we used a truncated elongation factor Tu (EF‐Tu, *tufB*) as previously done for a similar purpose (Dong *et al*, 1995). Briefly, starting with the ASKA‐library plasmid carrying *tufB* (pCA24N(−)tufB), the SmaI restriction fragment of 243 bp in length was cut out and the blunt‐ended DNA fragment was closed by ligation to form a plasmid again, named pAAtufB here. This deletion results in a shortened, non‐functional gene (*ΔtufB*), which can be used to provide gratuitous protein expression, resulting in a burden that slows down growth (Dong *et al*, 1995; Scott *et al*, 2010). The plasmids from the ASKA‐library (Kitagawa *et al*, 2005) use the P_T5‐lac_ promoter, which allows for a graded control of expression by the addition of the inducer IPTG (which works sufficiently well in a *lac*‐operon compromised strain like *E. coli* BW25113). As a control, we used pAA30 which is the empty ASKA plasmid modified to not contain a gene to prevent any expression; we created this plasmid since the original empty ASKA plasmid, pCA24N(−), does in fact encode a short coding sequence in frame with the promoter. Briefly, through a PCR with the overlapping primers #1 and # 2 (Table EV1; for general strategy see Heckman & Pease, 2007; Hansson *et al*, 2008) a short stretch of pCA24N(−) encompassing start codon over the His‐Tag and until the stop codon, was eliminated. The elimination was confirmed by sequencing the resulting plasmid with the primers #3 and #4 (Table EV1) flanking the gene insertion site. For the strong *folA* overexpression, the ASKA plasmid pCA24N(−)folA was used.

We generated reporter strains and a strain with inducible *folA* regulation. To construct the first *gfp*‐reporter and corresponding *gfp*‐less control pair integrated into the chromosome (BWAA01 and BWAA02), the promoter‐reporter construct for P_folA_ and the corresponding region from the empty plasmid pUA66 from the reporter library (Zaslaver *et al*, 2006) were integrated into a neutral site (*phoA*) in the genome, respectively. To this end, P1 transduction was used to move the construct from an MG1655 strain carrying the reporter constructs (Bollenbach *et al*, 2009) into the BW25113 background. The insertion was confirmed by sequencing PCR products generated using the primers #5 and 6 (Table EV1) binding outside the *phoA* locus.

The other reporters were based on *folA‐gfp* fusion constructs from the ASKA‐library (Kitagawa *et al*, 2005). Again, pairs of strains were made where each pair consists of a strain with and a strain without the *gfp* fused to *folA*. We generated one pair to induce and thereby control expression level by an IPTG‐responsive promoter (P_LlacO‐1_; Lutz & Bujard, 1997) and one pair with the native regulation through P_folA_. The latter strains (BWAA11 native regulation folA:gfp:kan and BWAA12 native regulation folA:kan) were made to validate that the induced expression matches the expression level of the native regulation. Indeed, the inducible strains (BWAA19 IPTG‐inducible regulation ΔPfolA:kan:PLlacO‐1:folA:gfp, intS:PLlacO‐1:lacI, ΔlacI:kan and BWAA20 IPTG‐inducible regulation ΔPfolA:kan:PLlacO‐1:folA, intS:PLlacO‐1:lacI, ΔlacI:kan) show similar expression level as WT‐regulation when lowly induced (0.5 mM IPTG) and slightly less than 5‐fold induction when strongly induced (2.0 mM IPTG). To create the *gfp* fusion strains, *folA‐chlR* and *folA‐gfp‐chlR* fragments were PCR‐amplified from the *folA*‐carrying ASKA‐library plasmids (using as template the respective plasmids from the library, with and without *gfp* (Kitagawa *et al*, 2005)) as a first step and were used for recombineering (Datsenko & Wanner, 2000) into the plasmid pKD13‐gfpmut3 (a derivative of pKD13 (Datsenko & Wanner, 2000); gift from Bor Kavčič which contains FRT‐flanked kanamycin resitance cassette and the promoter sequence PLlacO1). Primers used for that step were #7 and #8 (Table EV1). This resulted in pAA39 and pAA40 where an FRT‐flanked kanamycin resistance cassette, the promoter P_*LlacO‐1*_ driving *folA* and the *folA‐gfp* fusion, respectively, and a chloramphenicol resistance cassette are present (in this order). These plasmids first served as the source for the promoter‐*folA* and *folA‐gfp* fusion with a chloramphenicol resistance cassette to be inserted into the genome at the *folA* locus to generate the strains with the native regulation (BWAA11 and BWAA12). PCR‐fragments for recombineering were obtained with the previously used forward primer #7 and primer #9 (Table EV1) serving as a reverse primer to get the *folA* gene with and without *gfp*, respectively, and the chloramphenicol resistance cassette (but not the synthetic promoter) were inserted into the genome of BW25113 replacing the *folA* gene (but not the promoter on the genome). Next, by recombineering with PCR‐fragments containing the kanamycin resistance cassette only obtained with the primer #10 and the primer #11 (Table EV1) from pKD13‐gfpmut3 the chloramphenicol resistance cassette was replaced with the FRT‐flanked kanamycin resistance cassette. Next, to obtain a marker‐less strain the kanamycin resistance cassette was removed using the plasmid pCP20, the FLP helper plasmid, as described (Cherepanov & Wackernagel, 1995). For the strains with the IPTG‐inducible regulation (BWAA19 and BWAA20) a similar strategy was applied. Primer #12 and primer #13 (Table EV1; putative RBS sequence from Baba *et al*, 2006) were used to amplify the FRT‐flanked kanamycin resistance cassette, the promoter P_*LlacO‐1*_ driving *folA* and the *folA‐gfp* respectively of pAA39 and pAA40 (but not the chloramphenicol resistance cassette). Next, to obtain a marker‐less strain the kanamycin resistance cassette was removed using pCP20. All four marker‐less strains were further modified by P1 transduction from a MG1655 strain carrying the *lacI* gene under the promoter P_*lacO1*_ and a FRT‐flanked kanamycin resistance cassette at the neutral insertion site *intS* (based on the strain from (Garcia *et al*, 2011) (HG105) and a gift from Bor Kavčič). The insertion was confirmed by sequencing PCR products generated using primers #14 and #15 (Table EV1) binding outside the *intS* locus. Next, that kanamycin resistance cassette was removed using pCP20. The resulting markerless strains were further modified by P1 transduction with the *lacI* knock‐out strain from the KEIO collection (Baba *et al*, 2006) replacing the *lacI* gene with the kanamycin resistance cassette. The deletion was confirmed by sequencing PCR products generated using primers #16 and #17 (Table EV1) binding outside the *lacI* locus. We reasoned, that here a P_L*lacO‐1*_‐driven *lacI* allows a better control of the P_L*lacO‐1*_‐driven *folA* based on observations in Klumpp *et al* (2009) and Kavčič *et al* (2020) dealing with growth rate independent negative autoregulation. Moreover, as mentioned above, with the combination of the respective RBS and the Lac‐repressor driven by P_*lacO1*_ we achieved expression relatively close to wild‐type levels (Liu & Naismith, 2008; Deris *et al*, 2013).

For the recombineering procedure (Datsenko & Wanner, 2000) the temperature‐inducible system from pSIM19, the recombineering helper plasmid, (Sharan *et al*, 2009) was used. Chloramphenicol at 10 μg/ml and kanamycin at 25 μg/ml were used. During the whole strain construction procedure wherever *folA* was driven by PL_*lacO1*_, 1 mM IPTG was added as this inducer controls expression from PL_*lacO1*_ and *folA* is an essential gene.

#### Microfluidics‐based single‐cell experiments to determine cell death in the presence of TMP

The time‐lapse microscopy experiments were performed as previously described (Mitosch *et al*, 2017, 2019). Briefly, we used *E. coli* BW25113 carrying the low copy plasmid pZS11‐*pHluorin* (Mitosch *et al*, 2017) with *pHluorin* from (Martinez *et al*, 2012) and PLtetO‐1 promoter with absent Tet repressor (Lutz & Bujard, 1997), leading to the constitutive expression of *pHluorin*; here, *pHluorin* is only used as a cytosolic fluorescent protein for segmentation, i.e., to follow single cells and determine growth rate, and to detect cell lysis. We used a microfluidic device (CellASIC ONIX, Merck Millipore) in which bacteria grow in microcolonies. It allows inflow from different inlets; the growth medium in the microfluidic chamber can be completely exchanged within minutes. Bacteria were inoculated from frozen glycerol stocks, grown to exponential growth phase, diluted, and then added into the preheated (30°C) microfluidic device. Images were taken every 7.5 min. TMP was added after 30 min. The movies were segmented and analyzed using a slightly adapted version of the MATLAB (MathWorks) script “SchnitzCells” (Young *et al*, 2012). To find cells and segment them we subtracted the fluorescent background of the surrounding environment (LB medium is autofluorescent) as the median fluorescence over all pixels outside of bacteria. Cell lysis was detected as a sudden disappearance of the cytosolic fluorescence signal; cells were considered intact from birth until the occurrence of such a lysis event (if any). In this way, we determined the number of intact and lysed cells at each time point. At later time points (after about 4 and 8 h, respectively, for the different TMP concentrations used), the experiments were terminated because the microfluidic chamber became crowded with cells.

#### Cellular resource allocation model

##### Derivation of the model

We built our model using Constrained Allocation Flux Balance Analysis (CAFBA) (Mori *et al*, 2016), which divides the proteome of a bacterial cell into three different sectors: ribosomal proteins (R), metabolic enzymes (E), and nutrient scavenging (C). A core feature of CAFBA is its implementation of empirical bacterial growth laws, where the R and E sectors increase their share in the proteome linearly with the growth rate in improving nutritional conditions, while the C sector follows the opposite trend, i.e., fewer nutrient‐scavenging proteins like transporters are produced in improved nutritional conditions. Here, we lump the R and E sectors of the CAFBA model into a single R sector for simplicity. Let us denote the proteome fractions of the R and C sectors as $\phi_{R}$ and $\phi_{C}$, respectively, satisfying the normalization condition $\phi_{R}+\phi_{C}=1$. We follow the formulation in CAFBA and assume that the R sector is linearly related to the growth rate:

$$\phi_{R}=\omega_{R}\lambda$$

In the original CAFBA model, the influx of nutrient substrate s is carried out by the C‐sector and takes the form $\phi_{C}\frac{s}{K_{\mathrm{Ms}}+s}$. Here, *K*_*Ms*_ is the equilibrium constant of substrate binding to the transporter, and $\frac{s}{K_{\mathrm{Ms}}+s}$ describes how the variation of s changes the saturation of this substrate binding. This influx of nutrient is then fed into the metabolic network (E‐sector) to make biomass and ultimately determines the growth rate *λ* [h^−1^]. We assumed that in the absence of the antibiotic, all nutrients are converted to biomass, and hence the growth rate is proportional to the nutrient influx, i.e.,

$$\lambda ∼\Phi_{C}\frac{s}{K_{\mathrm{Ms}}+s}.$$

In our experimental setup for glucose limitation, we maintain a constant glucose concentration and change the ratio a of αMG relative to glucose in the growth medium. As the transporter can bind to both glucose and αMG, the concentration of importable substrate *s* follows $s=c_{g}\left(a+1\right)$, where $c_{g}$ is the concentration of glucose (constant throughout the experiment), and every glucose molecule comes with a αMG molecules. Replacing s by a+1 entails the substitution of *K*_*Ms*_ by $K_{\mathrm{Ma}}≔\frac{K_{\mathrm{Ms}}}{c_{g}}$, giving $\frac{s}{K_{\mathrm{Ms}}+s}=\frac{a+1}{K_{\mathrm{Ma}}+a+1}$. Further, only a fraction $\frac{1}{a+1}$ of the imported substrates contributes to growth. These considerations lead to $\lambda ∼\phi_{C}\frac{a+1}{K_{\mathrm{Ma}}+a+1}\frac{1}{a+1}=\phi_{C}\frac{1}{K_{\mathrm{Ma}}+a+1}$. Introducing the proportionality constant $\omega_{C}$ gives $\omega_{C}\lambda =\phi_{C}\frac{1}{K_{\mathrm{Ma}}+a+1}$ or, after rearrangement,

$$\Phi_{C}=\omega_{C}\lambda \;\left(K_{\mathrm{Ma}}+a+1\right).$$

According to the CAFBA assumptions, we also have $1-\phi_{C}=\phi_{R}=\omega_{R}\lambda$, or $\phi_{C}=1-\omega_{R}\lambda$. Setting this equal to the above equation and rearranging, we can thus write the TMP‐free growth rate $\lambda_{0}$ as $\lambda_{0}\left(a|K_{\mathrm{Ma}},\omega_{R},\omega_{C}\right)=\frac{1}{\omega_{R}+\omega_{C}\left(K_{\mathrm{Ma}}+a+1\right)}$. In our case, the substrate concentration is sufficiently high and so that the C‐sector is always saturated. This means $\frac{s}{K_{\mathrm{Ms}}+s}=\frac{a+1}{K_{\mathrm{Ma}}+a+1}\approx 1$, and therefore $1\gg K_{\mathrm{Ma}}\approx 0$. $\lambda_{0}$can thus be simplified as

$$\lambda_{0}\left(a|\omega_{R},\omega_{C}\right)=\frac{1}{\omega_{R}+\omega_{C}\left(a+1\right)}.$$

In the presence of TMP at concentration c, it binds to the FolA protein according to the following reaction: FolA_free_ + TMP ⇌ FolA‐TMP. Here FolA_free_ is the free FolA protein, whereas FolA‐TMP is FolA bound to TMP. At equilibrium, we have

$$\frac{\left[\mathrm{Fol}A_{\text{free}}\right]\left[\mathrm{TMP}\right]}{\left[\text{FolA}-\mathrm{TMP}\right]}=K_{\mathrm{Mc}}$$

Let us denote the total concentration of FolA proteins in the cell as $\chi_{\text{FolA}}≔\left[\text{FolA}\right]=\left[\mathrm{Fol}A_{\text{free}}\right]+\left[\text{FolA}-\mathrm{TMP}\right].$ The concentration of free FolA can then be written as

$$\left[\mathrm{Fol}A_{\text{free}}\right]=\frac{\chi_{\text{FolA}}}{1+\frac{c}{K_{\mathrm{Mc}}}}$$

The growth rate of the cell depends on the availability of and the demand for FolA. We assume that the demand for FolA is a constant, denoted by $\chi_{\text{FolAX}}$, and that the growth rate is proportional to free FolA when its concentration is lower than the demand; otherwise, the growth rate is not affected. This then gives the growth rate equation

$$\lambda \left(a,c,\chi_{\text{FolA}}|K_{\mathrm{Mc}},\omega_{R},\omega_{C},\chi_{\text{FolAX}}\right)=\lambda_{0}\left(a|\omega_{R},\omega_{C}\right)\min\left\{1,\frac{\chi_{\text{FolA}}}{\chi_{\text{FolAX}}\left(1+\frac{c}{K_{\mathrm{Mc}}}\right)}\right\}$$

It explains the relationship between the variables λ,c,a, and $\chi_{\text{FolA}}$. While ais dimensionless, the variables ( $\lambda ,c,\chi_{\text{FolA}}$) have their own dimension. Thus, three model parameters can be eliminated by rescaling the variables using suitable units. Let us denote these rescaled variables as $\bar{\lambda },\bar{c}$, and $\bar{\chi_{\text{FolA}}}$. Starting from the growth law in the drug‐free condition

$$\lambda_{0}\left(a|\omega_{R},\omega_{C}\right)=\frac{1}{\omega_{R}+\omega_{C}\left(a+1\right)},$$

we can rearrange the terms to get

$$\omega_{R}\lambda_{0}\left(a|\omega_{R},\omega_{C}\right)=\frac{1}{1+\frac{\omega_{C}}{\omega_{R}}\left(a+1\right)}.$$

Therefore, if we define $\bar{\lambda }=\omega_{R}\lambda$ and $A=\omega_{C}/\omega_{R}$, the growth rate in the drug free condition can be written as

$$\bar{\lambda }=\frac{1}{1+A\left(a+1\right)}$$

Defining $\bar{c}=c/K_{\mathrm{Mc}}$ and $\bar{\chi_{\text{FolA}}}=\chi_{\text{FolA}}/\chi_{\text{FolAX}}$, the general form of the model becomes

$$\bar{\lambda }=\frac{1}{1+A\left(a+1\right)}\min\left\{1,\frac{\bar{\chi_{\text{FolA}}}}{1+\bar{c}}\right\}.$$

In this way, the model is simplified to have only a single meaningful fit parameter A. While the last equation provides insight into the structural dimensionality of the model, comparison with the data requires us to also fit the scaling variables $\omega_{R}$, $K_{Mc}$, and $\chi_{FolAX}$.

##### Fitting to determine the numerical values of the model parameters

As we measured the expression level of *folA* by fusing it to a fluorescent protein (Fig 4), we use the corresponding emitted light intensity to quantify $\chi_{\text{FolA}}$. We use the model to describe the behavior of cells within the range a≤10, ignoring data points with a>10. We also filtered out data points with high uncertainty, including those with growth rate <0.05 [h^−1^] or zero fluorescent intensity.

Without the drug TMP, the expression of *folA* changes with changing glucose limitation, quantified by a. If we start to increase the TMP concentration from this point, the expression of *folA* across different glucose limitations starts to converge and comes to the same value at around c=0.4 μg/ml, independent of a (Fig 4D). Therefore, we assumed that the expression of *folA* changes linearly with c within the range $c\in \left[0,0.4\right]$ at constant glucose limitation a in the model, and thereafter it becomes independent of TMP concentration for c>0.4. We implemented this strategy numerically by defining $F_{\text{FolA}}\left(a,c\right)$, the functional form to summarize the behavior of $\chi_{\text{FolA}}$. $F_{\text{FolA}}\left(a,c\right)$ is obtained by linear regression, followed by two‐dimensional interpolation of $\chi_{\text{FolA}}$ across different carbon limitations a and TMP concentrations c (Fig 4). Specifically, we used linear regression to fit $\chi_{\text{FolA}}$ vs. c at four different values of a ( a= 0, 2.5, 5, and 10, data shown in Fig 4D). If multiple measurements were performed at a given combination of a and c, we give each measurement the same weight in the regression. Thereafter, $F_{\text{FolA}}\left(a,c\right)$ was estimated through the MATLAB interpolation function “scatteredInterpolant” based on these four fitted curves. For a TMP concentration $c^{\prime }>0.4$, we set $F_{\text{FolA}}\left(a,c^{\prime }\right)=F_{\text{FolA}}\left(a,0.4\right)$.

The resulting model has four parameters: $K_{\mathrm{Mc}},\omega_{R},\omega_{C}$, and $\chi_{\text{FolAX}}$. Of these, $\omega_{R}$ and $\omega_{C}$ are associated with the simplified CAFBA model describing $\lambda_{0}\left(a|\omega_{R},\omega_{C}\right)$. To determine the free fitting parameters to the drug response, we first fit these parameters to data from measurements at negligible drug concentrations ( c< 0.1 μg/ml). We applied the MATLAB function “fit” to fit $\lambda_{0}\left(a|\omega_{R},\omega_{C}\right)$ using the same criteria to filter the data as we did in $F_{\text{FolA}}\left(a,c\right).$

The term involving $\omega_{C}$ and $\chi_{\text{FolAX}}$ modifies the $\lambda_{0}$ model to explain the drug response. We applied the same MATLAB function to fit $\lambda \left(a,c,\chi_{\text{FolA}}|K_{\mathrm{Mc}},\omega_{R},\omega_{C},\chi_{\text{FolAX}}\right)$, fixing $\omega_{R}$, and $\omega_{C}$ to the values obtained from the previous fit, and further eliminated one dynamic variable by substituting $\chi_{\text{FolA}}$ with $F_{\text{FolA}}\left(a,c\right)$. In this way, we determined all parameters of our cellular resource allocation model.

Each curve in Fig 6A and B shows the model predictions as a function of TMP concentration *c*. The dose‐sensitivity n of these curves (Fig 6, insets) was determined by fitting a Hill function exactly as for the experimental data; the error bars show 95% confidence intervals estimated from the MATLAB function “fit”. Let us denote the FolA level of the wild type at c=1 μg/ml as $\chi_{1}$. For the constant FolA condition in Fig 6B, the level of FolA was kept at $\chi_{1}$; for inverted FolA regulation, the FolA level was set to $2\chi_{1}$ at c=0, decreasing linearly to $\chi_{1}$ at c=1 μg/ml.

## Author contributions

**S Andreas Angermayr:** Conceptualization; data curation; formal analysis; validation; investigation; visualization; methodology; writing – original draft; writing – review and editing. **Tin Yau Pang:** Formal analysis; investigation; writing – review and editing. **Guillaume Chevereau:** Data curation; formal analysis; writing – review and editing. **Karin Mitosch:** Investigation; methodology; writing – review and editing. **Martin J Lercher:** Formal analysis; supervision; writing – review and editing. **Tobias Bollenbach:** Conceptualization; resources; data curation; formal analysis; supervision; funding acquisition; validation; investigation; visualization; writing – original draft; project administration; writing – review and editing.

## Disclosure and competing interests statement

The authors declare that they have no conflict of interest.

## Supporting information




Appendix

Table EV1

Source Data for Figure 1

Source Data for Figure 2

Source Data for Figure 3

Source Data for Figure 4

Source Data for Figure 5

Source Data for Appendix

## Data Availability

This study includes no data deposited in external repositories. All essential data are available as Source Data.

## References

[msb202110490-bib-0001] Baba T , Ara T , Hasegawa M , Takai Y , Okumura Y , Baba M , Datsenko KA , Tomita M , Wanner BL , Mori H (2006) Construction of *Escherichia coli* K‐12 in‐frame, single‐gene knockout mutants: the Keio collection. Mol Syst Biol 2: 20060008 10.1038/msb4100050 PMC1681482 16738554

[msb202110490-bib-0002] Balaban NQ , Merrin J , Chait R , Kowalik L , Leibler S (2004) Bacterial persistence as a phenotypic switch. Science 305: 1622–1625 15308767 10.1126/science.1099390

[msb202110490-bib-0003] Baym M , Lieberman TD , Kelsic ED , Chait R , Gross R , Yelin I , Kishony R (2016) Spatiotemporal microbial evolution on antibiotic landscapes. Science 353: 1147–1151 27609891 10.1126/science.aag0822 PMC5534434

[msb202110490-bib-0004] Bershtein S , Choi J‐M , Bhattacharyya S , Budnik B , Shakhnovich E (2015) Systems‐level response to point mutations in a core metabolic enzyme modulates genotype‐phenotype relationship. Cell Rep 11: 645–656 25892240 10.1016/j.celrep.2015.03.051 PMC4416983

[msb202110490-bib-0005] Bershtein S , Mu W , Serohijos AWR , Zhou J , Shakhnovich EI (2013) Protein quality control acts on folding intermediates to shape the effects of mutations on organismal fitness. Mol Cell 49: 133–144 23219534 10.1016/j.molcel.2012.11.004 PMC3545112

[msb202110490-bib-0006] Bhattacharyya S , Bershtein S , Yan J , Argun T , Gilson AI , Trauger SA , Shakhnovich EI (2016) Transient protein‐protein interactions perturb *E. coli* metabolome and cause gene dosage toxicity. Elife 5: 1–22 10.7554/eLife.20309 PMC5176355 27938662

[msb202110490-bib-0007] Bintu L , Buchler NE , Garcia HG , Gerland U , Hwa T , Kondev J , Phillips R (2005) Transcriptional regulation by the numbers: models. Curr Opin Genet Dev 15: 116–124 15797194 10.1016/j.gde.2005.02.007 PMC3482385

[msb202110490-bib-0008] Bollenbach T , Quan S , Chait R , Kishony R (2009) Nonoptimal microbial response to antibiotics underlies suppressive drug interactions. Cell 139: 707–718 19914165 10.1016/j.cell.2009.10.025 PMC2838386

[msb202110490-bib-0009] Bremer H , Dennis PP (2008) Modulation of chemical composition and other parameters of the cell at different exponential growth rates. EcoSal Plus 3: 765–777 10.1128/ecosal.5.2.3 26443740

[msb202110490-bib-0010] Chait R , Craney A , Kishony R (2007) Antibiotic interactions that select against resistance. Nature 446: 668–671 17410176 10.1038/nature05685

[msb202110490-bib-0011] Cherepanov PP , Wackernagel W (1995) Gene disruption in *Escherichia coli*: TcR and KmR cassettes with the option of Flp‐catalyzed excision of the antibiotic‐resistance determinant. Gene 158: 9–14 7789817 10.1016/0378-1119(95)00193-a

[msb202110490-bib-0012] Chevereau G , Bollenbach T (2015) Systematic discovery of drug interaction mechanisms. Mol Syst Biol 11: 807 25924924 10.15252/msb.20156098 PMC4422561

[msb202110490-bib-0013] Chevereau G , Dravecká M , Batur T , Guvenek A , Ayhan DH , Toprak E , Bollenbach T (2015) Quantifying the determinants of evolutionary dynamics leading to drug resistance. PLoS Biol 13: e1002299 26581035 10.1371/journal.pbio.1002299 PMC4651364

[msb202110490-bib-0014] Chou T‐C , Talalay P (1983) Analysis of combined drug effects: a new look at a very old problem. Trends Pharmacol Sci 4: 450–454

[msb202110490-bib-0015] Datsenko KA , Wanner BL (2000) One‐step inactivation of chromosomal genes in *Escherichia coli* K‐12 using PCR products. Proc Natl Acad Sci USA 97: 6640–6645 10829079 10.1073/pnas.120163297 PMC18686

[msb202110490-bib-0016] Datta S , Costantino N , Court DL (2006) A set of recombineering plasmids for gram‐negative bacteria. Gene 379: 109–115 16750601 10.1016/j.gene.2006.04.018

[msb202110490-bib-0017] Deris JB , Kim M , Zhang Z , Okano H , Hermsen R , Groisman A , Hwa T (2013) The innate growth bistability and fitness landscapes of antibiotic‐resistant bacteria. Science 342: 1237435 24288338 10.1126/science.1237435 PMC4059556

[msb202110490-bib-0018] Dong H , Nilsson L , Kurland C (1995) Gratuitous overexpression of genes in *Escherichia coli* leads to growth inhibition and ribosome destruction. J Bacteriol 177: 1497–1504 7883706 10.1128/jb.177.6.1497-1504.1995 PMC176765

[msb202110490-bib-0019] Elf J , Nilsson K , Tenson T , Ehrenberg M (2006) Bistable bacterial growth rate in response to antibiotics with low membrane permeability. Phys Rev Lett 97: 1–4 10.1103/PhysRevLett.97.258104 17280399

[msb202110490-bib-0020] Elowitz MB , Leibler S (2000) A synthetic oscillatory network of transcriptional regulators. Nature 403: 335–338 10659856 10.1038/35002125

[msb202110490-bib-0021] Flensburg J , Sköld O (1987) Massive overproduction of dihydrofolate reductase in bacteria as a response to the use of trimethoprim. Eur J Biochem 162: 473–476 3549289 10.1111/j.1432-1033.1987.tb10664.x

[msb202110490-bib-0022] Garcia HG , Lee HJ , Boedicker JQ , Phillips R (2011) Comparison and calibration of different reporters for quantitative analysis of gene expression. Biophys J 101: 535–544 21806921 10.1016/j.bpj.2011.06.026 PMC3145315

[msb202110490-bib-0023] Gardner TS , Cantor CR , Collins JJ (2000) Construction of a genetic toggle switch in *Escherichia coli* . Nature 403: 339–342 10659857 10.1038/35002131

[msb202110490-bib-0024] Godin M , Delgado FF , Son S , Grover WH , Bryan AK , Tzur A , Jorgensen P , Payer K , Grossman AD , Kirschner MW *et al* (2010) Using buoyant mass to measure the growth of single cells. Nat Methods 7: 387–390 20383132 10.1038/nmeth.1452 PMC2862099

[msb202110490-bib-0025] Greulich P , Scott M , Evans MR , Allen RJ (2015) Growth‐dependent bacterial susceptibility to ribosome‐targeting antibiotics. Mol Syst Biol 11: 796 26146675 10.15252/msb.20145949 PMC4380930

[msb202110490-bib-0026] Hansen MT , Pato ML , Molin S , Fill NP , von Meyenburg K (1975) Simple downshift and resulting lack of correlation between ppGpp pool size and ribonucleic acid accumulation. J Bacteriol 122: 585–591 1092659 10.1128/jb.122.2.585-591.1975 PMC246095

[msb202110490-bib-0027] Hansson MD , Rzeznicka K , Rosenbäck M , Hansson M , Sirijovski N (2008) PCR‐mediated deletion of plasmid DNA. Anal Biochem 375: 373–375 18157935 10.1016/j.ab.2007.12.005

[msb202110490-bib-0028] Heckman KL , Pease LR (2007) Gene splicing and mutagenesis by PCR‐driven overlap extension. Nat Protoc 2: 924–932 17446874 10.1038/nprot.2007.132

[msb202110490-bib-0029] Hermsen R , Deris JB , Hwa T (2012) On the rapidity of antibiotic resistance evolution facilitated by a concentration gradient. Proc Natl Acad Sci USA 109: 10775–10780 22711808 10.1073/pnas.1117716109 PMC3390829

[msb202110490-bib-0030] Hol FJH , Hubert B , Dekker C , Keymer JE (2016) Density‐dependent adaptive resistance allows swimming bacteria to colonize an antibiotic gradient. ISME J 10: 30–38 26140531 10.1038/ismej.2015.107 PMC4681852

[msb202110490-bib-0031] Kavčič B , Tkačik G , Bollenbach T (2020) Mechanisms of drug interactions between translation‐inhibiting antibiotics. Nat Commun 11: 4013 32782250 10.1038/s41467-020-17734-z PMC7421507

[msb202110490-bib-0032] Keseler IM (2004) EcoCyc: a comprehensive database resource for *Escherichia coli* . Nucleic Acids Res 33: D334–D337 10.1093/nar/gki108 PMC540062 15608210

[msb202110490-bib-0033] Kishony R , Leibler S (2003) Environmental stresses can alleviate the average deleterious effect of mutations. J Biol 2: 14 12775217 10.1186/1475-4924-2-14 PMC193686

[msb202110490-bib-0034] Kitagawa M , Ara T , Arifuzzaman M , Ioka‐Nakamichi T , Inamoto E , Toyonaga H , Mori H (2005) Complete set of ORF clones of *Escherichia coli* ASKA library (A complete set of *E. coli* K‐12 ORF archive): unique resources for biological research. DNA Res 12: 291–299 16769691 10.1093/dnares/dsi012

[msb202110490-bib-0035] Klumpp S , Zhang Z , Hwa T (2009) Growth rate‐dependent global effects on gene expression in bacteria. Cell 139: 1366–1375 20064380 10.1016/j.cell.2009.12.001 PMC2818994

[msb202110490-bib-0036] Kwon YK , Higgins MB , Rabinowitz JD (2010) Antifolate‐induced depletion of intracellular glycine and purines inhibits thymineless death in *E. coli* . ACS Chem Biol 5: 787–795 20553049 10.1021/cb100096f PMC2945287

[msb202110490-bib-0037] Lee AJ , Wang S , Meredith HR , Zhuang B , Dai Z , You L (2018) Robust, linear correlations between growth rates and β‐lactam‐mediated lysis rates. Proc Natl Acad Sci USA 115: 4069–4074 29610312 10.1073/pnas.1719504115 PMC5910845

[msb202110490-bib-0038] Liu H , Naismith JH (2008) An efficient one‐step site‐directed deletion, insertion, single and multiple‐site plasmid mutagenesis protocol. BMC Biotechnol 8: 91 19055817 10.1186/1472-6750-8-91 PMC2629768

[msb202110490-bib-0039] Lopatkin AJ , Stokes JM , Zheng EJ , Yang JH , Takahashi MK , You L , Collins JJ (2019) Bacterial metabolic state more accurately predicts antibiotic lethality than growth rate. Nat Microbiol 4: 2109–2117 31451773 10.1038/s41564-019-0536-0 PMC6879803

[msb202110490-bib-0040] Lutz R , Bujard H (1997) Independent and tight regulation of transcriptional units in *Escherichia coli* via the LacR/O, the TetR/O and AraC/I1‐I2 regulatory elements. Nucleic Acids Res 25: 1203–1210 9092630 10.1093/nar/25.6.1203 PMC146584

[msb202110490-bib-0041] Martinez KA , Kitko RD , Mershon JP , Adcox HE , Malek KA , Berkmen MB , Slonczewski JL (2012) Cytoplasmic pH response to acid stress in individual cells of *Escherichia coli* and *Bacillus subtilis* observed by fluorescence ratio imaging microscopy. Appl Environ Microbiol 78: 3706–3714 22427503 10.1128/AEM.00354-12 PMC3346368

[msb202110490-bib-0042] Mitosch K , Rieckh G , Bollenbach T (2017) Noisy response to antibiotic stress predicts subsequent single‐cell survival in an acidic environment. Cell Syst 4: 393–403 28342718 10.1016/j.cels.2017.03.001

[msb202110490-bib-0043] Mitosch K , Rieckh G , Bollenbach T (2019) Temporal order and precision of complex stress responses in individual bacteria. Mol Syst Biol 15: e8470 30765425 10.15252/msb.20188470 PMC6375286

[msb202110490-bib-0044] Mori M , Hwa T , Martin OC , De Martino A , Marinari E (2016) Constrained allocation flux balance analysis. PLoS Comp Biol 12: e1004913 10.1371/journal.pcbi.1004913 PMC4927118 27355325

[msb202110490-bib-0045] Nevozhay D , Adams RM , Murphy KF , Josic K , Balázsi G (2009) Negative autoregulation linearizes the dose‐response and suppresses the heterogeneity of gene expression. Proc Natl Acad Sci USA 106: 5123–5128 19279212 10.1073/pnas.0809901106 PMC2654390

[msb202110490-bib-0046] Nichols RJ , Sen S , Choo YJ , Beltrao P , Zietek M , Chaba R , Lee S , Kazmierczak KM , Lee KJ , Wong A *et al* (2011) Phenotypic landscape of a bacterial cell. Cell 144: 143–156 21185072 10.1016/j.cell.2010.11.052 PMC3060659

[msb202110490-bib-0047] Nyerges Á , Csörgő B , Draskovits G , Kintses B , Szili P , Ferenc G , Révész T , Ari E , Nagy I , Bálint B *et al* (2018) Directed evolution of multiple genomic loci allows the prediction of antibiotic resistance. Proc Natl Acad Sci USA 115: E5726–E5735 29871954 10.1073/pnas.1801646115 PMC6016788

[msb202110490-bib-0048] Palmer AC , Kishony R (2014) Opposing effects of target overexpression reveal drug mechanisms. Nat Commun 5: 4296 24980690 10.1038/ncomms5296 PMC4408919

[msb202110490-bib-0049] Regoes RR , Wiuff C , Zappala RM , Garner KN , Baquero F , Levin BR (2004) Pharmacodynamic functions: a multiparameter approach to the design of antibiotic treatment regimens. Antimicrob Agents Chemother 48: 3670–3676 15388418 10.1128/AAC.48.10.3670-3676.2004 PMC521919

[msb202110490-bib-0050] Rodrigues JV , Bershtein S , Li A , Lozovsky ER , Hartl DL , Shakhnovich EI (2016) Biophysical principles predict fitness landscapes of drug resistance. Proc Natl Acad Sci USA 113: E1470–E1478 26929328 10.1073/pnas.1601441113 PMC4801265

[msb202110490-bib-0051] Rood JI , Laird AJ , Williams JW (1980) Cloning of the *Escherichia coli* K‐12 dihydrofolate reductase gene following mu‐mediated transposition. Gene 8: 255–265 6444603 10.1016/0378-1119(80)90003-7

[msb202110490-bib-0052] Russ D , Kishony R (2018) Additivity of inhibitory effects in multidrug combinations. Nat Microbiol 3: 1339–1345 30323252 10.1038/s41564-018-0252-1 PMC6295580

[msb202110490-bib-0053] Scott M , Gunderson CW , Mateescu EM , Zhang Z , Hwa T (2010) Interdependence of cell growth and gene expression: origins and consequences. Science 330: 1099–1102 21097934 10.1126/science.1192588

[msb202110490-bib-0054] Sharan SK , Thomason LC , Kuznetsov SG , Court DL (2009) Recombineering: a homologous recombination‐based method of genetic engineering. Nat Protoc 4: 206–223 19180090 10.1038/nprot.2008.227 PMC2790811

[msb202110490-bib-0055] Soo VWC , Hanson‐Manful P , Patrick WM (2011) Artificial gene amplification reveals an abundance of promiscuous resistance determinants in *Escherichia coli* . Proc Natl Acad Sci USA 108: 1484–1489 21173244 10.1073/pnas.1012108108 PMC3029738

[msb202110490-bib-0056] Toprak E , Veres A , Michel J‐B , Chait R , Hartl DL , Kishony R (2012) Evolutionary paths to antibiotic resistance under dynamically sustained drug selection. Nat Genet 44: 101–105 10.1038/ng.1034 PMC3534735 22179135

[msb202110490-bib-0057] Tuomanen E , Cozens R , Tosch W , Zak O , Tomasz A (1986) The rate of killing of *Escherichia coli* by beta‐lactam antibiotics is strictly proportional to the rate of bacterial growth. J Gen Microbiol 132: 1297–1304 3534137 10.1099/00221287-132-5-1297

[msb202110490-bib-0058] Yang J , Ogawa Y , Camakaris H , Shimada T , Ishihama A , Pittard AJ (2007) folA, a new member of the TyrR regulon in *Escherichia coli* K‐12. J Bacteriol 189: 6080–6084 17557822 10.1128/JB.00482-07 PMC1952039

[msb202110490-bib-0059] Young JW , Locke JCW , Altinok A , Rosenfeld N , Bacarian T , Swain PS , Mjolsness E , Elowitz MB (2012) Measuring single‐cell gene expression dynamics in bacteria using fluorescence time‐lapse microscopy. Nat Protoc 7: 80–88 10.1038/nprot.2011.432 PMC4161363 22179594

[msb202110490-bib-0060] Zaslaver A , Bren A , Ronen M , Itzkovitz S , Kikoin I , Shavit S , Liebermeister W , Surette MG , Alon U (2006) A comprehensive library of fluorescent transcriptional reporters for *Escherichia coli* . Nat Methods 3: 623–628 16862137 10.1038/nmeth895

