# Supplementary material for: Growth‐mediated negative feedback shapes quantitative antibiotic response
Source: Mol Syst Biol. 2022 Sep 20;18(9):e10490. doi: 10.15252/msb.202110490 (PMC9486506; doi:10.15252/msb.202110490)
Supplement: Supplementary file 1 — Appendix [file MSB-18-e10490-s009.pdf]

Appendix

Growth-mediated negative feedback shapes quantitative antibiotic response

S. Andreas Angermayr[1,2,#], Tin Yau Pang[3,4], Guillaume Chevereau[5], Karin Mitosch[2,6], Martin J. Lercher[3,4], Tobias Bollenbach[1,7*]

[1]Institute for Biological Physics, University of Cologne, Cologne, Germany

[2]Institute of Science and Technology Austria, Klosterneuburg, Austria

[3]Institute for Computer Science, Heinrich Heine University Düsseldorf, Düsseldorf, Germany

[4]Department of Biology, Heinrich Heine University Düsseldorf, Düsseldorf, Germany

[5]INSA de Strasbourg, Strasbourg, France

[6]Genome Biology Unit, European Molecular Biology Laboratory (EMBL), Heidelberg, Germany

[7]Center for Data and Simulation Science, University of Cologne, Cologne, Germany

[#]Present address: CeMM Research Center for Molecular Medicine of the Austrian Academy of Sciences, Vienna, Austria.

[*]Corresponding author, t.bollenbach@uni-koeln.de

Contents

Appendix Figure S1|Correlation of antibiotic response and drug-free growth rate for genome-wide gene deletion strains. 3

Appendix Figure S2|Correlation of IC_{50} and drug-free growth rate in gene deletion strains. 4

Appendix Figure S3| Day-to-day reproducibility of growth rate measurements. 5

Appendix Figure S4| IPTG alone at the concentrations used here has no effect on growth rate. 6

Appendix Figure S5| Effect of growth rate reduction by glucose limitation on susceptibility to diverse antibiotics. 7

Appendix Figure S6| Effect of growth rate reduction by gratuitous protein overexpression on susceptibility to antibiotics. 8

Appendix Figure S7| Effect of growth rate reduction by changing carbon source on susceptibility to antibiotics. 9

Appendix Figure S8|Lowering growth rate by changing temperature does not affect the shape of antibiotic dose-response curves. 10

Appendix Figure S9|Expression level of folA as a function of trimethoprim and αMG/glucose ratio. 11

Appendix Figure S10|Growth rate decrease due to DHFR overexpression is rescued by trimethoprim. 12

Appendix Figure S11| Bacterial growth curves from optical density measurements. 13

Appendix Figure S12| Dose-response curves calculated from mathematical model for different levels of glucose limitation. 14

Appendix Figure S13| Unscaled dose-response curves. 15

Appendix Figure S14| Non-normalized dose-response curves. 16

Appendix Figure S15| Effect of slower growth on trimethoprim efficacy is also observed for the IC_{50} and for alternative normalization of the dose-response data. 17

Appendix Figure S16| Trimethoprim causes almost no cell death at concentrations below the MIC. 18

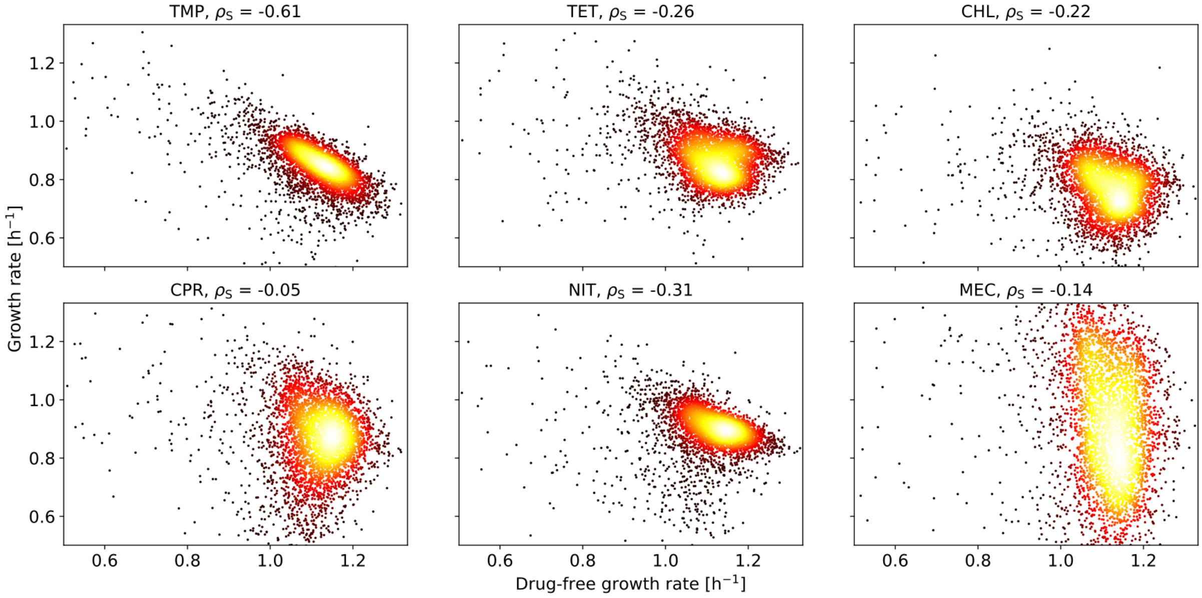

Appendix Figure S1 | Correlation of antibiotic response and drug-free growth rate for genome-wide gene deletion strains. *Density scatterplots showing growth response to different antibiotics versus normalized growth rate in the absence of drug for genome-wide gene deletion strains* (Baba *et al*, 2006) *as in Fig. 1C. Response is defined as growth rate in the presence of the respective drug normalized to the drug-free growth rate of the respective deletion strain. Each drug was used at a fixed drug concentration that inhibits wild type growth by about 30%* (Chevereau *et al*, 2015). *Spearman correlation coefficient* ρ_s *is shown.*

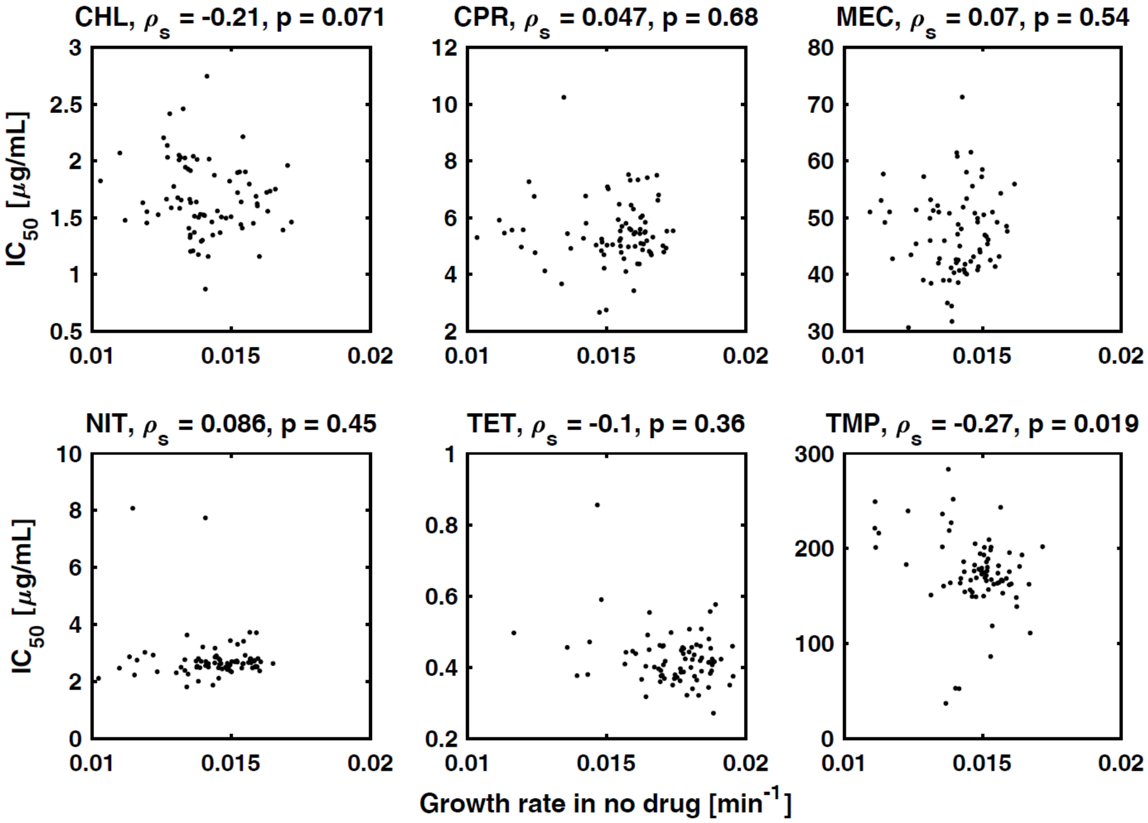

Appendix Figure S2|Correlation of IC_{50} and drug-free growth rate in gene deletion strains.
Scatterplots of the IC_{50} of gene deletion mutants versus the growth rate of these mutants in the absence of drug; each panel shows a different antibiotic as labeled. ρ_S is the Spearman correlation; p-values of this correlation from permutation test are shown. The only significant (negative) correlation occurs for TMP, consistent with growth-mediated negative feedback for this drug. IC_{50}s were determined from dose-response curve measurements of 78 arbitrary gene deletions strains (Chevereau *et al*, 2015).

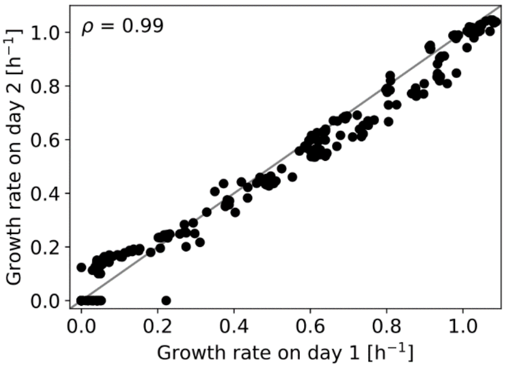

Appendix Figure S3| Day-to-day reproducibility of growth rate measurements.
Scatterplot showing comparison of growth rate data from αMG-TMP two-dimensional concentration gradient experiment (Fig. 2B) performed on two different days. Pearson's correlation coefficient ρ is shown.

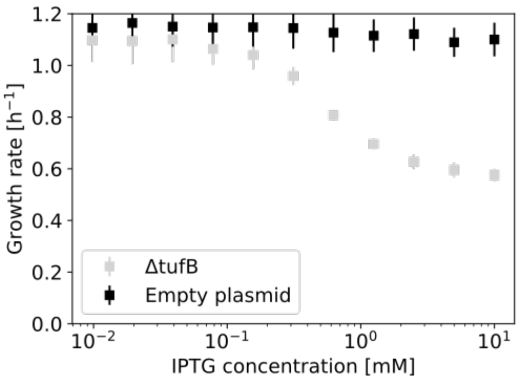

Appendix Figure S4| IPTG alone at the concentrations used here has no effect on growth rate.
Black data points show growth rate versus IPTG concentration for a control strain with an empty expression vector; data from Fig. 2C is shown in gray for comparison. Error bars show standard deviation from eight biological replicates.

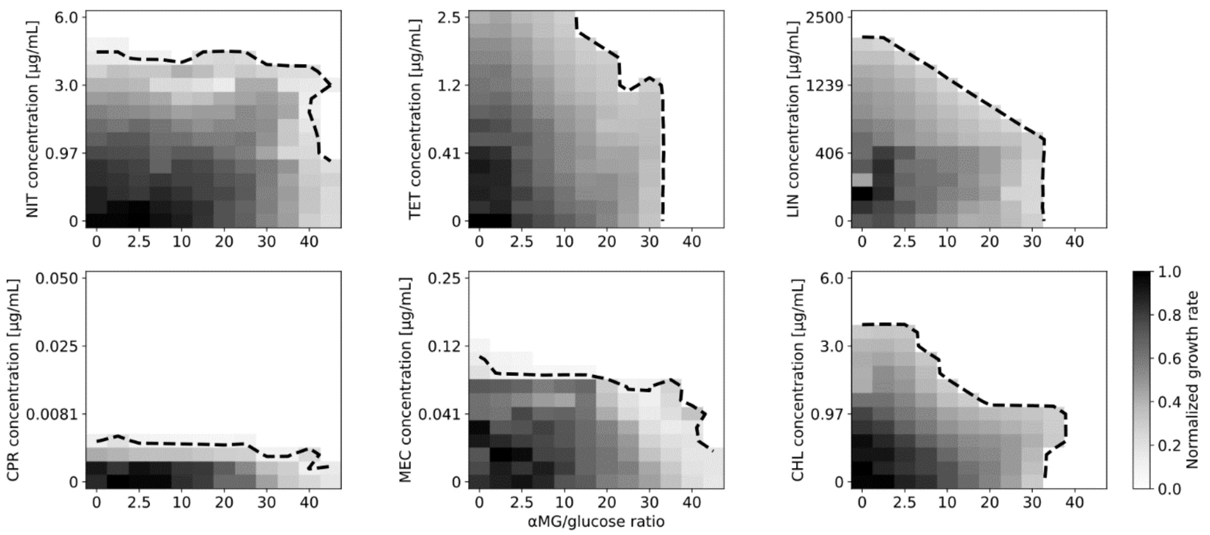

Appendix Figure S5| Effect of growth rate reduction by glucose limitation on susceptibility to diverse antibiotics. *As Fig. 2B, for nitrofurantoin (NIT), tetracycline (TET), lincomycin (LIN), ciprofloxacin (CPR), mecillinam (MEC), and chloramphenicol (CHL). Lowering growth rate by glucose limitation via αMG does not lower susceptibility to these antibiotics as for TMP (cf. Fig. 2C).*

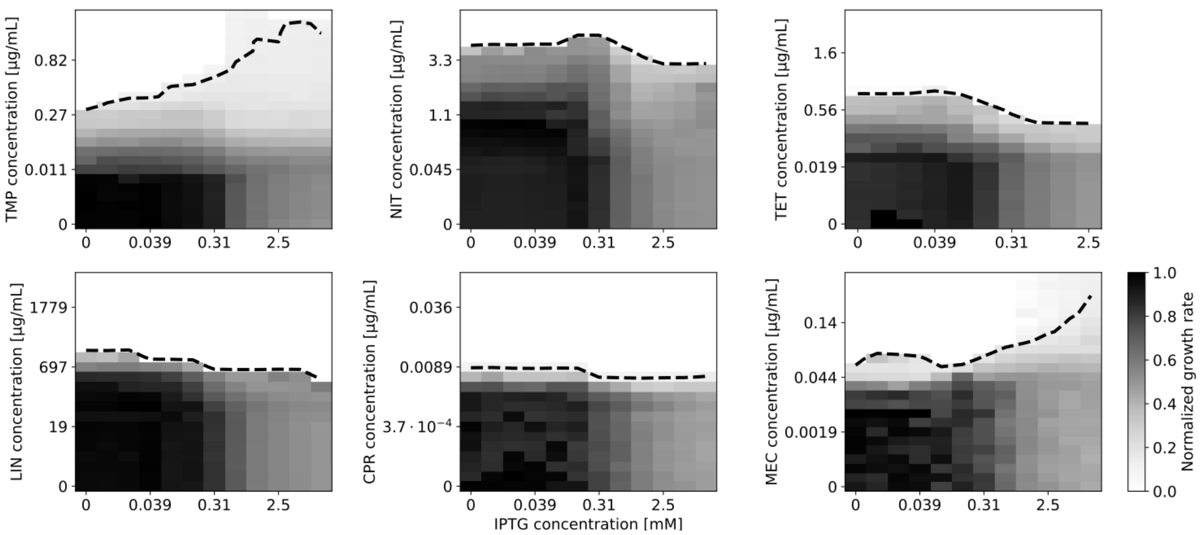

Appendix Figure S6| Effect of growth rate reduction by gratuitous protein overexpression on susceptibility to antibiotics. *As Fig. 2E, for trimethoprim (TMP), nitrofurantoin (NIT), tetracycline (TET), lincomycin (LIN), ciprofloxacin (CPR), and mecillinam (MEC). Lowering growth rate by gratuitous protein overexpression lowers susceptibility to TMP and, to a lesser extent, to MEC (bottom right), but not for the other antibiotics (cf. Fig. 2F).*

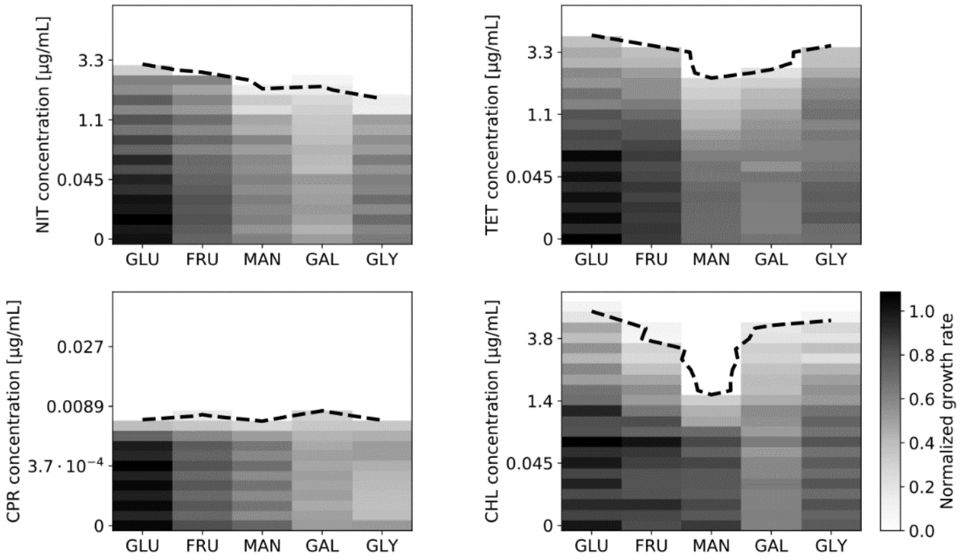

Appendix Figure S7| Effect of growth rate reduction by changing carbon source on susceptibility to antibiotics.
As Fig. 2H, for nitrofurantoin (NIT), tetracycline (TET), ciprofloxacin (CPR), and chloramphenicol (CHL). Lowering growth rate via poorer carbon sources does not lower susceptibility to other antibiotics than TMP (cf. Fig. 2I). Data shown is the mean of three biological replicates. We also performed this assay for mecillinam (MEC), but excluded it from further analysis because – for unknown reasons – it consistently showed extremely noisy dose-response curves in this assay.

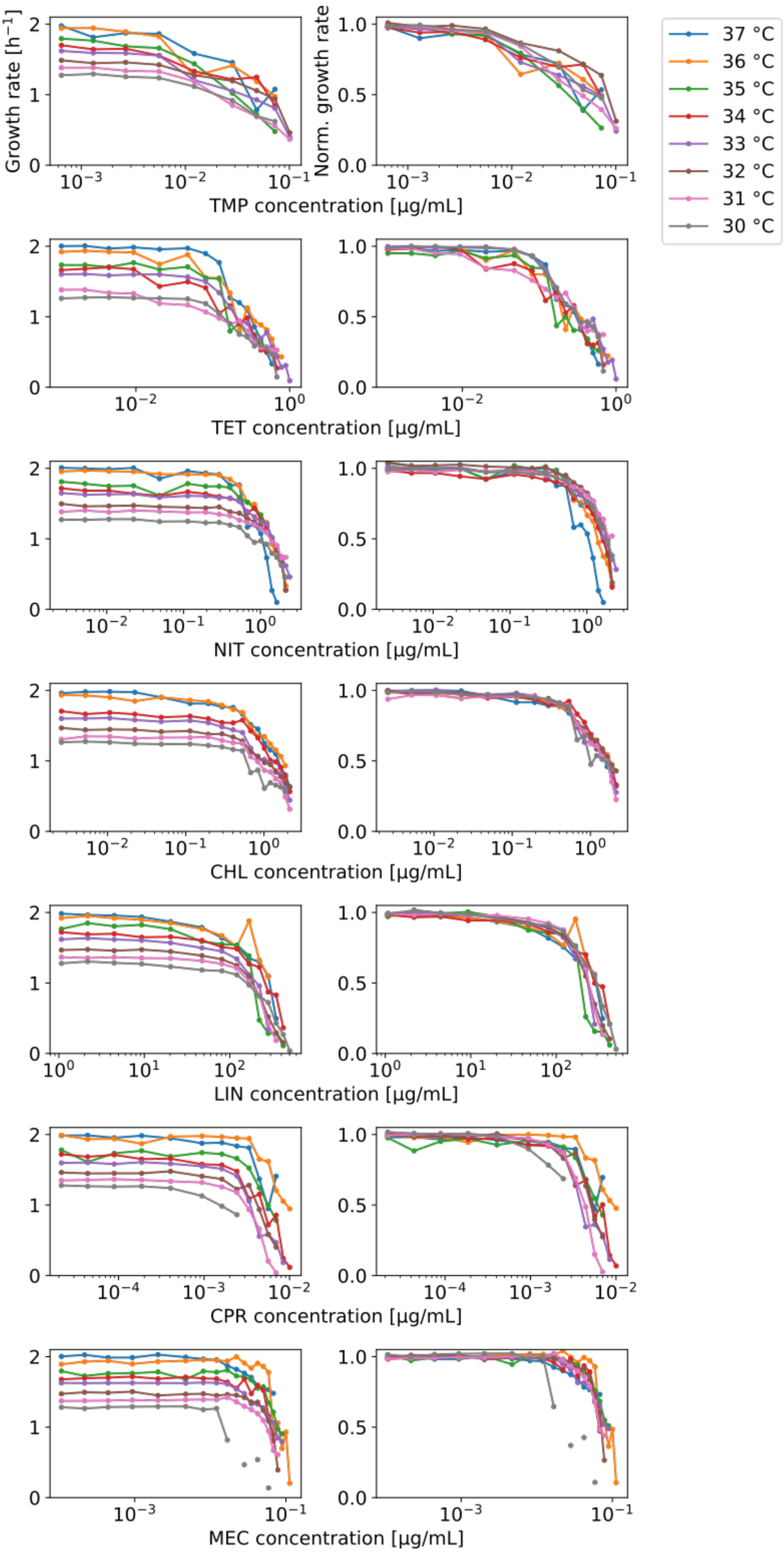

Appendix Figure S8|Lowering growth rate by changing temperature does not affect the shape of antibiotic dose-response curves.

Left column: Growth rate versus drug concentration for eight different antibiotics at eight different temperatures as shown. Right column: Growth rate normalized to drug-free growth rate.

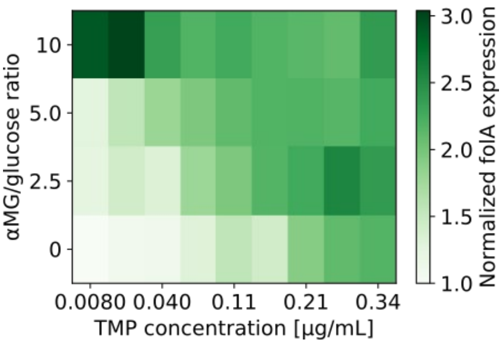

Appendix Figure S9|Expression level of folA as a function of trimethoprim and αMG/glucose ratio.
Data from Fig. 4 in two-dimensional checkerboard plot.

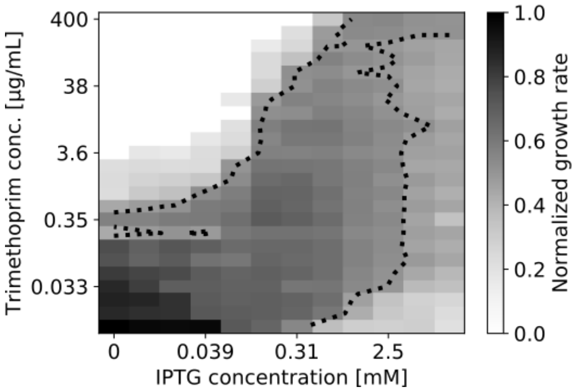

Appendix Figure S10|Growth rate decrease due to DHFR overexpression is rescued by trimethoprim.
Normalized growth rate (gray scale) in a two-dimensional concentration gradient of IPTG and TMP. IPTG controls overexpression of folA (Materials and Methods). Dotted lines are contour lines at 50% growth inhibition. DHFR overexpression lowers growth rate but adding TMP at high IPTG concentrations partially rescues this phenotype: Growth rate increases with increasing TMP concentration. The increase in TMP IC_{50} *resulting from DHFR overexpression confirms previous reports (Palmer & Kishony, 2014).*

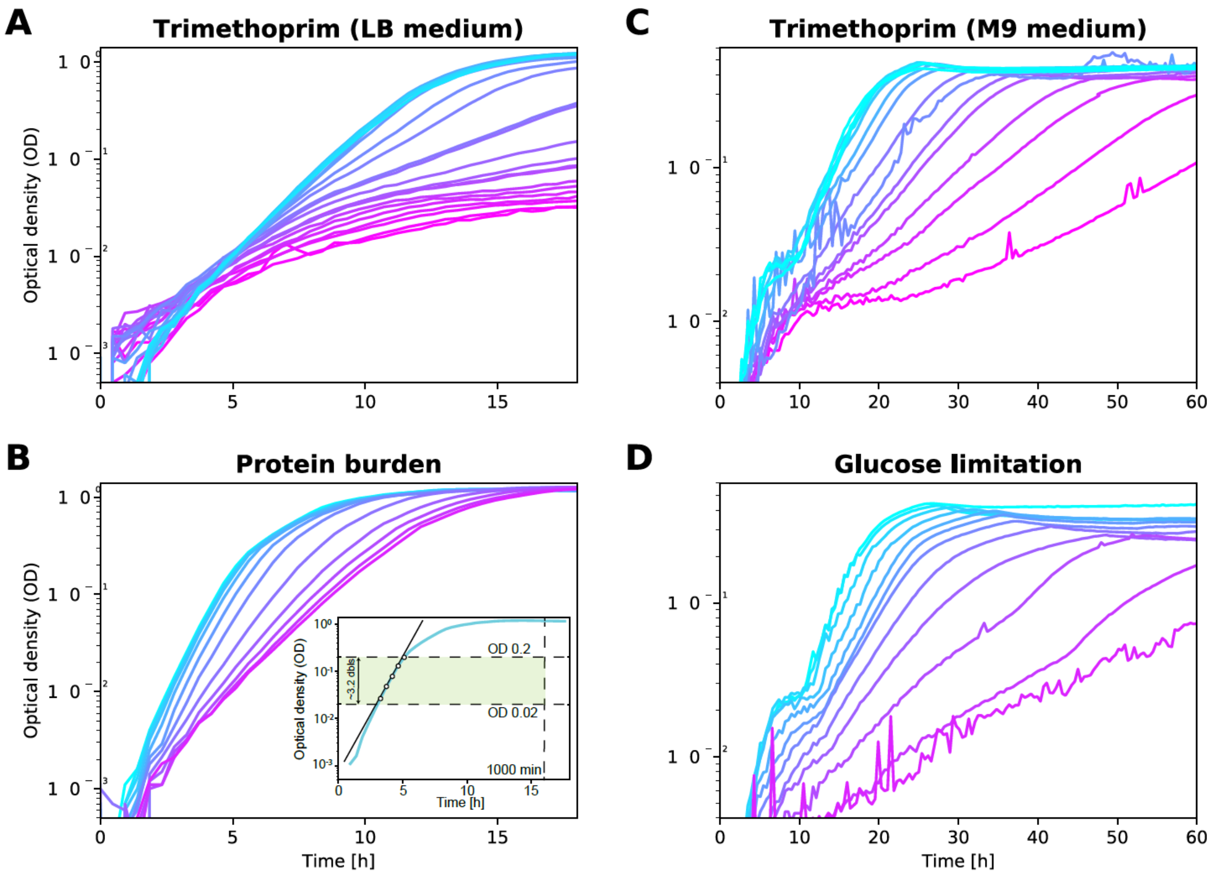

Appendix Figure S11| Bacterial growth curves from optical density measurements.
Representative data for different ways of lowering the growth rate in different growth media.
***(A)** TMP in LB medium. Growth curves underlying the data shown in the leftmost column of Fig. 2E; TMP concentration increases from cyan to magenta.*
***(B)** Gratuitous protein overexpression in LB medium; data correspond to Fig. 2D; IPTG concentration increases from cyan to magenta.*
***(C)** TMP in glucose (minimal M9) medium; data correspond to the leftmost column of Fig. 2B.*
***(D)** αMG in glucose (minimal M9) medium; data correspond to Fig. 2A; αMG/glucose ratio increases from cyan to magenta.*
Inset in B: Schematic showing growth rate quantification by a linear fit of the log-transformed and background-subtracted OD data from exponential growth phase (Materials and Methods), essentially as described in (Chevereau & Bollenbach, 2015). Here, OD windows used for cultures in LB medium were 0.02 to 0.2 (3.2 doublings), and for minimal medium 0.03 to 0.12 (2 doublings), taking into account the lower growth yield in minimal medium. For LB medium, data after ~1,000 min were discarded to avoid including fast-growing mutants that may occur sporadically in the presence of antibiotics; this was not necessary for experiments in minimal medium.

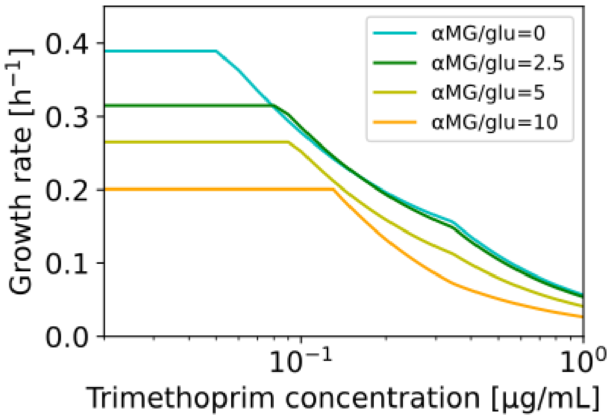

Appendix Figure S12| Dose-response curves calculated from mathematical model for different levels of glucose limitation.

As Fig. 6A, but showing non-normalized growth rate; αMG/glucose ratio is shown in legend. At moderate glucose limitation (αMG/glucose = 2.5, green line), the growth rate can be slightly higher than without this limitation (cyan line), as observed experimentally (cf. Fig. 2B).

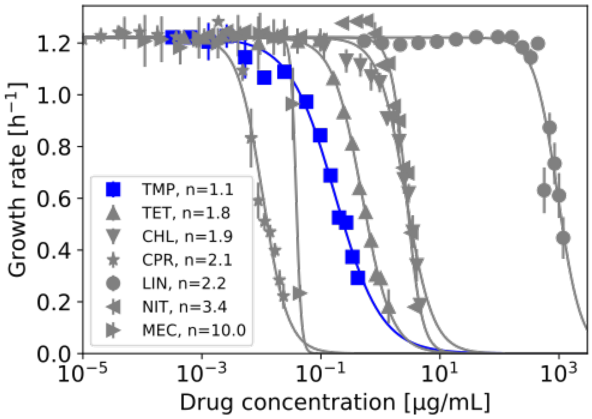

Appendix Figure S13| Unscaled dose-response curves.
As Fig. 1A but with absolute drug concentrations on the x-axis.

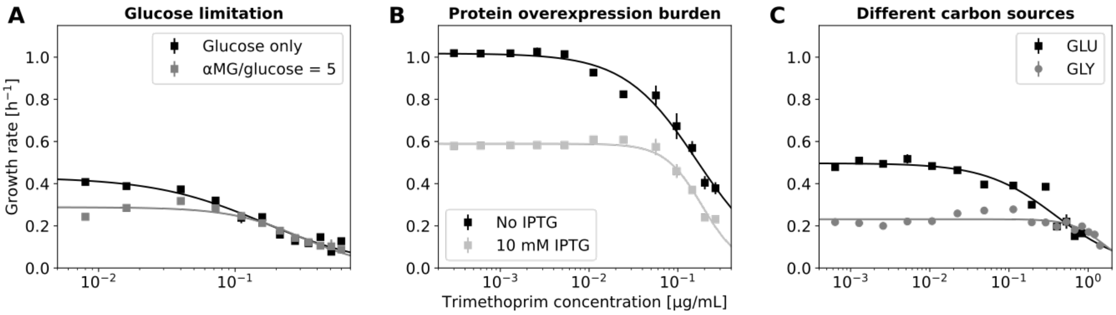

Appendix Figure S14| Non-normalized dose-response curves.
As Fig. 3A,C,E, but with absolute growth rate on the y-axis.

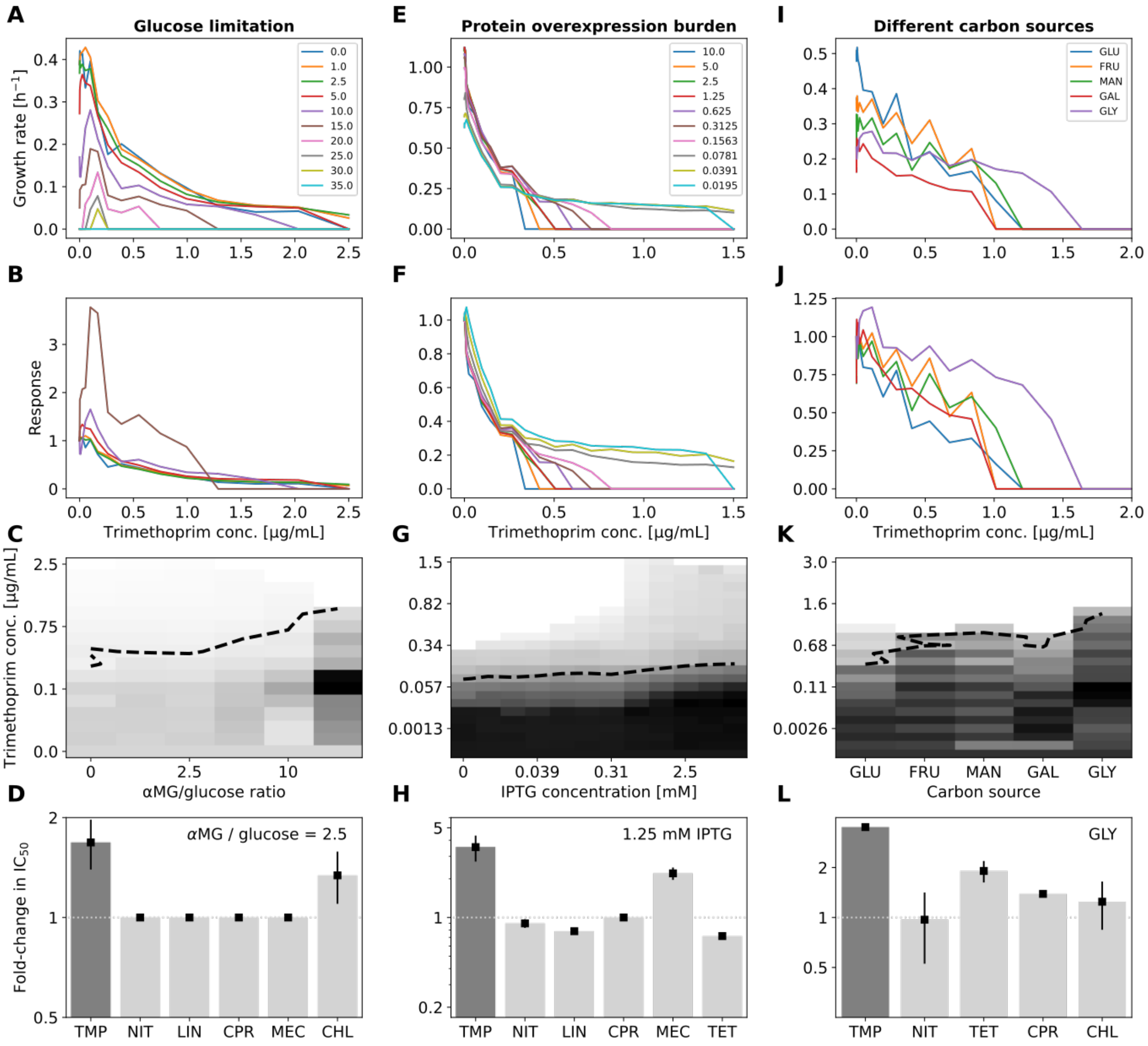

Appendix Figure S15| Effect of slower growth on trimethoprim efficacy is also observed for the IC_{50} and for alternative normalization of the dose-response data.

(A) *Data from Fig. 2B shown as TMP dose-response curves at different αMG/glucose ratios as shown in legend.*

(B) *As A, but with each dose-response curve normalized to the growth rate in the absence of drug (response).*

(C) *As Fig. 2B but using the normalization in B. Dashed line shows isobole of 50% growth inhibition. Note that this normalization is not possible for the higher αMG/glucose ratios shown in Fig. 2B, which lead to zero growth in the absence of TMP.*

(D) *Fold-change in IC_{50} as determined from dose-response curves in B; other antibiotics are shown for comparison (as in Fig. 2C).*

(E-H) *As A-D but for the data in Fig. 2E (protein over-expression burden).*

(I-L) *As A-D but for the data in Fig. 2H (different carbon sources).*

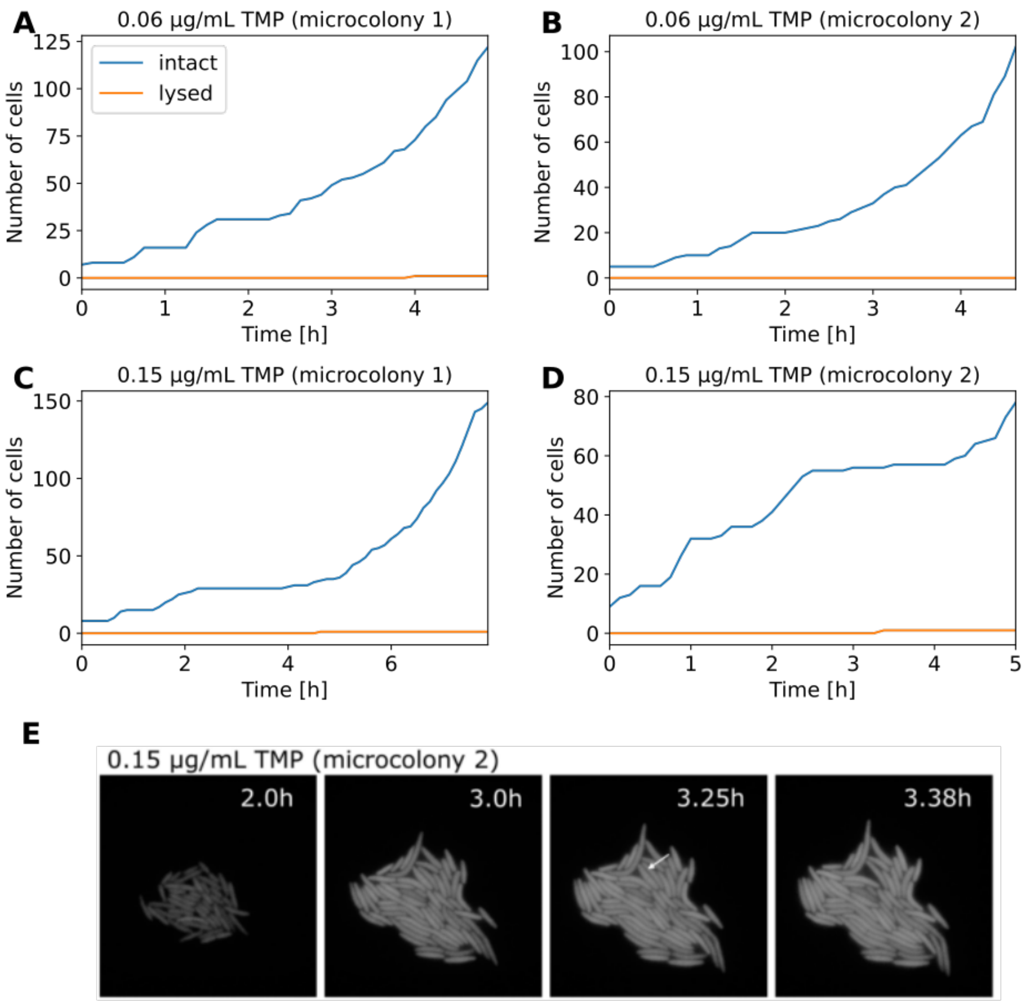

Appendix Figure S16| Trimethoprim causes almost no cell death at concentrations below the MIC.
(A-D) *Numbers of intact and lysed cells in microcolonies observed by time-lapse imaging in a microfluidic chamber in LB medium containing TMP at different concentrations (0.06µg/mL in A,B, corresponding approximately to the* IC_{50}*, and 0.15µg/mL in C,D, corresponding approximately to the* IC_{75}*). Blue lines show the number of intact cells, orange lines the number of lysed cells. Panels A,B and C,D show the data of two different microcolonies in the respective conditions. TMP was added after 30 min of growth and the experiments were stopped in each case when the microfluidic chamber became crowded with cells.*
(E) *Sample fluorescence images of the microcolony shown in D at different time points; a single cell lyses at 3.25h (white arrow) as detected by the sudden disappearance of the cytosolic fluorescent protein signal (Materials and Methods).*
