# Supplementary material for: Growth‐mediated negative feedback shapes quantitative antibiotic response
Source: Mol Syst Biol. 2022 Sep 20;18(9):e10490. doi: 10.15252/msb.202110490 (PMC9486506; doi:10.15252/msb.202110490)
Supplement: Supplementary file 9 [file MSB-18-e10490-s002.pdf]

molecular systems biology

Growth-mediated negative feedback shapes quantitative antibiotic response

S. Andreas Angermayr, Tin Pang, Guillaume Chevereau, Karin Mitosch, Martin Lercher, and Tobias Bollenbach
DOI: 10.15252/msb.202110490

Corresponding author(s): Tobias Bollenbach (t.bollenbach@uni-koeln.de)

Review Timeline:

Submission Date:	4th Jun 21
Editorial Decision:	12th Jul 21
Appeal Received:	10th Aug 21
Editorial Decision:	13th Aug 21
Revision Received:	30th May 22
Editorial Decision:	21st Jul 22
Revision Received:	19th Aug 22
Accepted:	26th Aug 22

Editor: Maria Polychronidou

Transaction Report:

(Note: With the exception of the correction of typographical or spelling errors that could be a source of ambiguity, letters and reports are not edited. Depending on transfer agreements, referee reports obtained elsewhere may or may not be included in this compilation. Referee reports are anonymous unless the Referee chooses to sign their reports.)

1st Editorial Decision 12th Jul 2021

RE: Manuscript MSB-2021-10490, Growth-mediated negative feedback shapes quantitative antibiotic response

Thank you again for submitting your work to Molecular Systems Biology. We have now heard back from two of the three referees who agreed to evaluate your study. Since their recommendations are very similar, in the interest of time we have decided to proceed with making a decision based on these two available reports. As you will see below, the reviewers raise substantial concerns on your work, which unfortunately preclude its publication in Molecular Systems Biology.

The reviewers appreciate that the goal of the study is relevant. However, they point out that as it stands the overall advance seems rather limited and several of the conclusions are not convincingly supported. Overall, the reviewers rated both the conceptual advance and the conclusiveness of the study as "Low" and they indicated that they do not support publication in Molecular Systems Biology.

Taken together and given the low level of enthusiasm expressed by the reviewers, I am afraid that we cannot offer to publish the study. I am sorry that the review of your work did not result in a more favorable outcome, and I hope that you will not be discouraged from submitting future work to Molecular Systems Biology. In any case, thank you for the opportunity to examine this work.

Reviewer #1:

Summary
This paper suggests that growth-mediated feedback loops determine the steepness, and thus the steepness of dose-response curve may reflect the antibiotic mechanism upon growth-rate. Using mathematical modeling, they have shown the shallow steepness may come from a negative growth-mediated feedback loop (and vice versa). This feedback affects the antibiotic sensitivity as well, they experimentally observed that the slower growth environment increases steepness and IC50. They also found one possible biological mechanism to answer how the negative feedback is established in the molecular level with trimethoprim (TMP), which showed the shallowest curve in their data set. TMP treatment slows the growth of bacteria. Such slow growth upregulates the target of TMP in the cell and thus decreases TMP effectivity-that is, the negative feedback. Down-regulation of TMP target with the inducible promoter attenuates the strength of the negative feedback and steepens the curve.

Overall comments
When I initially read the abstract, everything seemed to make perfect sense. However, as I read the full manuscript, I got increasingly confused in terms of what mechanistic insights the authors were aiming to convey. This confusion resulted from a collection of issues on various aspects of the paper, undercutting my confidence in the work.

At the highest level, I can see how negative feedback can attenuate dose responses. This is a concept that was established by a paper from the Balaszi lab (Nevozhay et al, PNAS 2009), which shows a negative feedback can linearize dose response in gene expression (in contrast to sharper responses in the absence of a negative feedback). This work should be cited.
The difference for the current study is that it's applied to a population response to an antibiotic. But unfortunately, this intuition was somewhat muddled by the presentation of data and analysis in the current manuscript, making me less confident as I read along. The authors have generated a substantial amount of data, a clearer and more rigorous analysis in a revision is necessary to clarify the central points. If so, the study will represent a substantial contribution to our understanding of how bacterial populations respond to drug treatment. Altogether, my reservations are on the technical (rather than conceptual) aspects of the work.

Specific issues
1. I don't see how the presented the model (eq 1 and C_eff formulation) offers any insight into their proposed mechanism. I understand how mathematically it can generate an attenuated dose response. However, it is rather odd formulation to describe the negative feedback. Specifically, their implementation of the negative feedback assumes that C_eff increases linearly with growth rate, beyond the external concentration (C_ext). This assumption doesn't make sense to me and there is no explanation how such a dependence would arise through some biologically plausible mechanisms. It's also disconnected with their experimental interrogation (e.g. the analysis on the role of antibiotic target). If this is the mechanism they propose, experiments should be designed to demonstrate this dependence and modulate it.

Considering the extensive quantitative data the authors have generated, the authors have a real opportunity to develop a more biologically relevant model that better integrates with experimental analysis. If so, the paper will be substantially strengthened.

2. I wish to see more details on how growth rates were calculated from the growth curves. Since growth rates are used throughout the manuscript as the central metric, it is particularly important to ensure that the calculations are well justified. For example, from the textual description, the authors noted that some growth rates were determined by cells producing luminescence. It's unclear which sets of growth rates were estimated by this approach. A major caveat of the luminescence readout is that it is highly sensitive to the physiological state of the cell population. It is unclear if these measurements were calibrated with growth rates derived from OD measurements.

3. One major aspect causing the confusion is the following. The population size is dictated by a growth term and a death term. When applying an antibiotic, it can suppress the growth term and promote the death term. When discussing growth rate suppression by TMP mechanistically, I assume the authors refer to the suppression of the growth term. As a result of this suppression, the death term can also be suppressed. However, the effects of TMP were largely evaluated based on the combined population response (combining both the growth and death terms). This lumping is reflected in the description of Figure 1A and also in the formulation of their simple model. I think that separating the effects of TMP on the growth term and those on the death term is important for developing a coherent interpretation of their data.

4. The authors tried different ways to change the growth rates and found that reducing growth by lowering the temperature did not change the antibiotic sensitivity. They then went on to say that ... " this observation indicates that the observed effect has biological - rather than an elementary physical-origin". This statement and the previous statement seem questionable. Changing temperature should cause quite significant changes to the cell physiology in general.

Also, is their interpretation here consistent with the analysis on target regulation by growth? That is, do they expect that the expression of drug target does not change when temperature is shifted?

5. A major point of the paper is that the inhibition by antibiotic TMP upregulates FolA, which reduces efficacy of TMP (again, this proposed mechanism doesn't connect to their proposed model). It's unclear whether this is specific to TMP or applicable to other antibiotics. Also, does this require some sort of molecular mechanisms or is it just a consequence of slower growth?

6. In figure 5, the definition of down-regulation was misleading to me, since the actual folA hyper-expressed over TMP concentration than the WT drug-free expression level. This may lead to the discrepancy of IC50 predictions from model and measured IC50s. The model supports that the increase of alpha decreases IC50. However, figure 5B seemed like increasing alpha (constant and down-regulation) increased the IC50, which indeed come from overexpression of folA.

7. If I read correctly, reference growth rate to normalize whole dataset in figure 2 is the left and bottom most block from where the drug-free and no growth-intervention condition. The ICs that the model can compare were the normalized response to only drug-free condition, where g0 is the constant variable. Thus, IC90 increase and fold comparisons across different g0 in figure 2 (B-C, E-F, H-I) may not be the valid way. Also, IC90 comparison may show different pattern from IC50 comparison due to the hill equation characteristic itself. This case is being seen in figure 3C, when the curves were normalized to corresponding g0s, the slower growth indeed increased IC50 but decreased IC90.

Reviewer #3:

This paper seeks to explain the contribution of antibiotic-mediated growth inhibition on observed dose-response relationships. The authors show that a shallow dose-response curve is based on a negative feedback loop of antibiotic-mediated growth inhibition, using the antibiotic trimethoprim. Overall, explaining the shape of a dose-response is an inherently open and interesting question, and thus the overall idea of the paper is very exciting. However, there's very little novel in this manuscript; their main argument, that slower growth leads to decreased antibiotic efficacy is well-established. Moreover, their model formulation, which purports to explain Hill-like behavior, already assumes a Hill coefficient; it's in effect no different than the typical dose-response equation, and therefore doesn't provide any additional mechanistic information. Finally, the data presented in many cases did not seem to support the conclusions made. As such, I do not believe this paper to be suitable for MSB.

Major points:

1. The paper presents quite a general mechanism, e.g., that TMP inhibits growth, and slower growth leads to shallower DR curve. The proposed mechanism does not involve TMP-specific factors, and yet the data shown seems highly specific to TMP. Conceptually, it's unclear why TMP is singled out compared to the other antibiotics, when the mechanism remains so general - if it is simply that TMP inhibits growth, then this should be true for other antibiotics that inhibit growth via a similar negative feedback (eg chloramphenicol, tet, etc.). Yet, there are many cases where static drugs show far less shallow dose responses compared to cidal, which would have less of an inibitory effect, e.g., see Fig 2 of Tan et al MSB 2012 where Cm dose response is much sharper than Kan. Overall, this suggests a TMP-specific mechanism, and as discussed below, the generality claims made seem widely unfounded.

2. I have several issues with the model formulation: First, there is no justification for the equation chosen for Ceff (Line 130-132), and it seems to be not even the simplest way to capture what the authors want it to capture. E.g. why not just Ceff = Cext * [1 + alpha*g/g0]? Instead, the authors are saying Ceff = Cext * [1 + alpha - alpha*g/g0]. What is the justification or purpose of that extra alpha term? By definition, as written, alpha>0 will potentiate the effect of the Hill coefficient, and vice versa. Moreover, and more to the point, the authors argue that the antibiotic effect on growth rate accounts for the steepness of the DR (i.e., the effective hill coefficient). If so, their alternative formulation of growth rate as a function of [abx] should not contain this Hill structure. If they had presented some biologically justified alternate formulation that did not rely on Hill equation, yet still displayed Hill-like dose response, that would be interesting and supportive - they could then argue that feedback etc is leading to Hill phenotype.
3. The data in Fig. 1C certainly shows some amount of correlation, but the methods aren't clear to me. 78 strains were chosen arbitrarily - which strains were used? The authors claim that these strains are unrelated to the mechanism of drug action, yet TMP interferes in folate metabolism which both feeds into, and from, various other interrelated aspects of cell physiology that are likely impacted by many genes. Also, why was 30% of the reference strain chosen and not the IC50 concentration? It seems quite arbitrary.
4. Data in supplementary Figure 2 is perhaps the least visually compelling of any trend between IC50 and growth rate. First, there is no obvious relationship for TMP data compared to the other drugs presented; also that TMP is statistically significant when CHL is not, despite the two having similarly low correlations, seems odd. Even so, I am not convinced that the statistical significance, in this case, is biologically meaningful.
5. The key data in this paper is presented in Fig 2b; however, at aMG/Glu >= 10 it looks like the growth rates are not even monotonically decreasing with increasing TMP, which does not necessarily suggest to me that the dose responses are shallower. The authors would likely need a denser range of Amg/Glu ratios on the range [0 5]. Also, the heatmap itself makes it quite challenging to interpret the trends - the authors should plot multiple growth vs TMP dose response lines (instead of this heatmap).
6. Lines 228-232, - that modulating growth by temperature does not result in the same phenotype raises further questions about the central claim. It makes sense that the non-normalized growth rates decrease with decreasing temp (Fig S8 LHS), but the fact that the normalized curves (RHS) overlap counteracts their premise that lower growth == shallower curves (eg Discussion lines 347-348). In fact, it suggests a more complex explanation (perhaps circling back to metabolism as of TMP as I suspect).
7. A great deal of relevant literature was not referenced, including Lee et al in PNAS 2018 and Lopatkin et al in Nature Microbiology 2019. Both cases investigate growth specific quantitative relationships of antibiotic and susceptibility.
8. The data presented overall lacked transparency - most figures reported normalized values on both axes. For example, why did the x-axis need to be normalized in Fig. 1C? Also, the x-axis was scaled in fig. 1A that made it difficult to interpret the trends. At the minimum, in all cases, the raw data should be presented in the supplementary information.
9. The authors argue that "TMP lowers growth, which in turn weakens the inhibitory effect of the drug". If this were the case, wouldn't you expect growth rate to oscillate with time -- TMP lowers growth -> less potent TMP == faster growth -> faster growth == more potent TMP.
10. Figure 5 lacks sufficient controls. In particular, they don't account for the increased burden of IPTG expression on growth rate in the absence of drug, which could also influence antibiotic susceptibility. Also lines 171-173, the sentence starting with "Notably..." is not supported by their own data.

** As a service to authors, EMBO Press offers the possibility to directly transfer declined manuscripts to another EMBO Press title or to the open access journal Life Science Alliance launched in partnership between EMBO Press, Rockefeller University Press and Cold Spring Harbor Laboratory Press. The full manuscript and if applicable, reviewers' reports, are automatically sent to the receiving journal to allow for fast handling and a prompt decision on your manuscript. For more details of this service, and to transfer your manuscript please click on Link Not Available. **

Dear Dr. Polychronidou,

Thank you for your evaluation of our manuscript and sorry for this delayed response. We understand and respect your decision based on these reviewer reports. It seems that we have presented the results of this work in a way that has led to several misunderstandings. This is unfortunate as the manuscript was informally reviewed by several experienced systems biologists from different fields prior to submission, who had no such problems. It has become clear that the presentation of this work needs to be improved to be accessible to a broader audience.

However, we cannot let these reports stand without comment. First, please note that reviewer 1 explicitly states that, upon revision, this study will represent a substantial contribution to our understanding of how bacterial populations respond to drug treatment. Their confidence in our results seems to be affected by a number of technical concerns, which we are quite confident can all be easily addressed with additional controls and further explanation.

Reviewer 3 agrees that explaining the shape of a dose-response is an inherently open and interesting question, and thus the overall idea of the paper is very exciting and then voices various major criticisms. However, most of these criticisms do not hold up to scrutiny and some indicate a disturbing lack of care and attention. Here is a small selection of the most glaring examples:

- Major point 1 completely ignores Fig. 4 and the accompanying part of the main text (lines 255-298), which actually explains the TMP-specific mechanism.
- Major point 3 criticizes that it is not clear how 78 strains were chosen in Fig. 1C. However, Fig. 1C shows genome-wide data (~4,000 strains), which is explained in the figure legend and is easily seen from the number of data points in the figure alone.
- Major point 7 criticizes that relevant literature was not cited and specifically mentions Lee et al in PNAS 2018. We were stunned by this since we did cite this paper (see reference list and line 89).
- The reviewer argues that ...their main argument, that slower growth leads to decreased antibiotic efficacy is well-established. This is established for the killing rate of beta-lactam antibiotics, but not for other antibiotics (lines 87-89 in the Introduction). The reviewer provides no references for their far more general claim. Notably, the data in our manuscript do not even support this supposedly "well-established" phenomenon for the IC50 of most antibiotics (see e.g. Fig. 2C,F,I).

There are more issues of a similar insight value, which would require going deeper into details (we will be happy to explain those as well if it is helpful). A few of the points raised by reviewer 3 point at more plausible misunderstandings and issues that could be easily addressed. Still, overall, the quality of this report is unacceptable.

It is clear that the manuscript needs to be improved to avoid any misunderstandings and the reports will be helpful to do so. We are confident that we can clarify all technical concerns and, in particular, that we can improve the model and its connection to specific mechanisms. Given the blatant incoherence of one of the two reports that the editorial decision is based on and the fact that reviewer 1 suggests a revision in which their technical concerns should be addressed, we would like to ask if you would consider a strongly revised version of this manuscript with an improved model and presentation as a new submission. Please let us know if that is possible.

Thank you and best wishes,

Tobias Bollenbach

Manuscript Number: MSB-2021-10490R-Q

Thank you for your message related to our decision on your manuscript MSB-2021-10490. I apologize for the delay in getting back to you, which was due to the high number of submissions that we have been receiving. I have now had the chance to consider the manuscript and the points raised in your appeal letter and I have discussed your request with the team. As I will explain below, we would not be opposed to considering a new submission addressing the issues raised by the reviewers.

During the review, the two reviewers appreciated that examining the effect of negative feedback on the response of bacterial populations to antibiotic treatment is a relevant topic. However, they both questioned the conclusiveness of the study and they were not convinced that as it stands the resulting advance seems sufficient to warrant publication in Molecular Systems Biology. Both of them ranked the conceptual novelty as "Medium/Low" and the validity of the main conclusions as "Low". I wanted to clarify that the decision to not proceed with the publication of the manuscript was not solely based on the comments of reviewer #3, but if fact also largely based on the rather substantial concerns of reviewer #1. While indeed reviewer #1 mentioned that a revision could result in a study suitable for publication, they also raised several concerns, the most significant of which are summarized below:

- Reviewer #1 mentioned that the model seems somewhat disconnected from the rest of the analyses and it is not sufficiently integrated with the experimental data to derive mechanistic insights. They also expressed some concerns regarding the model's biological relevance.
- Reviewer #1 questioned the generality of the proposed mechanism for other antibiotics beyond TMP.
- They also listed several technical concerns, including the calculation of growth rates, potential issues due to jointly considering the effect of the drug on growth rate and death and the analyses modifying the growth rate.

Some of these concerns, including technical ones are also shared by reviewer #3. Overall, we feel that these concerns are rather substantial as they potentially compromise the validity and significance of the presented conclusions.

We appreciate that in your preliminary response you clarify some points related to the report of reviewer #3, which seem to have been due to potential misunderstandings. Nevertheless, we think that beyond improving the presentation of the study and providing further clarifications, significant additional analyses would be required in order to address the more substantial issues raised, including those summarised above. Addressing these issues would be important for the eventual acceptance of the study. Considering that the reviewers did have positive words for the topic and the goals of the study, we would not be opposed to considering a revised and extended study as a new submission, provided that these concerns have been satisfactorily addressed.

Cologne, May 30, 2022

Dear Dr. Polychronidou,

Attached please find the revised version of our manuscript titled "Growth-mediated negative feedback shapes quantitative antibiotic response", which was reviewed by your journal last year. Thank you for your editorial guidance and your willingness to consider a strongly revised version of this manuscript. We also thank the reviewers for the careful evaluation of our work and for the constructive feedback on our manuscript. We are pleased that both reviewers found our study interesting, recognized its potential, and made valuable suggestions for improvements. Their constructive criticism enabled us to corroborate our results in a targeted manner.

Compared to our initial submission, we have now included a drastically improved mathematical model that is better connected to the experimental evidence and provides more mechanistic insight. We developed this model in collaboration with Dr. Tin Pang and Prof. Martin Lercher (University of Düsseldorf), who are experts in models of metabolism and cellular resource allocation; therefore, they were added as co-authors. We have also heavily revised the presentation of our results to avoid a number of misconceptions that became clear to us from the reviewers' comments. Finally, as requested by the reviewers, we added several additional experimental and data analysis controls, in particular to address technical concerns regarding quantification of growth rates and the potential role of cell death. For the last point, we directly observed cell survival under trimethoprim using time-lapse imaging of single cells in a microfluidic device; these experiments were performed by Dr. Karin Mitosch (EMBL), who was added as a co-author accordingly.

These revisions have considerably improved the manuscript overall. Below, please find our point-by-point responses to the reviewers.

We are looking forward to hearing from you.

Best regards,

T. Bollenbach

Tobias Bollenbach (on behalf of all authors)

Point-by-point response

Reviewer #1:

Summary

This paper suggests that growth-mediated feedback loops determine the steepness, and thus the steepness of dose-response curve may reflect the antibiotic mechanism upon growth-rate. Using mathematical modeling, they have shown the shallow steepness may come from a negative growth-mediated feedback loop (and vice versa). This feedback affects the antibiotic sensitivity as well, they experimentally observed that the slower growth environment increases steepness and IC50. They also found one possible biological mechanism to answer how the negative feedback is established in the molecular level with trimethoprim (TMP), which showed the shallowest curve in their data set. TMP treatment slows the growth of bacteria. Such slow growth upregulates the target of TMP in the cell and thus decreases TMP effectivity-that is, the negative feedback. Down-regulation of TMP target with the inducible promoter attenuates the strength of the negative feedback and steepens the curve.

Overall comments

When I initially read the abstract, everything seemed to make perfect sense. However, as I read the full manuscript, I got increasingly confused in terms of what mechanistic insights the authors were aiming to convey. This confusion resulted from a collection of issues on various aspects of the paper, undercutting my confidence in the work.

At the highest level, I can see how negative feedback can attenuate dose responses. This is a concept that was established by a paper from the Balaszi lab (Nevozhay et al, PNAS 2009), which shows a negative feedback can linearize dose response in gene expression (in contrast to sharper responses in the absence of a negative feedback). This work should be cited.

We agree that (Nevozhay et al, PNAS 2009) is relevant in the context of our work and have added a citation to this work in the Introduction (line 41).

The difference for the current study is that it's applied to a population response to an antibiotic. But unfortunately, this intuition was somewhat muddled by the presentation of data and analysis in the current manuscript, making me less confident as I read along. The authors have generated a substantial amount of data, a clearer and more rigorous analysis in a revision is necessary to clarify the central points. If so, the study will represent a substantial contribution to our understanding of how bacterial populations respond to drug treatment. Altogether, my reservations are on the technical (rather than conceptual) aspects of the work.

We thank the reviewer for their careful evaluation of our work and their recognition of the significant contribution this work can make to our understanding of bacterial responses to antibiotic treatment. It has become clear that the presentation of our results needs to be improved. To this end, we have thoroughly revised the entire manuscript and addressed all specific issues raised by the reviewer, especially the technical issues (see below).

Specific issues

1. I don't see how the presented the model (eq 1 and C_eff formulation) offers any insight into their proposed mechanism. I understand how mathematically it can generate an attenuated dose response. However, it is rather odd formulation to describe the negative feedback. Specifically, their implementation of the negative feedback assumes that C_eff increases linearly with growth rate, beyond the external concentration (C_ext). This assumption doesn't make sense to me and there is no explanation

how such a dependence would arise through some biologically plausible mechanisms. It's also disconnected with their experimental interrogation (e.g. the analysis on the role of antibiotic target). If this is the mechanism they propose, experiments should be designed to demonstrate this dependence and modulate it.

Considering the extensive quantitative data the authors have generated, the authors have a real opportunity to develop a more biologically relevant model that better integrates with experimental analysis. If so, the paper will be substantially strengthened.

We thank the reviewer for pointing out this key issue. We agree that the minimal model we had used in our original submission did not connect well to the experimental data. We had mainly included this generic model to illustrate the general effects of growth-mediated feedback loops on the shape of dose-response curves. Following the suggestion of the reviewer, we have now developed a biologically more relevant model that is specific to the mode of action of trimethoprim and explicitly captures the regulation of its target enzyme FolA. We developed this model using the framework of Constrained Allocation Flux Balance Analysis (CAFBA) (Mori et al, 2016). This was done in collaboration with Dr. Tin Pang and Prof. Martin Lercher (University of Dusseldorf) who are experts for models of metabolism and cellular resource allocation; they were consequently added as co-authors in the revised manuscript.

In brief, the model describes the combined effects of carbon limitation and trimethoprim on bacterial growth. It captures how the interaction of trimethoprim with its target enzyme FolA reduces the metabolic flux through the folic acid synthesis pathway, thereby reducing the growth rate. A key assumption of the model is that growth is limited by the metabolic flux catalyzed by FolA when sufficiently many FolA enzymes are blocked by trimethoprim. Using the experimentally measured regulation of FolA protein levels in response to trimethoprim and glucose limitation (Fig. 4) as an input to this model yields good agreement with the experimentally observed dose-response curve shapes at different levels of carbon limitation. In particular, it produces a shallow dose-response curve with dose-sensitivity slightly above 1 for the wild type without carbon limitation, in reasonable quantitative agreement with experimental observations (Fig. 1A); with increasing carbon limitation, the dose-response curves predicted by the model become steeper as observed experimentally (Fig. 3). Further, moderate glucose limitation can slightly increase the absolute growth rate under trimethoprim, again as observed experimentally (Fig. 2B). In the model, we further mimicked the experiment in which we break the wild-type regulatory loop and control the expression of *folA* with an external inducer (Fig. 5); in this situation, the model produces the experimentally observed steepening of the dose-response curve when FolA level is forced to be constant or its regulation is inverted compared to the wild type. Overall, the model relies on biologically plausible assumptions and provides a semi-quantitative mechanistic explanation for the experimentally observed phenomena. It thus corroborates that the negative feedback loop underlying the exceptionally shallow trimethoprim dose-response curve in wild type is mediated by the growth-rate-dependent regulation of the drug target.

We have extensively revised the manuscript to integrate this new model. Specifically, we have added a new section at the end of the results part (lines 398-426) to explain the essence of the model and the new Fig. 6 and Supplementary Fig. 12 to show the key results that can be directly compared to the experimental data. We have further added a new section *Cellular resource allocation model* to the Methods part to explain the technical details of this model (lines 761-841). We have drastically shortened the description of the generic minimal model of growth-mediated feedback in antibiotic responses in the main text (lines 138-140) and largely shifted it to the Methods (lines 740-759). We could remove this minimal model completely, but would prefer to keep it in this minor role since it can help to illustrate the general effects of growth-mediated feedback loops (Fig. 1B), irrespective of the underlying molecular mechanism.

2. I wish to see more details on how growth rates were calculated from the growth curves. Since growth rates are used throughout the manuscript as the central metric, it is particularly important to ensure that the calculations are well justified. For example, from the textual description, the authors noted that some growth rates were determined by cells producing luminescence. It's unclear which sets of growth rates were estimated by this approach. A major caveat of the luminescence readout is that it is highly sensitive to the physiological state of the cell population. It is unclear if these measurements were calibrated with growth rates derived from OD measurements.

We agree that it is important to make the quantification of growth rates from the growth curves transparent. In brief, we performed a linear a fit to the background-subtracted log(OD) (or luminescence) measurements in the OD window corresponding to exponential phase. This is described in the Methods section (lines 569-585). We have revised Supplementary Fig. 11, which shows examples of typical growth curves, and now illustrate how growth rates were calculated in this figure (new inset in panel B).

We used the luminescence-based approach for measuring growth rates only for the experiment in which temperature was varied (Supplementary Fig. 8); growth rates in all other experiments were measured using OD. It is correct that the luminescence intensity per cell is highly sensitive to the physiological state of the cell but, importantly, the rate of exponential increase in luminescence in steady-state exponential growth is independent of this. A major advantage of using luminescence is that the growth curve can be followed over ~5 orders of magnitude (~16-17 doublings) starting from just ~100 cells, enabling more precise quantification of growth rates than possible from OD measurements. This technique is established (Kishony and Leibler, Journal of Biology, 2003); we recently calibrated it and validated that it results in the same growth rates as obtained from OD measurements (see Supplementary Fig. 7e in Kavcic *et al.*, Nat Comm, 2020). We have revised the relevant part of the Methods section (*Growth conditions and growth rate measurements*, in particular lines 521-522 and lines 561-563) to clarify these points and explained which sets of growth rates were measured using OD or luminescence, respectively.

3. One major aspect causing the confusion is the following. The population size is dictated by a growth term and a death term. When applying an antibiotic, it can suppress the growth term and promote the death term. When discussing growth rate suppression by TMP mechanistically, I assume the authors refer to the suppression of the growth term. As a result of this suppression, the death term can also be suppressed. However, the effects of TMP were largely evaluated based on the combined population response (combining both the growth and death terms). This lumping is reflected in the description of Figure 1A and also in the formulation of their simple model. I think that separating the effects of TMP on the growth term and those on the death term is important for developing a coherent interpretation of their data.

This is an important point and we have added new control experiments to address it. Please note that it is established that trimethoprim can kill bacteria by causing thymineless death, but this requires certain nutrient conditions – in minimal media, this drug merely results in growth inhibition and cell stasis, even at higher concentrations (see e.g. Kwon *et al.*, ACS Chemical Biology, 2010). Still, to validate that cell death is negligible in our experiments even in rich growth medium, we performed time-lapse imaging of *E. coli* in a microfluidic device at the relevant concentrations of TMP at which growth observed at the population level is reduced but not fully inhibited. We observed virtually no cell death in our experimental conditions during the relevant time period. We also imaged cells taken from populations cultured on 96-well plates exactly as those reported in the main figures and, again, did not observe any dead cells. With both approaches we found that cells elongate at sub-MIC concentrations of trimethoprim, confirming previous observations (see e.g. Bollenbach *et al.*, Cell, 2009 and references therein). However, these observations indicate that cell death, if it occurs at all, requires higher concentrations (above the

MIC) of this antibiotic under the growth conditions we used. Thus, this should alleviate the reviewer's concerns.

We now explain this point in the main text (lines 202-207) and also added a reference to (Kwon *et al.*, 2010) (line 204) to clarify that TMP can only kill bacteria under special circumstances. We have further added the new Supplementary Fig. 16, which shows the results for cell survival under trimethoprim in the microfluidics experiment; this experiment is explained further in the Methods (lines 719-738). Dr. Karin Mitosch (EMBL) performed the time-lapse imaging experiments and was added as a co-author accordingly.

4. The authors tried different ways to change the growth rates and found that reducing growth by lowering the temperature did not change the antibiotic sensitivity. They then went on to say that ... " this observation indicates that the observed effect has biological - rather than an elementary physical-origin". This statement and the previous statement seem questionable. Changing temperature should cause quite significant changes to the cell physiology in general.

Also, is their interpretation here consistent with the analysis on target regulation by growth? That is, do they expect that the expression of drug target does not change when temperature is shifted?

We agree that this point was not explained in sufficient detail and thank the reviewer (and reviewer 3) for pointing it out. In the relevant paragraph, we refer to the classical results on the effects of temperature on bacterial cell physiology (reviewed in Bremer & Dennis, 2008, section *Macromolecular Composition during Growth at Different Temperatures*). In brief, when the steady-state exponential growth rate is altered by changing the temperature rather than the nutrient content of the growth medium, the concentrations of the relevant macromolecules in the cell are known to remain constant to a good approximation. Specifically, the total amounts of protein, RNA, and RNA polymerase per cell mass, the fractions of total RNA synthesis corresponding to stable RNA and mRNA synthesis, and the DNA replication fork patterns all remain constant when growth rates are altered by a change in temperature, at least in the relevant temperature range of 25 °C to 38 °C (Bremer & Dennis, 2008). The reason for this behavior is that the corresponding chain elongation rates (for DNA, RNA, and polypeptides) all depend approximately equally on temperature (Bremer & Dennis, 2008). Therefore, the most plausible null expectation for the effects of temperature changes is that they have no (or very limited) effects on molecular composition and cell physiology. This expectation agrees well with our observations for the dose-response curve measured at different temperatures, which is all we wanted to say on this point.

We now explain this point in more detail to make it accessible to a broader audience and clarify the conclusion we draw from it. We have revised and expanded the relevant paragraph accordingly (lines 262-276). Note that this result is relatively minor; we have included it only because we felt that some readers might wonder about the effects of temperature. If it is still confusing, we could remove this paragraph and the corresponding supplementary figure entirely – it is not essential to the main results of this work, but merely confirms an expectation that many experts would have.

5. A major point of the paper is that the inhibition by antibiotic TMP upregulates FolA, which reduces efficacy of TMP (again, this proposed mechanism doesn't connect to their proposed model). It's unclear whether this is specific to TMP or applicable to other antibiotics. Also, does this require some sort of molecular mechanisms or is it just a consequence of slower growth?

These points are important to clarify as pointed out by both reviewers. Indeed, we now understand that it was not sufficiently clear which aspects of our work are specific to trimethoprim and which apply more generally to antibiotics and other drugs.

The upregulation of *folA* by TMP is specific to this antibiotic, as it depends on the specific drug target. This is now explicitly accounted for in the new resource allocation model we added in the revision (see also reply to point 1 above), which should help clarify this drug-specific mechanism. However, a similar scenario (upregulation of drug target in response to drug) applies to some ribosome inhibitors, suggesting that this is a more general type of negative feedback in drug responses. It is certainly interesting to explore these mechanisms in more detail for other drugs, but this goes beyond the scope of our manuscript.

Our results indicate that no specific molecular mechanism is required for the observed *folA* upregulation (e.g. via a specific transcription factor that senses TMP and controls the *folA* promoter), but that this upregulation largely occurs as a consequence of slower growth alone. Of particular note, this upregulation occurs even in the absence of TMP when the growth rate is lowered by carbon limitation (Fig. 4B). The FolA level essentially follows a similar trend as the change in protein concentration expected from bacterial growth laws for genes without specific regulation (*cf.* Fig. 2C in Scott *et al.*, Science, 2010).

In addition to including the new theoretical model that captures this mechanism, we have revised the parts of the main text where these points are explained (lines 311-313, 317-319, and 328-330) to make them clearer. We have also revised the legend of Fig. 4 to highlight these points. We have further expanded the part of the Discussion where we explain the extent to which our results are applicable to other antibiotics than TMP (lines 446-463).

6. In figure 5, the definition of down-regulation was misleading to me, since the actual folA hyper-expressed over TMP concentration than the WT drug-free expression level. This may lead to the discrepancy of IC50 predictions from model and measured IC50s. The model supports that the increase of alpha decreases IC50. However, figure 5B seemed like increasing alpha (constant and down-regulation) increased the IC50, which indeed come from overexpression of folA.

This is a good point and we thank the reviewer for pointing it out. It is correct that in Fig. 5 we need to start from a higher *folA* expression level to analyze the effect of FolA levels that decrease with TMP concentration, because the wild-type expression level is only slightly above the detection limit of our technique. Therefore, we cannot quantitatively control this expression level below the wild-type level using the approach shown in Fig. 5. However, the purpose of this experiment was precisely to invert the wild-type response (which goes from a low FolA concentration in the absence of TMP to a high FolA concentration when the TMP concentration is increased) to the opposite response (i.e. high FolA concentration in the absence of TMP, decreasing with increasing TMP concentration). Despite the limited accessible dynamic range of FolA levels, the effect of inverting the wild-type regulation on the shape of the dose-response curve can be studied in this way. However, we agree that the term *down-regulation* is not ideal for this purpose and may lead to misunderstandings. To avoid any confusion, we now refer to the modified *folA* regulation as *inverted regulation* instead of *down-regulation* throughout, since it exactly inverts the wild-type regulation.

The reviewer is also correct that *folA* is slightly overexpressed in this assay, even in the constant FolA condition (albeit less than two-fold). This is due to the use of the Llac-O1 promoter (in combination with a lac repressor) for which we approximately get the wild-type expression level (of the *folA* promoter; see Methods, lines 657-664). Indeed, this leads to an increase in IC_{50} compared to the wild type, which was not well captured by the previous model. The new resource allocation model we have added in the revised version of the manuscript correctly captures this shift in IC_{50} when the slight overexpression of *folA* is taken into account (new Fig. 6B).

We have revised the corresponding part of the main text (lines 374-383) and Fig. 5 and its legend to clarify this point. The comparison of the new model to these data is now explained in lines 421-426.

7. If I read correctly, reference growth rate to normalize whole dataset in figure 2 is the left and bottom most block from where the drug-free and no growth-intervention condition. The ICs that the model can compare were the normalized response to only drug-free condition, where g0 is the constant variable. Thus, IC90 increase and fold comparisons across different g0 in figure 2 (B-C, E-F, H-I) may not be the valid way. Also, IC90 comparison may show different pattern from IC50 comparison due to the hill equation characteristic itself. This case is being seen in figure 3C, when the curves were normalized to corresponding g0s, the slower growth indeed increased IC50 but decreased IC90.

It is correct that the values of the IC_{50} and IC_{90} depend on the condition used for normalization. In Fig. 2 (panels B, E, H), the entire dataset was normalized to the condition on the bottom left, respectively, corresponding to the highest growth rate in the absence of TMP. We used this normalization because it represents the most stringent criterion for the protective effect of slower growth: An increase in IC_{90} here means that the same absolute growth rate was observed at a higher TMP concentration than in the absence of glucose limitation (or other perturbations). Nevertheless, following the suggestion of the reviewer, we reanalyzed these data by normalizing each column in Fig. 2B,E,H to the growth rate for zero TMP in that column. The resulting trend of increasing TMP resistance with decreasing growth rate, which is the main point of Fig. 2, remains unchanged in this alternative analysis. As indicated by the reviewer, it makes more sense to investigate the IC_{50} for this normalization. For the IC_{50}, the same trend for TMP is seen as for the IC_{90} in Fig. 2.

This analysis is shown in the new Supplementary Fig. 15 and mentioned in the main text in lines 232-235 and in the legend of Fig. 2. We have also revised Fig. 2 to make it clearer, in part at the suggestion of reviewer 3.

It should be noted that analyzing the IC_{50} for the normalization used in Fig. 2 is less useful because growth inhibition of 50% or more is already achieved in many conditions in the absence of antibiotic (due to glucose limitation alone), i.e. the IC_{50} defined in this way would be zero or negative in many cases, which is not a meaningful measure.

Reviewer #3:

This paper seeks to explain the contribution of antibiotic-mediated growth inhibition on observed dose-response relationships. The authors show that a shallow dose-response curve is based on a negative feedback loop of antibiotic-mediated growth inhibition, using the antibiotic trimethoprim. Overall, explaining the shape of a dose-response is an inherently open and interesting question, and thus the overall idea of the paper is very exciting.

We thank for reviewer for their efforts and for their appreciation of the overall idea of our paper.

However, there's very little novel in this manuscript; their main argument, that slower growth leads to decreased antibiotic efficacy is well-established.

We agree that this is well-established for the killing rate of β-lactam antibiotics and for extreme cases such as persisters (lines 87-89 in the Introduction of the original manuscript; lines 101-104 in the revised version). However, many antibiotics do not kill bacteria, and we are not aware of any results demonstrating a more general correlation between growth rate and common measures of antibiotic efficacy (such as MIC or IC_{90}) that would generalize this trend across drug classes for both bacteriostatic

and bactericidal antibiotics. On the contrary, it has been shown in (Greulich *et al.*, MSB, 2015) that the IC_{50} changes in opposite ways with increasing drug-free growth for different ribosome inhibitors: it decreases for tetracycline and chloramphenicol, but increases for streptomycin and kanamycin (see Fig. 2 in Greulich *et al.*). If there are additional relevant references on this point, we would be happy to include them as context for our work.

A major objective of our study was to systematically determine how the efficacy of different antibiotics changes with the drug-free growth rate. Importantly, our data do not support the scenario that slower growth always decreases efficacy: the IC_{90} of most antibiotics does not increase with decreasing drug-free growth rate (see Fig. 2 and Supplementary Figures 5 and 6). The β-lactam mecillinam and trimethoprim were the only antibiotics that showed a clearly detectable increase in IC_{90} when the drug-free growth rate was reduced; for mecillinam, this increase occurred only when growth was reduced by overexpressing a gratuitous protein (Supplementary Fig. 6). Notably, trimethoprim is the only antibiotic that showed a general increase in IC_{90} when drug-free growth rate was reduced, regardless of whether this was done by genetic perturbations (Fig. 1C), glucose limitation, gratuitous protein overexpression, or by changing the carbon source (Fig. 2).

We have revised the relevant parts of the Introduction (lines 104-111) and the part of the main text where we explain these results (lines 165-248) to clarify this point.

Moreover, their model formulation, which purports to explain Hill-like behavior, already assumes a Hill coefficient; it's in effect no different than the typical dose-response equation, and therefore doesn't provide any additional mechanistic information.

We thank the reviewer for pointing out this key issue, which is closely related to the first point of reviewer 1. We agree that the minimal model we had used in our original submission did not provide additional mechanistic information. We had mainly included this generic model to illustrate the general effects growth-mediated feedback loops on the shape of dose-response curves. Following the suggestion of the reviewer, we have now developed a more mechanistic model specific to the mode of action of trimethoprim that explicitly captures the function and regulation of its target enzyme FolA. We developed this model using the framework of Constrained Allocation Flux Balance Analysis (CAFBA) (Mori *et al.*, 2016). Importantly, this model produces a shallow dose-response curve that can be approximated by a Hill function with dose-sensitivity (Hill coefficient) slightly above 1 for the wild type without carbon limitation (cyan line in new Fig. 6A), which is in reasonable quantitative agreement with experimental observations (Fig. 1A); with increasing carbon limitation, the dose-response curves predicted by the model become steeper as observed experimentally (Fig. 3).

We have added a new section at the end of the results section (lines 398-426) to explain the essence of the model, and the new Fig. 6 and Supplementary Fig. 12 to show its key results. The details of the model are now explained in the Methods (lines 761-841). For more details on this model, see also our response to the first point of reviewer 1 above.

Finally, the data presented in many cases did not seem to support the conclusions made. As such, I do not believe this paper to be suitable for MSB.

Below, we address the specific points raised by the reviewer to highlight how the data presented support our conclusions.

Major points:
1. The paper presents quite a general mechanism, e.g., that TMP inhibits growth, and slower growth leads to shallower DR curve. The proposed mechanism does not involve TMP-specific factors, and yet the data

shown seems highly specific to TMP. Conceptually, it's unclear why TMP is singled out compared to the other antibiotics, when the mechanism remains so general - if it is simply that TMP inhibits growth, then this should be true for other antibiotics that inhibit growth via a similar negative feedback (eg chloramphenicol, tet, etc.). Yet, there are many cases where static drugs show far less shallow dose responses compared to cidal, which would have less of an inibitory effect, e.g., see Fig 2 of Tan et al MSB 2012 where Cm dose response is much sharper than Kan. Overall, this suggests a TMP-specific mechanism, and as discussed below, the generality claims made seem widely unfounded.

There seems to be a misunderstanding here that needs to be clarified. Our proposed mechanism is specific to TMP and involves TMP-specific factors, namely FolA, the target of TMP, and its regulation (Fig. 4). A key finding is that this target is upregulated in response to the reduction in growth rate alone. Since FolA is targeted by TMP and not by any of the other antibiotics investigated, this mechanism is specific to TMP. TMP was singled out because it has by far the shallowest dose-response curve of all antibiotics studied (Fig. 1A), suggesting that a growth-mediated negative feedback loop may be at play (Fig. 1B); furthermore, the response to TMP in genome-wide gene deletion strains is strongly anti-correlated with their drug-free growth rate (Fig. 1C,D). The latter point is crucial: all antibiotics can inhibit growth, but we expect that growth-mediated negative feedback affects the response to antibiotics only if slower drug-free growth attenuates the inhibitory effect of the drug. That is, we by no means claim that this feedback loop applies generally to all antibiotics. We have revised the relevant parts of the main text (lines 108-111, 135-136, 146-148, 164-165, 186-191) and the legends of Fig. 1 and Fig. 2 to clarify this point.

While this is not the focus of our work, a similar scenario as for trimethoprim probably applies to the ribosome inhibitors mentioned by the reviewer (chloramphenicol, tetracycline). Here, the target (the ribosome) is also upregulated in response to the drug. Although the effect is not as extreme as with trimethoprim, chloramphenicol and tetracycline have among the shallowest dose-response curves in our dataset (Fig. 1A), suggesting that the regulation of the respective drug target plays a more general role in shaping dose-response curves. Certainly, other factors specific for each drug may contribute to the shape of the dose-response curve. We conclude only that the presence of a strong growth-mediated feedback loop, as we demonstrate for trimethoprim, has a strong effect on the shape of the dose-response curve. We have revised the corresponding parts of the Discussion (lines 446-463) to clarify this point.

Regarding the comparison of dose-response curves of chloramphenicol (Cm) and kanamycin (Kan): we did not use aminoglycosides such as Kan in our assays because we could not observe a clear steady state of exponential growth for these antibiotics (the optical density growth curves were not exponential over several orders of magnitude, but exhibited different phases; this as also evident for Kan in Fig. 2A of Tan *et al.*). Therefore, we could not rigorously quantify the growth rate, which is a prerequisite for our quantitative analysis of the dose-response curve. Note that the dose-response curves for Kan in Fig. 2 of Tan *et al.* have a more complicated (non-monotonic) shape. In quantitative experiments published by other groups, these dose-response curves have more conventional (monotonic) shapes and the dose-response curve for chloramphenicol is much less sharp than for kanamycin (see e.g. Fig. 1 in Greulich *et al.*, MSB, 2015); this is consistent with what our experiments with these antibiotics have invariably shown.

2. I have several issues with the model formulation: First, there is no justification for the equation chosen for Ceff (Line 130-132), and it seems to be not even the simplest way to capture what the authors want it to capture. E.g. why not just Ceff = Cext * [1 + alpha*g/g0]? Instead, the authors are saying Ceff = Cext * [1 + alpha - alpha*g/g0]. What is the justification or purpose of that extra alpha term? By definition, as written, alpha>0 will potentiate the effect of the Hill coefficient, and vice versa. Moreover, and more to the point, the authors argue that the antibiotic effect on growth rate accounts for the steepness of the DR (i.e., the effective hill coefficient). If so, their alternative formulation of growth rate as a function of [abx]

should not contain this Hill structure. If they had presented some biologically justified alternate formulation that did not rely on Hill equation, yet still displayed Hill-like dose response, that would be interesting and supportive - they could then argue that feedback etc is leading to Hill phenotype.

We agree that the minimal model in the original version of our manuscript was not helpful in this regard, and we have developed a more mechanistic model of resource allocation to capture these phenomena (see below). We have drastically cut the role of the original minimal model in the main text and now use it only to illustrate the general effect of feedback loops (negative and positive) on the shape of dose-response curves, regardless of the underlying mechanisms (Fig. 1B); this model could even be removed altogether, but we still find it useful to illustrate the effects of growth-mediated feedbacks.

Nevertheless, to answer the reviewer's question: We used the form $c_{\mathrm{eff}} = [1 + \alpha\,(1 - g/g_0)]c_{\mathrm{ext}}$ because in this way positive values of α correspond to positive feedback and negative values of α to negative feedback; moreover, in this form, $c_{\mathrm{eff}} = c_{\mathrm{ext}}$ for $g = g_0$, i.e., the parameter α does not affect the effective drug concentration at the reference growth rate g_0; this would be different for the functional form suggested by the reviewer. In the end, this is a matter of style that makes little difference.

More importantly, in part because of this important reviewer comment, we have included a new resource allocation model in the revised version (end of Results section and new Fig. 6). The formulation of this model does not contain the Hill structure to begin with. It describes the concentration of the target of trimethoprim, its interaction with this drug and regulation, as well as resource allocation under carbon limitation, and is thus biologically well justified. Although the model does not lend itself to a simple analytical solution for the dose-response curve that would correspond exactly to a Hill function, it does yield a Hill-like dose-response curve with similar steepness as observed experimentally (new Fig. 6). The change in steepness of the dose-response curve resulting from various experimental perturbations is also well captured by this model (new Fig. 6). For more details on the model and the changes we made in the manuscript to include it, see our response to the first point of reviewer 1 above.

3. The data in Fig. 1C certainly shows some amount of correlation, but the methods aren't clear to me. 78 strains were chosen arbitrarily - which strains were used? The authors claim that these strains are unrelated to the mechanism of drug action, yet TMP interferes in folate metabolism which both feeds into, and from, various other interrelated aspects of cell physiology that are likely impacted by many genes. Also, why was 30% of the reference strain chosen and not the IC50 concentration? It seems quite arbitrary.

There was clearly a misunderstanding here: Fig. 1C shows data for all viable genome-wide gene deletion strains (almost 4,000 strains), not 78 arbitrarily chosen strains. We agree that some of these genes are specifically related to the mode of action of the drug. However, it is not reasonable to assume that the majority of genes have a specific effect on the mode of action of TMP. A general correlation as in Fig. 1C therefore likely reflects nonspecific effects of the change in drug-free growth rate resulting from the gene deletions. Since this growth-rate dependence of TMP efficacy is central to our main results, we validated this nonspecific effect of changes in drug-free growth rate on TMP susceptibility using three additional independent approaches (Fig. 2). Note that 78 arbitrarily chosen gene deletion strains were used only for the full dose-response curve measurements (Supplementary Fig. 2), which are a relatively minor complement to the main data shown in Fig. 1C and Supplementary Fig. 1 (see also response to the reviewer's next point). We have revised the main text (lines 152-155) and the legend of Fig. 1 to better explain this and make unmistakably clear that Fig. 1C shows genome-wide data.

The 30% inhibition used is a compromise that ensures that (a) most gene deletion strains exhibit significantly reduced growth compared to no drug and (b) most gene deletion strains that are more sensitive to the antibiotic than the reference strain still grow exponentially, allowing quantitative analysis

(a much larger fraction of the gene-deletion strains barely grow at the wild-type IC_{50}). We now explain this point in lines 155-159.

4. Data in supplementary Figure 2 is perhaps the least visually compelling of any trend between IC50 and growth rate. First, there is no obvious relationship for TMP data compared to the other drugs presented; also that TMP is statistically significant when CHL is not, despite the two having similarly low correlations, seems odd. Even so, I am not convinced that the statistical significance, in this case, is biologically meaningful.

The IC_{50} data shown in Supplementary Fig. 2 are a relatively minor complement to the main data shown in Fig. 1C and Supplementary Fig. 1 and are not essential for our main conclusions. The number of data points is necessarily lower here because growth rate measurements in concentration gradients with at least ten different concentrations are required for each strain to obtain an accurate dose-response curve and to quantify the IC_{50} (i.e. an order of magnitude more measurements per strain than in Fig. 1C). The reviewer is correct that fewer data points generally make such correlations less visually compelling than, for example, the one shown in Fig. 1C. However, it is common practice to analyze the significance of such correlations quantitatively to avoid biased conclusions that result from simply looking at the scatter plots. In this case, this standard statistical analysis shows that (a) the (anti-)correlation for TMP is indeed low but stronger than for CHL ($\rho_S = -0.27$ for TMP versus $\rho_S = -0.22$ for CHL) and (b) it is significantly different from zero for TMP (p=0.019) at the common 5% level, whereas this is not the case for CHL (p=0.071). The correlations for all other antibiotics studied are even weaker and none of them are significant (Supplementary Fig. 2).

We have toned down the conclusion from these data in the main text (lines 171-175). We could leave them out altogether if it satisfies the reviewer, but so far we are not convinced that there is any problem with these data or their analysis.

5. The key data in this paper is presented in Fig 2b; however, at aMG/Glu >= 10 it looks like the growth rates are not even monotonically decreasing with increasing TMP, which does not necessarily suggest to me that the dose responses are shallower. The authors would likely need a denser range of Amg/Glu ratios on the range [0 5]. Also, the heatmap itself makes it quite challenging to interpret the trends - the authors should plot multiple growth vs TMP dose response lines (instead of this heatmap).

We agree that this non-monotonic dependence of growth rate on TMP concentration is quite striking. Therefore, we devoted two paragraphs to it in the main text (lines 202-206 of the original manuscript; lines 228-237 in the revised version) and at the end of the Discussion (lines 387-402 of the original manuscript; lines 485-503 in the revised version). We further agree that the shape of the TMP dose-response curve in this region is more complicated and difficult to interpret. This phenomenon occurs at high αMG/glucose ratios where carbon limitation becomes so strong that growth approaches zero. This is an extreme situation in which virtually no growth occurs in the absence of TMP but the addition of this antibiotic promotes growth; we agree with the reviewer that it is not helpful to describe such a qualitatively different effect, in which an antibiotic promotes growth, as a change in the shape of the dose-response curve. Therefore, we limited the discussion of the effects of drug-free growth rate on the quantitative shape of the TMP dose-response curve to lower αMG/glucose ratios. However, it should be noted that this phenomenon is qualitatively consistent with the TMP-specific mechanism we identified: *folA* expression is greatly increased at low drug-free growth rates (Fig. 4C), leading to a situation in which blocking a fraction of FolA in the cell by TMP promotes growth, probably by rebalancing metabolic resources in the cell. Direct overexpression of *folA* results in a similar rescue effect by TMP, further supporting this scenario (Supplementary Fig. 10).

We have revised the main text to more explicitly state that the TMP dose-response curve in this regime has a very unusual shape and to explain this phenomenon in more detail (lines 230-237 in Results and lines 485-491 in Discussion). To highlight this unusual shape more clearly, we have included an inset in Fig. 2B showing a vertical section through the heat map at a higher αMG/glucose ratio. We have also added the new Supplementary Fig. 15, which shows, among other things, the data from Fig. 2B,E,H as multiple growth-vs-TMP concentration dose-response lines.

6. Lines 228-232, - that modulating growth by temperature does not result in the same phenotype raises further questions about the central claim. It makes sense that the non-normalized growth rates decrease with decreasing temp (Fig S8 LHS), but the fact that the normalized curves (RHS) overlap counteracts their premise that lower growth == shallower curves (eg Discussion lines 347-348). In fact, it suggests a more complex explanation (perhaps circling back to metabolism as of TMP as I suspect).

We thank the reviewer for pointing this out. We understand that it was not sufficiently explained. Reviewer 1 raised essentially the same issue. It is well established that changing growth rate by changing temperature generally does not produce the same phenotypes as other means of changing growth rate. For a detailed explanation of the reasons and the corresponding changes we have made to the manuscript to clarify this point for a broader audience, please see our response to point 4 of reviewer 1 above.

7. A great deal of relevant literature was not referenced, including Lee et al in PNAS 2018 and Lopatkin et al in Nature Microbiology 2019. Both cases investigate growth specific quantitative relationships of antibiotic and susceptibility.

We agree that these two papers are interesting and relevant to our work and thank the reviewer for pointing them out. Note that we had already cited the Lee *et al.*, PNAS, 2018 (line 89 of the original manuscript; lines 102 and 226 of the revised manuscript). We now also cite Lopatkin *et al.*, Nature Microbiology, 2019) in the Introduction (line 108).

8. The data presented overall lacked transparency - most figures reported normalized values on both axes. For example, why did the x-axis need to be normalized in Fig. 1C? Also, the x-axis was scaled in fig. 1A that made it difficult to interpret the trends. At the minimum, in all cases, the raw data should be presented in the supplementary information.

We normalized (or scaled) some of the data for presentation purposes. For example, we normalized the x-axis in Fig. 1C to treat both axes in this graph consistently (note that the ranges of values on the x- and y-axis are also identical; it is relatively common to normalize the response on the y-axis in dose-response curves to the growth rate in the absence of the drug). This may be partly a matter of taste. The x-axis in Fig. 1A was scaled for each drug so that the steepness of the different dose-response curves (which is independent of this scaling) is clearly visible and increases from left to right; the absolute concentrations of the different antibiotics are less relevant to this specific point.

Nevertheless, following the reviewer suggestion, we either changed the axes in all main figures to non-normalized values where possible without complicating the interpretation of the figure (Fig. 1A,C), or added the same plots using non-normalized values as supplementary figures (Fig. 3A,C,E; new Supplementary Figures 14 and 15A,E,I). The normalized *folA* expression levels in Fig. 4 are an exception – here, the absolute fluorescence intensities we measured depend on the instrument and detector settings and are virtually meaningless, so we kept the normalized values only (they correspond to fold-change in expression). We have also retained the scaling of the x-axis in Fig. 1A to clearly show the differences in dose-response curve steepness, but we now also show the unscaled data in the new Supplementary Fig. 13.

9. The authors argue that "TMP lowers growth, which in turn weakens the inhibitory effect of the drug". If this were the case, wouldn't you expect growth rate to oscillate with time -- TMP lowers growth -> less potent TMP == faster growth -> faster growth == more potent TMP.

This is an interesting idea. We agree that a negative feedback loop with a time delay can generally produce oscillations. However, we think that such oscillations would most likely not be detectable at the population level, since this phenomenon may not be synchronized across the population. Such oscillations in growth rate might occur at the single-cell level but would be difficult to detect on top of the cell cycle and other fluctuations (precise measurements of the instantaneous growth rate of individual bacteria are technically extremely challenging, see e.g. Godin *et al.*, Nature Methods, 2010; DOI: 10.1038/nmeth.1452). Further, the time scale (period) of such an oscillation is unclear and might be too short to be easily detectable. For the revised manuscript, we have performed single-cell time-lapse imaging experiments in which TMP is suddenly added to an *E. coli* population growing in a microfluidic chamber (see response to point 3 of reviewer 1 and the new Supplementary Fig. 16) but we have not observed such oscillations.

Nonetheless, it is possible that this effect occurs and it might be detectable with more advanced techniques. Therefore, we now mention this interesting possibility in the Discussion (lines 464-472).

10. Figure 5 lacks sufficient controls. In particular, they don't account for the increased burden of IPTG expression on growth rate in the absence of drug, which could also influence antibiotic susceptibility. Also lines 171-173, the sentence starting with "Notably..." is not supported by their own data.

It is correct that IPTG-induced expression in the experiment shown in Fig. 5 has a slight effect on drug-free growth rate (~20% compared with wild type), but this is relatively unproblematic for the purpose of this experiment. First, this effect is the same for the *constant FolA* and *inverted FolA regulation* lines, and the observed increase in dose-sensitivity is huge. Thus, the data in Fig. 5 show that breaking the negative feedback loop changes the shape of the dose-response curve regardless of the comparison with the absolute growth rate of the wild type. Second, to further support this point, we compared the effects shown in Fig. 5 with those that result when growth rate is altered to a similar extent in the wild type; here, a ~20% reduction in growth rate by glucose limitation only increases dose-sensitivity to $n \approx 1.5$ (Fig. 3B); the changes in dose-sensitivity are even weaker when the growth rate is reduced by approximately 20% by overexpressing a gratuitous protein (Fig. 3D) or by changing the carbon source (Fig. 3F). Overall, the dose-sensitivity that would result from the slight decrease in growth rate alone is clearly lower than that observed with constant FolA ($n \approx 2.0$) and with inverted FolA regulation ($n \approx 5.0$) in Fig. 5. We now explain this issue in more detail and highlight the comparison with these relevant controls in the main text (lines 378-383).

Regarding lines 171-173: The point of this statement was to indicate that there may be a more general trend that the strength of the negative correlations as in Fig. 1C,D tends to coincide with the steepness of the dose-response curve; we do not claim that this is generally or even strictly true for all antibiotics. Other factors can certainly contribute to the shape of the dose-response curve, which may override the growth-mediated feedback we focus on in this work. That said, our data are consistent with such a more general trend: TMP shows by far the strongest anti-correlation in Fig. 1D and the shallowest dose-response curve (Fig. 1A); MEC has one of the lowest anti-correlations and the steepest dose-response curve; TET, CHL have intermediate values for both the anti-correlation and the steepness of their dose-response curve; NIT and CPR do not follow this trend, but neither do they deviate much from it. It is hard to strengthen this point further because the number of truly distinct antibiotics one can use in such experiments (which rely on quantitative growth rate measurements) is relatively low; the resulting small number of data points essentially rules out the possibility of a statistically significant correlation here from the outset. In principle, this analysis could be performed for a larger number of antibiotics, but the

problem remains that there is only a fairly limited number of different modes of action for antibiotics, most of which are already represented by the drugs used in Fig. 1.

To address this point, we have further toned down the statement in this sentence; we now explicitly say that this correlation is not significant, and briefly explain the considerations above (lines 183-189). We could also delete this sentence entirely if the reviewer feels it is important. We have further revised the Discussion (lines 446-463) to clarify that there are indications of a more general role of growth-mediated feedback in antibiotic dose-responses, but that this remains to be studied in detail for most antibiotics.

RE: MSB-2021-10490RR-Q, Growth-mediated negative feedback shapes quantitative antibiotic response

Thank you again for submitting your work to Molecular Systems Biology. I would like to apologise for the delay in sending you a decision on your manuscript, which was due the fact that I was out of office during the last two weeks. We have now heard back from the two reviewers (previous reviewers #1 and #3) who were asked to evaluate your study. As you will see below, the reviewers think that the study has improved as a result of the performed revisions. Reviewer #1 lists a few remaining concerns related to the model(s), which we would ask you to address in a final round of revision.

We would also ask you to address some editorial issues listed below.

Reviewer #1:

The authors have done a substantial job in addressing many of my raised comments initially. In particular, they clarified the calculation of growth rates and how they were able to neglect death rates. This clarification makes it easier to interpret the experimental data.

Given this, I believe that the core conclusions are sound. However, there are two lingering points that I hope the authors will address. First, I don't understand why the authors would like to keep the original simple model. If it's purely used for data fitting, it's unnecessarily complicated (relative to a Hill equation, which they used). If it's intended to illustrate a mechanism, it's misleading and at least insufficiently justfied. I would suggest the authors remove it.

I can see the rationale of the new model, which however it's a bit over-complicated given the mechanism they proposed. Again, as I mentioned in my initial review, I actually find the basic idea very intuitive, which was obscured by the subsequent presentation. This issue continues to persist (to a lesser degree), which would reduce the general accessibility of the story. The authors might consider using a simple kinetic model to implement their mechanism or, better clarify what new insights were learned from the current model (the more complicated one).

On a more subtle point, it is unclear whether either of the proposed models can explain the non-monotonic dose responses under certain conditions (Figure 2)

Reviewer #3:

The authors have adequately addressed my comments and is suitable for publication

Point-by-point response

[The line numbers below refer to the DOCX file of the revised version showing the tracked changes (with "All Markup").]

Reviewer #1:

The authors have done a substantial job in addressing many of my raised comments initially. In particular, they clarified the calculation of growth rates and how they were able to neglect death rates. This clarification makes it easier to interpret the experimental data.

Given this, I believe that the core conclusions are sound. However, there are two lingering points that I hope the authors will address. First, I don't understand why the authors would like to keep the original simple model. If it's purely used for data fitting, it's unnecessarily complicated (relative to a Hill equation, which they used). If it's intended to illustrate a mechanism, it's misleading and at least insufficiently justfied. I would suggest the authors remove it.

We thank the reviewer for their positive assessment and for the constructive criticism. Following the reviewer's suggestion, we have reconsidered the relevance of the original model. We agree that, after the addition of the new model and other changes made in the manuscript, it is no longer necessary and might make our work less accessible. Therefore, we have completely removed this model from our manuscript (lines 140-143 and 779-798). Accordingly, we changed Fig. 1B into a schematic that illustrates the general role of growth-mediated feedback loops in shaping the dose-response curve.

I can see the rationale of the new model, which however it's a bit over-complicated given the mechanism they proposed. Again, as I mentioned in my initial review, I actually find the basic idea very intuitive, which was obscured by the subsequent presentation. This issue continues to persist (to a lesser degree), which would reduce the general accessibility of the story. The authors might consider using a simple kinetic model to implement their mechanism or, better clarify what new insights were learned from the current model (the more complicated one).

We agree with the reviewer that the basic idea of this work should be quite intuitive for most readers. However, we do not feel that the new model is overly complicated. It is a relatively straightforward application of the CAFBA framework, which is state-of-the-art for modeling resource allocation in bacteria. The model connects four different empirically observed variables: a (αMG/glucose ratio), c (trimethoprim concentration), λ (growth rate), and χ_{FolA} (fluorescence signal of FolA-GFP), which would have to be included in any model that is directly compared to our experimental data. After suitable rescaling of these variables, the model has only a single meaningful fit parameter, which supports its relative simplicity. For these reasons, we decided against replacing this model with a simpler kinetic model.

However, we have made an effort to better explain this model and the insights gained from it. Specifically, we have added several sentences to explain the connection of this model to the basic intuition (lines 409-415 and 426-434). To optimize the presentation, we have made smaller revisions throughout the part describing the model (lines 400-444) and in the Discussion that refers to the model (lines 491-510). We have also added a sentence at the end of the Results section (lines 441-444) to sum up the insights gained from this model. To clarify that the model has only a single meaningful fit parameter, we have added a short part in the Materials and Methods (lines 849-861).

On a more subtle point, it is unclear whether either of the proposed models can explain the non-monotonic dose responses under certain conditions (Figure 2)

This is a good point. The non-monotonic dose-response curves are currently not explained by the model we propose. In our experiments, this phenomenon occurs only under extreme carbon limitation that drastically lowers growth even in the absence of antibiotic. This situation is likely too far away from the physiological conditions assumed in the model (where e.g. bacterial growth laws hold, which may no longer be the case under extreme conditions). We have added an explanation of this point in lines 506-509.

Reviewer #3:

The authors have adequately addressed my comments and is suitable for publication

We thank the reviewer for re-evaluating our manuscript. We are very pleased that the reviewer feels we have adequately addressed their previous comments and now supports publication.

Manuscript number: MSB-2021-10490RRR, Growth-mediated negative feedback shapes quantitative antibiotic response

Thank you for performing the requested minor revisions. We are now satisfied with the modifications made and I am pleased to inform you that your paper has been accepted for publication.

EMBO Press Author Checklist

Corresponding Author Name: Tobias Bollenbach
Journal Submitted to: Molecular Systems Biology
Manuscript Number: MSB-2021-10490RR-Q

USEFUL LINKS FOR COMPLETING THIS FORM
The EMBO Journal - Author Guidelines
EMBO Reports - Author Guidelines
Molecular Systems Biology - Author Guidelines
EMBO Molecular Medicine - Author Guidelines

Reporting Checklist for Life Science Articles (updated January
This checklist is adapted from Materials Design Analysis Reporting (MDAR) Checklist for Authors. MDAR establishes a minimum set of requirements in transparent reporting in the life sciences (see Statement of Task: 10.31222/osf.io/9sm4x). Please follow the journal's guidelines in preparing your manuscript.
Please note that a copy of this checklist will be published alongside your article.

Abridged guidelines for figures
1. Data
The data shown in figures should satisfy the following conditions:
- the data were obtained and processed according to the field's best practice and are presented to reflect the results of the experiments in an accurate and unbiased manner.
- ideally, figure panels should include only measurements that are directly comparable to each other and obtained with the same assay.
- plots include clearly labeled error bars for independent experiments and sample sizes. Unless justified, error bars should not be shown for technical
- if n<5, the individual data points from each experiment should be plotted. Any statistical test employed should be justified.
- Source Data should be included to report the data underlying figures according to the guidelines set out in the authorship guidelines on Data

2. Captions
Each figure caption should contain the following information, for each panel where they are relevant:
- a specification of the experimental system investigated (eg cell line, species name).
- the assay(s) and method(s) used to carry out the reported observations and measurements.
- an explicit mention of the biological and chemical entity(ies) that are being measured.
- an explicit mention of the biological and chemical entity(ies) that are altered/varied/perturbed in a controlled manner.
- the exact sample size (n) for each experimental group/condition, given as a number, not a range;
- a description of the sample collection allowing the reader to understand whether the samples represent technical or biological replicates (including how many animals, litters, cultures, etc.).
- a statement of how many times the experiment shown was independently replicated in the laboratory.
- definitions of statistical methods and measures:
  - common tests, such as t-test (please specify whether paired vs. unpaired), simple χ2 tests, Wilcoxon and Mann-Whitney tests, can be unambiguously identified by name only, but more complex techniques should be described in the methods section;
  - are tests one-sided or two-sided?
  - are there adjustments for multiple comparisons?
  - exact statistical test results, e.g., P values = x but not P values < x;
  - definition of 'center values' as median or average;
  - definition of error bars as s.d. or s.e.m.

Please complete ALL of the questions below.
Select "Not Applicable" only when the requested information is not relevant for your study.

Materials

Newly Created Materials	Information included in the manuscript?	In which section is the information available? (Reagents and Tools Table, Materials and Methods, Figures, Data Availability Section)
New materials and reagents need to be available; do any restrictions apply?	Yes	Reagents and Tools Table, Materials and Methods

Antibodies	Information included in the manuscript?	In which section is the information available? (Reagents and Tools Table, Materials and Methods, Figures, Data Availability Section)
For **antibodies** provide the following information:<br>- Commercial antibodies: RRID (if possible) or supplier name, catalogue number and or/clone number<br>- Non-commercial: RRID or citation	Not Applicable	

DNA and RNA sequences	Information included in the manuscript?	In which section is the information available? (Reagents and Tools Table, Materials and Methods, Figures, Data Availability Section)
**Short novel DNA or RNA including primers, probes**: provide the sequences.	Yes	Reagents and Tools Table, Table EV1

Cell materials	Information included in the manuscript?	In which section is the information available? (Reagents and Tools Table, Materials and Methods, Figures, Data Availability Section)
**Cell lines:** Provide species information, strain. Provide accession number in repository **OR** supplier name, catalog number, clone number, and/**OR** RRID.	Not Applicable	
**Primary cultures:** Provide species, strain, sex of origin, genetic modification status.	Not Applicable	
Report if the cell lines were recently **authenticated** (e.g., by STR profiling) and tested for mycoplasma contamination.	Not Applicable	

Experimental animals	Information included in the manuscript?	In which section is the information available? (Reagents and Tools Table, Materials and Methods, Figures, Data Availability Section)
**Laboratory animals or Model organisms:** Provide species, strain, sex, age, genetic modification status. Provide accession number in repository **OR** supplier name, catalog number, clone number, **OR** RRID.	Not Applicable	
**Animal observed in or captured from the field:** Provide species, sex, and age where possible**.**	Not Applicable	
Please detail **housing and husbandry conditions**.	Not Applicable	

Plants and microbes	Information included in the manuscript?	In which section is the information available? (Reagents and Tools Table, Materials and Methods, Figures, Data Availability Section)
**Plants:** provide species and strain, ecotype and cultivar where relevant, unique accession number if available, and source (including location for collected wild specimens).	Not Applicable	
**Microbes:** provide species and strain, unique accession number if available, and source.	Yes	Reagents and Tools Table, Materials and Methods

Human research participants	Information included in the manuscript?	In which section is the information available? (Reagents and Tools Table, Materials and Methods, Figures, Data Availability Section)
If collected and within the bounds of privacy constraints report on age, sex and gender or ethnicity for all study participants.	Not Applicable	

Core facilities	Information included in the manuscript?	In which section is the information available? (Reagents and Tools Table, Materials and Methods, Figures, Data Availability Section)
If your work benefited from core facilities, was their service mentioned in the acknowledgments section?	Yes	Materials and Methods

Design

Study protocol	Information included in the manuscript?	In which section is the information available? (Reagents and Tools Table, Materials and Methods, Figures, Data Availability Section)
If study protocol has been **pre-registered, provide DOI in the manuscript**. For clinical trials, provide the trial registration number **OR** cite DOI.	Not Applicable	
Report the **clinical trial registration number** (at ClinicalTrials.gov or equivalent), where applicable.	Not Applicable	

Laboratory protocol	Information included in the manuscript?	In which section is the information available? (Reagents and Tools Table, Materials and Methods, Figures, Data Availability Section)
Provide DOI OR other citation details if **external detailed step-by-step protocols** are available.	Not Applicable	

Experimental study design and statistics	Information included in the manuscript?	In which section is the information available? (Reagents and Tools Table, Materials and Methods, Figures, Data Availability Section)
Include a statement about **sample size** estimate even if no statistical methods were used.	Not Applicable	
Were any steps taken to minimize the effects of subjective bias when allocating animals/samples to treatment (e.g. **randomization procedure**)? If yes, have they been described?	Not Applicable	
Include a statement about **blinding** even if no blinding was done.	Not Applicable	
Describe **inclusion/exclusion criteria** if samples or animals were excluded from the analysis. Were the criteria pre-established?<br><br>If sample or data points were omitted from analysis, report if this was due to attrition or intentional exclusion and provide justification.	Not Applicable	
For every figure, are **statistical tests** justified as appropriate? Do the data meet the assumptions of the tests (e.g., normal distribution)? Describe any methods used to assess it. Is there an estimate of variation within each group of data? Is the variance similar between the groups that are being statistically compared?	Yes	Figures

Sample definition and in-laboratory replication	Information included in the manuscript?	In which section is the information available? (Reagents and Tools Table, Materials and Methods, Figures, Data Availability Section)
In the figure legends: state number of times the experiment was **replicated** in laboratory.	Yes	Figures, Materials and Methods
In the figure legends: define whether data describe **technical or biological replicates**.	Yes	Figures

Ethics

Ethics	Information included in the manuscript?	In which section is the information available? (Reagents and Tools Table, Materials and Methods, Figures, Data Availability Section)
Studies involving **human participants**: State details of **authority granting ethics approval** (IRB or equivalent committee(s), provide reference number for approval.	Not Applicable	
Studies involving **human participants**: Include a statement confirming that **informed consent** was obtained from all subjects and that the experiments conformed to the principles set out in the WMA Declaration of Helsinki and the Department of Health and Human Services Belmont Report.	Not Applicable	
Studies involving **human participants:** For publication of **patient photos**, include a statement confirming that consent to publish was obtained.	Not Applicable	
Studies involving experimental **animals**: State details of **authority granting ethics approva**l (IRB or equivalent committee(s), provide reference number for approval. Include a statement of compliance with ethical regulations.	Not Applicable	
Studies involving **specimen and field samples:** State if relevant **permits** obtained, provide details of authority approving study; if none were required, explain why.	Not Applicable	

Dual Use Research of Concern (DURC)	Information included in the manuscript?	In which section is the information available? (Reagents and Tools Table, Materials and Methods, Figures, Data Availability Section)
Could your study fall under dual use research restrictions? Please check biosecurity documents and list of **select agents and toxins** (CDC): https://www.selectagents.gov/sat/list.htm	Not Applicable	
If you used a select agent, is the security level of the lab appropriate and reported in the manuscript?	Not Applicable	
If a study is subject to dual use research of concern regulations, is the name of the **authority granting approval and reference number** for the regulatory approval provided in the manuscript?	Not Applicable	

Reporting

The MDAR framework recommends adoption of discipline-specific guidelines, established and endorsed through community initiatives. Journals have their own policy about requiring specific guidelines and recommendations to complement MDAR.

Adherence to community standards	Information included in the manuscript?	In which section is the information available? (Reagents and Tools Table, Materials and Methods, Figures, Data Availability Section)
State if relevant guidelines or checklists (e.g., **ICMJE, MIBBI, ARRIVE, PRISMA**) have been followed or provided.	Not Applicable	
For **tumor marker prognostic studies,** we recommend that you follow the **REMARK** reporting guidelines (see link list at top right). See author guidelines, under 'Reporting Guidelines'. Please confirm you have followed these guidelines.	Not Applicable	
For **phase II and III randomized controlled trials**, please refer to the **CONSORT** flow diagram (see link list at top right) and submit the CONSORT checklist (see link list at top right) with your submission. See author guidelines, under 'Reporting Guidelines'. Please confirm you have submitted this list.	Not Applicable	

Data Availability

Data availability	Information included in the manuscript?	In which section is the information available? (Reagents and Tools Table, Materials and Methods, Figures, Data Availability Section)
Have **primary datasets** been deposited according to the journal's guidelines (see 'Data Deposition' section) and the respective accession numbers provided in the Data Availability Section?	Not Applicable	
Were **human clinical and genomic datasets** deposited in a public access-controlled repository in accordance to ethical obligations to the patients and to the applicable consent agreement?	Not Applicable	
Are **computational models** that are central and integral to a study available without restrictions in a machine-readable form? Were the relevant accession numbers or links provided?	Not Applicable	
If publicly available data were reused, provide the respective **data citations in the reference list**.	Yes	
